# CASP-Model Sepsis Triggers Systemic Innate Immune Responses Revealed by the Systems-Level Signaling Pathways

**DOI:** 10.3389/fimmu.2022.907646

**Published:** 2022-06-14

**Authors:** Hannan Ai, Bizhou Li, Fanmei Meng, Yuncan Ai

**Affiliations:** ^1^ State Key Laboratory for Biocontrol, School of Life Sciences, Sun Yat-sen University, Guangzhou, China; ^2^ Department of Electrical and Computer Engineering, The Grainger College of Engineering, University of Illinois at Urbana-Champaign, Urbana, IL, United States; ^3^ National Center for Quality Supervision and Inspection of Automatic Equipment, National Center for Testing and Evaluation of Robots (Guangzhou), CRAT, SINOMACH-IT, Guangzhou, China; ^4^ The Second Affiliated Hospital, Guangdong Provincial Key Laboratory of Allergy & Clinical Immunology, Center for Inflammation, Immunity & Immune-mediated Disease, Sino-French Hoffmann Institute, Guangzhou Medical University, Guangzhou, China

**Keywords:** microbial immunology, colon ascendens stent peritonitis (CASP), signaling pathways, innate immune responses, transcriptional regulation network, innate immunity, systems biology, bioinformatics

## Abstract

Colon ascendens stent peritonitis (CASP) surgery induces a leakage of intestinal contents which may cause polymicrobial sepsis related to post-operative failure of remote multi-organs (including kidney, liver, lung and heart) and possible death from systemic syndromes. Mechanisms underlying such phenomena remain unclear. This article aims to elucidate the mechanisms underlying the CASP-model sepsis by analyzing real-world GEO data (GSE24327_A, B and C) generated from mice spleen 12 hours after a CASP-surgery in septic MyD88-deficient and wildtype mice, compared with untreated wildtype mice. Firstly, we identify and characterize 21 KO MyD88-associated signaling pathways, on which true key regulators (including ligands, receptors, adaptors, transducers, transcriptional factors and cytokines) are marked, which were coordinately, significantly, and differentially expressed at the systems-level, thus providing massive potential biomarkers that warrant experimental validations in the future. Secondly, we observe the full range of polymicrobial (viral, bacterial, and parasitic) sepsis triggered by the CASP-surgery by comparing the coordinated up- or down-regulations of true regulators among the experimental treatments born by the three data under study. Finally, we discuss the observed phenomena of “systemic syndrome”, “cytokine storm” and “KO MyD88 attenuation”, as well as the proposed hypothesis of “spleen-mediated immune-cell infiltration”. Together, our results provide novel insights into a better understanding of innate immune responses triggered by the CASP-model sepsis in both wildtype and MyD88-deficient mice at the systems-level in a broader vision. This may serve as a model for humans and ultimately guide formulating the research paradigms and composite strategies for the early diagnosis and prevention of sepsis.

## Introduction

Infectious diseases have led mortality rates worldwide. Sepsis is the prevalent cause of death in the intensive care unit (1–3). Sepsis occurs when the immune response to an infection becomes dysregulated, resulting in life-threatening organ dysfunction (4). Sepsis is scientifically termed as a systemic syndrome of persistent and overwhelming inflammation that occurs at the early stage of infection (4–6). It has been perceived that bacterial infection may cause sepsis (7–10); while viral infection may produce cytokine storm (11–13); and parasitic infection may also yield sepsis (10). It is well-known that Toll-like receptors (TLRs) including TLR2, TLR4 and TLR9 contribute to polymicrobial sepsis (14, 15). These TLRs play crucial roles in signaling *via* the common myeloid differentiation factor 88 (MyD88)-dependent pathways (5, 16–19). TLR4 also signals through MyD88-independent but TIR domain-containing adaptor inducing IFNβ-mediated transcription factor (Trif)-dependent pathway (10, 17, 18, 20). Based on the bench-experiments of multiplex immunoassay and flow cytometry analysis in deficient mice, the cytokine production, neutrophil migration, and phagocytic function have been suggested to play critical roles in the pathogenesis of sepsis (21–23). Cytokines and chemokines including IL-6 (24), IL-10 (7, 24), CD14 (17), to name a few, were suggested as potential biomarkers for early diagnosis (before organ dysfunction occurs) of sepsis (25). But due to evolutionary discordance between human and mouse innate immune signaling (26), these may not necessarily be true biomarkers (4). 

The colon ascendens stent peritonitis (CASP) surgery induces a leakage of intestinal contents, causing polymicrobial sepsis related to post-operative failure of multi-organs (including heart, spleen, kidney, liver and lung) and possible death from syndromes (4). The CASP-model sepsis is a standardized approach to understanding pathogenesis of polymicrobial sepsis (8, 23, 24, 27–32). Deficient mice provide ideal models to investigate the roles of specific candidate biomarkers (24, 33). Transcriptomic analysis of mRNAs profiling on microarray, coupled with bioinformatics analysis, is powerful to decipher true key regulators through mapping them onto true KEGG pathways (34). In fact, KEGG pathways are manually curated based on pre-validated experimental and/or literature evidence (35, 36). The true key regulators identified by pathway enrichment analysis are rendered on KEGG pathways, which not only yields high efficacy of identification, but also offers true broader vision beyond traditional detection approaches (10, 24). As an imminent frontier in the field, top-down data-driven pathway enrichment analysis should become the advanced approach to investigate sepsis pathogenesis.

The real-world GSE24327 data were generated from samples recovered from spleens 12 hours after the CASP-model sepsis in MyD88-deficient and wildtype mice (24). The previous bench-experiments assessed the expression levels of regulators, including MFI (CD11b), CXCL10, MIP-2 (CXCL2), RANTES (CCL5), IL-17, IL-6, IL-10, IL-12, Ifng, Ifnb, IL-15, Ifit3, Ccl12, Rsad2 and IFNβ, by which a putative signaling pathway was determined, responsible for the innate immune responses triggered by the CASP-surgery (24). These data were not subjected to bioinformatics analysis like our perspectives and we are curious about additional occurences beyond the documented stories (24).

This study aims to elucidate mechanisms underlying the CASP-model sepsis at the systems-level through analyzing GSE24327 data (24) *via* top-down data-driven analysis. We discuss the observed phenomena of “systemic syndrome”, “cytokine storm”, and “KO MyD88 attenuation”, as well as the proposed hypothesis of “spleen-mediated immune-cell infiltration”. Our results provide novel insights into a better understanding of the CASP-model sepsis in mice, which may serve as a model for humans, to ultimately guide formulating the research paradigms and composite strategies for early diagnosis and prevention of severe sepsis.

## Materials and Methods

### Data Collection From GEO Database and Assignment of Subtype Data

We downloaded GSE24327 data from https://ncbi.nlm.nih.gov/geo/GSE24327. To select the significantly, differentially expressed genes (DEGs), we designed three subtypes of data according to the original bench-experiments (24): GSE24327_A (a. septic KO MyD88 *vs*. septic WT) for comparing septic null (*Myd88* ^–/–^) with septic wildtype mice; GSE24327_B (b. septic KO MyD88 *vs*. untreated WT) for comparing septic null (*Myd88* ^–/–^) with untreated wildtype mice; and GSE24327_C (c. septic WT *vs*. untreated WT) for comparing septic wildtype with untreated wildtype mice.

### Automatic Identification of Signaling Pathways With the PathwayKO Package

The PathwayKO package is available at https://github.com/allenaigit/pathwayko/. Briefly, the PathwayKO package is systems software, rather than a standalone application algorithm, which comprises multiple modules with diverse dependencies, allowing users to conduct integrated processes in a pipeline fashion, such as (I) preprocessing, (II) ROC-AUC calculating, (III) statistics analyzing, and (IV) visualizing. Users who might lack knowledge and/or experience would seek help from professionals and consult the offered users’ manual to understand our interpretations. It currently incorporates the state-of-the-art methods of pathway analysis, statistics analysis, and visualizing analysis. The non-topology-based methods of pathway analysis include SAFE (37), GSEA (38), GSA (39) and PADOG (40). The topology-based methods of pathway analysis include ROntoTools_PE (41), ROntoTools_pDIS (42) and SPIA (43). The statistics analysis methods include the changepoint package (44) and the pROC package (45). The visualizing analysis methods include the pROC package (45) and the Pathview package (34). Certain metrics are important for statistics comparison. The Youden’s best *p*-value (46) is employed to choose the best point on the ROC curve. AUC (with 95% CI, confidence interval) for a full area under the entire ROC-curve, and partial AUCs (pAUC_SP and pAUC_SE) for regions focusing on the 90–100% of specificity and sensitivity, in both original and corrected formats (47), are deployed to rank methods. The Adj.*p*-value is used to control the false discovery rate (FDR) for multiple testing (48, 49). Most importantly, the PathwayKO package has an advantage to conduct automatic selection of differentially expressed genes (DEGs) because it adapts the change-point method (44) based on HES threshold (50) that are automatically determined on the distribution of edge scores of each data, into which the fold-change and probability values of genes at the systems-level have been integrated.

To install the PathwayKO package, one needs to create and enter into a user directory (PathwayKO_demo_run), and type command lines:

$ cd/home/PathwayKO_demo_run
PathwayKO_demo_run$ R
> library(devtools)
> devtools::install_github(“allenaigit/pathwayko”)

To get started with the package, the user needs to run a demo:

$ cd/home/PathwayKO_demo_run
PathwayKO_demo_run$ R
> library(pathwayko)
> pathwayko_demo()

Batch-execution is conducted with parallel computations, which produces resulting output directories, each containing a group of desired output-files. The user completed a desired job by following instructions in the User’s manual, publicly available at https://github.com/allenaigit/pathwayko/tree/main/inst/docs/Users_manual.pdf.

### Estimation of Possible Infiltration of Core Immune Cells at the Systems-Level

We estimated the correlation between the expression level of key regulators and the infiltration level of core immune cells (CICs) (B, CD8+T, CD4+T, neutrophil, macrophage and dendritic cells) in human cancer cohorts by utilizing cohort database TIMER2.0 (https://cistrome.shinyapps.io/timer) (51).

## Results

### Automatic Selection of Coordinately, Significantly, Differentially Expressed Genes (DEGs)

**Figure 1:**
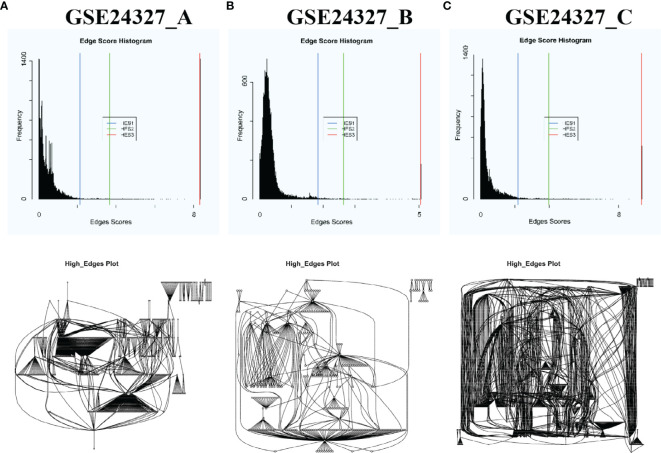
Automatic selection of DEGs with each HES1 threshold based on the distribution of edge scores and automatic construction of putative regulatory networks of DEGs of each data by using the PathwayKO package. Top-panel shows the landscape of true DEGs of each data. Bottom-panel displays the landscape of putative regulatory networks constituted by the true DEGs of each data. These landscapes drastically vary among data in a broader vision, reflecting real-world differences of the bench-experimental treatments: **(A)** septic KO MyD88 *vs*. septic wildtype (WT), **(B)** septic KO MyD88 *vs*. untreated WT, and **(C)** septic WT *vs*. untreated WT. See the main text.

Top-panel (**Figure 1**) displays the landscape of unbiased true DEGs automatically identified from GSE24327_A (408 DEGs), B (266 DEGs), and C (648 DEGs) data (**Supplementary Tables S1–S3**). Such true DEGs constitute putative regulatory networks, as shown by the landscapes in bottom-panel (**Figure 1**). Obviously, such true DEGs vary drastically among data, reflecting real-world variations among the bench-experimental treatments of (**A**), (**B**) and (**C**) under study. Note that **Figure 1** only shows the landscapes of these true DEGs (named as Edge Score Histogram) and their putative regulatory networks (named as High-Edges plot) in a broader vision at the systems-level. Each landscape may comprise hundreds or thousands of DEGs, which are unreadable by humans. Among such true DEGs, key regulators will be specifically identified, precisely located, and differentially marked on target signaling pathways *via* the subsequent analyses throughout the current study.

### Automatic Identification of the Entirety of KO MyD88-Associated Target Signaling Pathways

**Figure 2:**
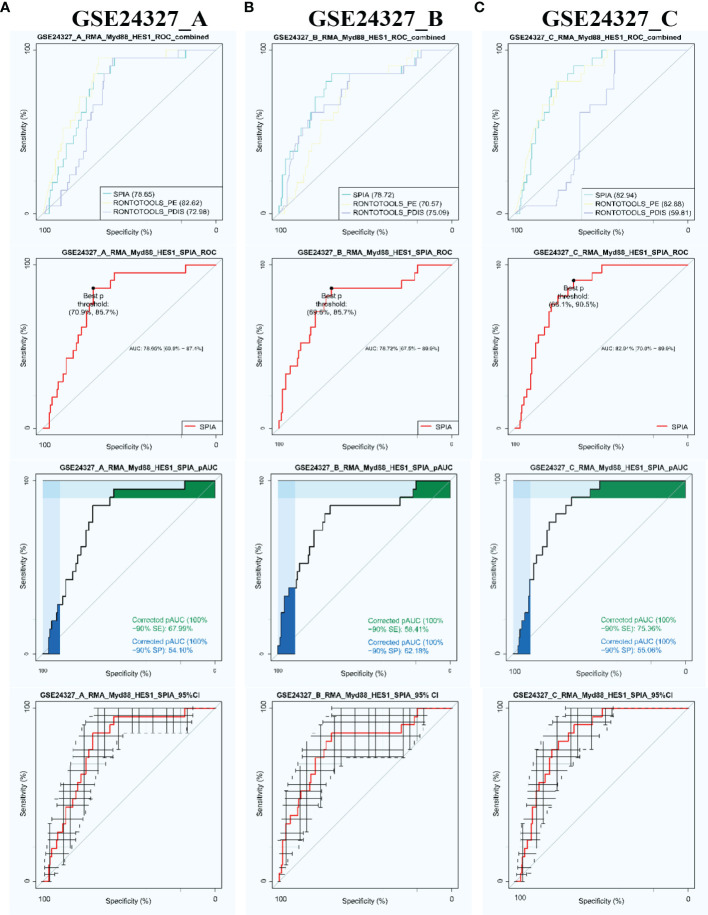
Automatic identification of target signaling pathways by using the PathwayKO package. The SPIA method demonstrates advantageous performance in view of the ROC-curve-based statistics metrics on each data. **(A)** GSE24327_A; **(B)** GSE24327_B; **(C)** GSE24327_C.

Firstly, we compared three popular methods of pathway enrichment analysis *via* the PathwayKO package benchmarked on each data under study (**Figure 2**). The SPIA method (43) has an overall better performance over ROntoToosls_PE (41) and ROntoTools_PDIS (42) in terms of the ROC-curve-based statistics metrics, such as AUC (with 95% CI), pAUC_SP, pAUC_SE, and Youden’s best *p*-threshold (**Figure 2**). The SPIA method is thus chosen to conduct subsequent analyses throughout this study.

Secondly, we used the SPIA method *via* the PathwayKO package to identify target signaling pathways with each HES1 threshold from each data (see **Figure 1**). As a result, we automatically identified the target 21 KO MyD88-associated signaling pathways from each data (**Table 1**). All pathways are identified as significant (*pGFdr <*0.0001) from the (**C**) treatment (septic wildtype *vs*. untreated wildtype). Only 1 pathway is identified as insignificant (*pGFdr >*0.05) from the (**A**) treatment (septic KO MyD88 mice *vs*. septic wildtype mice). Only 2 pathways are identified as insignificant (*pGFdr >*0.05) from the (**B**) treatment (septic KO MyD88 mice *vs*. untreated wildtype mice). These findings suggest that such insignificant pathways are likely dependent on MyD88-deficiency, i.e., the KO MyD88 deficiency has attenuated signaling. For instance, the number of true DEGs on the Toll-like signaling pathway is remarkably decreased from 52 in (**C**) treatment to 50 in (**B**) treatment, and to 34 in (**A**) treatment (**Table 1**).

**Table 1:** Target MyD88-associated signaling pathways identified by SPIA.

ID	Pathway Name	Status	pSize	DEGs (%)	pGFdr
***Signaling pathways from GSE24327_A (a. septic KO MyD88 vs. septic WT)***					
5140	Leishmaniasis	Activated	69	43 (62.3)	1.42E-32
5152	Tuberculosis	Activated	175	65 (37.1)	2.58E-32
5142	Chagas disease (American trypanosomiasis)	Activated	101	45 (44.6)	1.23E-25
5145	Toxoplasmosis	Activated	107	42 (39.3)	1.75E-22
5133	Pertussis	Activated	75	33 (44.0)	1.98E-18
5161	Hepatitis B	Activated	159	46 (28.9)	1.27E-17
**4620**	**Toll-like receptor signaling pathway**	**Inhibited**	**97**	**34 (35.1)**	**7.67E-16**
5170	Human immunodeficiency virus 1 infection	Activated	225	51 (22.7)	5.92E-15
**4010**	**MAPK signaling pathway**	**Inhibited**	**288**	**58 (20.1)**	**1.02E-14**
**5235**	**PD-L1 expression and PD-1 checkpoint pathway in cancer**	**Inhibited**	**88**	**29 (33.0)**	**1.32E-12**
5162	Measles	Activated	141	35 (24.8)	1.50E-11
**4621**	**NOD-like receptor signaling pathway**	**Activated**	**173**	**37 (21.4)**	**7.31E-10**
5135	Yersinia infection	Activated	120	29 (24.2)	4.50E-09
5169	Epstein-Barr virus infection	Activated	214	39 (18.2)	1.42E-08
5144	Malaria	Inhibited	51	18 (35.3)	1.49E-08
**4064**	**NF-kappa B signaling pathway**	**Inhibited**	**96**	**22 (22.9)**	**1.02E-07**
5164	Influenza A	Activated	162	30 (18.5)	8.01E-07
5134	Legionellosis	Inhibited	58	16 (27.6)	8.97E-07
5132	Salmonella infection	Inhibited	205	29 (14.1)	0.000233
5143	African trypanosomiasis	Inhibited	34	10 (29.4)	0.000556
5168	Herpes simplex virus 1 infection	Inhibited	391	31 (7.9)	0.254935
***Signaling pathways from GSE24327_B (b. septic KO MyD88 vs. untreated WT)***					
5169	Epstein-Barr virus infection	Inhibited	214	101 (47.2)	2.50E-85
5161	Hepatitis B	Inhibited	159	79 (49.7)	3.13E-67
5170	Human immunodeficiency virus 1 infection	Inhibited	225	83 (36.9)	2.29E-58
5162	Measles	Activated	141	69 (48.9)	1.04E-57
5168	Herpes simplex virus 1 infection	Activated	391	101 (25.8)	1.02E-56
5164	Influenza A	Activated	162	62 (38.3)	2.58E-43
**4620**	**Toll-like receptor signaling pathway**	**Inhibited**	**97**	**50 (51.5)**	**1.63E-42**
**4621**	**NOD-like receptor signaling pathway**	**Activated**	**173**	**56 (32.4)**	**1.49E-34**
5152	Tuberculosis	Activated	175	45 (25.7)	6.24E-23
5145	Toxoplasmosis	Activated	107	35 (32.7)	1.87E-21
5142	Chagas disease (American trypanosomiasis)	Activated	101	34 (33.7)	5.32E-21
5135	Yersinia infection	Inhibited	120	32 (26.7)	2.17E-16
**5235**	**PD-L1 expression and PD-1 checkpoint pathway in cancer**	**Activated**	**88**	**27 (30.7)**	**1.38E-15**
5133	Pertussis	Activated	75	24 (32.0)	4.56E-14
5140	Leishmaniasis	Activated	69	22 (31.9)	1.46E-13
5132	Salmonella infection	Activated	205	33 (16.1)	2.14E-10
**4010**	**MAPK signaling pathway**	**Inhibited**	**288**	**39 (13.5)**	**5.97E-10**
**4064**	**NF-kappa B signaling pathway**	**Activated**	**96**	**18 (18.8)**	**2.88E-08**
5134	Legionellosis	Inhibited	58	7 (12.1)	0.038191
5143	African trypanosomiasis	Activated	34	4 (11.8)	0.134315
5144	Malaria	Inhibited	51	5 (9.8)	0.245104
***Signaling pathways from GSE24327_C (c. septic WT vs. untreated WT)***					
5169	Epstein-Barr virus infection	Activated	214	105 (49.1)	2.44E-48
5170	Human immunodeficiency virus 1 infection	Inhibited	225	104 (46.2)	8.87E-45
5161	Hepatitis B	Activated	159	84 (52.8)	2.17E-41
5162	Measles	Activated	141	75 (53.2)	5.12E-37
5152	Tuberculosis	Inhibited	175	80 (45.7)	1.99E-33
5145	Toxoplasmosis	Inhibited	107	56 (52.3)	4.51E-27
5142	Chagas disease (American trypanosomiasis)	Activated	101	54 (53.5)	2.09E-26
**4620**	**Toll-like receptor signaling pathway**	**Activated**	**97**	**52 (53.6)**	**9.15E-26**
5133	Pertussis	Activated	75	46 (61.3)	9.15E-26
5140	Leishmaniasis	Activated	69	40 (58.0)	1.39E-21
5164	Influenza A	Activated	162	62 (38.3)	5.06E-21
**4621**	**NOD-like receptor signaling pathway**	**Activated**	**173**	**63 (36.4)**	**6.57E-20**
**4010**	**MAPK signaling pathway**	**Activated**	**288**	**80 (27.8)**	**8.56E-18**
5135	Yersinia infection	Inhibited	120	46 (38.3)	2.78E-15
5168	Herpes simplex virus 1 infection	Activated	391	88 (22.5)	7.05E-15
**5235**	**PD-L1 expression and PD-1 checkpoint pathway in cancer**	**Activated**	**88**	**37 (42.1)**	**2.82E-14**
**4064**	**NF-kappa B signaling pathway**	**Activated**	**96**	**34 (35.4)**	**5.39E-13**
5132	Salmonella infection	Activated	205	52 (25.4)	3.99E-10
5144	Malaria	Activated	51	23 (45.1)	1.47E-09
5134	Legionellosis	Activated	58	22 (37.9)	5.57E-08
5143	African trypanosomiasis	Inhibited	34	15 (44.1)	3.25E-06

Bold indicates the signaling pathways discussed in the main text. The rest pathways are provided online in **Supplementary Material**. pGFdr < 0.001***, < 0.01**, < 0.05*.

### Comparison on the Target Signaling Pathways Among Experimental Treatments

Each of the above 21 target signaling pathways (**Table 1**) contains MyD88, which is impaired by KO MyD88 in deficient mice. We named the Toll-like receptor signaling pathway (mmu04620) as the basal target pathway that is solely responsible for the KO MyD88 phenotype (**Figure 3**). We also named other target pathways as composite target pathways because they embed the named basal signaling pathway (mmu04620) coupled with others. Some examples of common core pathways are highlighted (**Figures 3**–**7**), while the rest of infection-related pathways are provided online (**Supplementary Figures S1–S16**). Both are in the context of comparing innate immune responses, triggered 12 hours after the CASP-surgery, among the experimental treatments born by the three subtypes of data under study.

**Figure 3:**
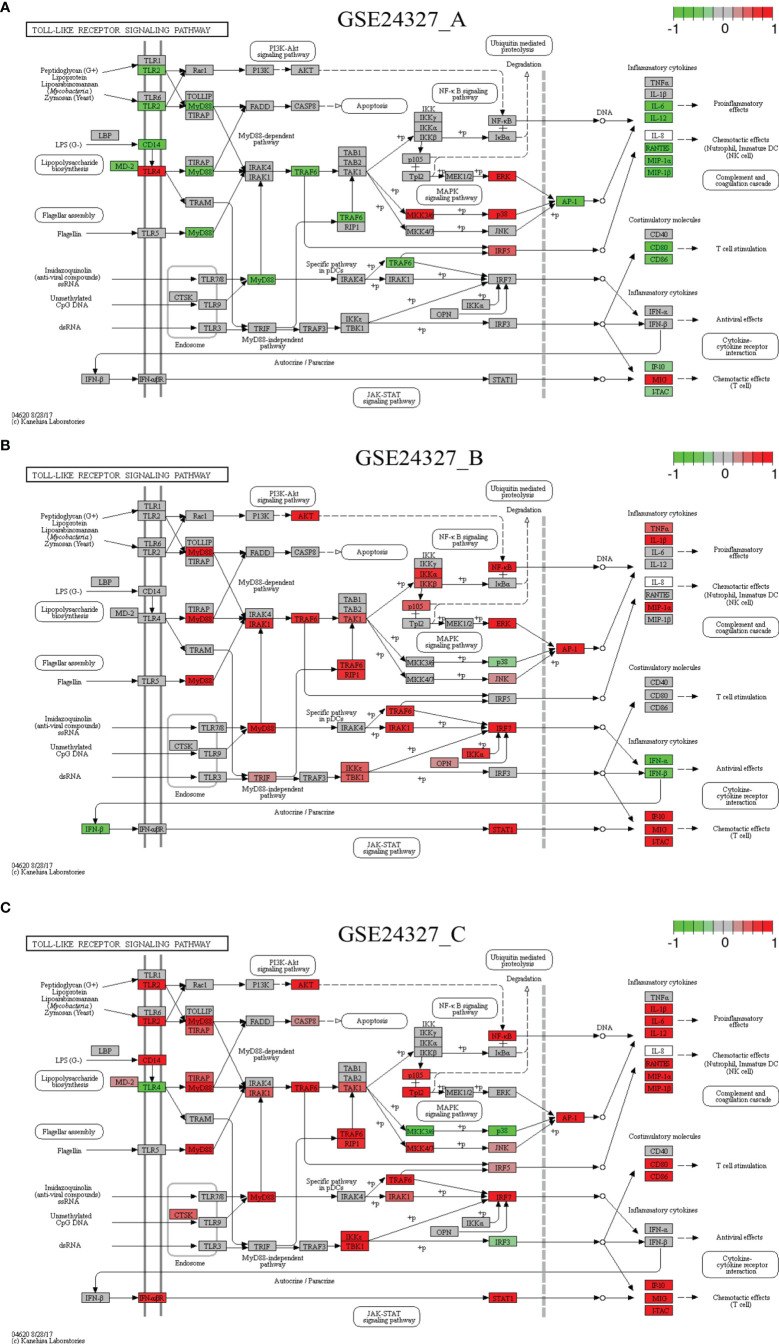
Comparison on the rendered Toll-like receptor signaling pathway (mmu04620). **(A)** GSE24327_A; **(B)** GSE24327_B; **(C)** GSE24327_C.

**Figure 4:**
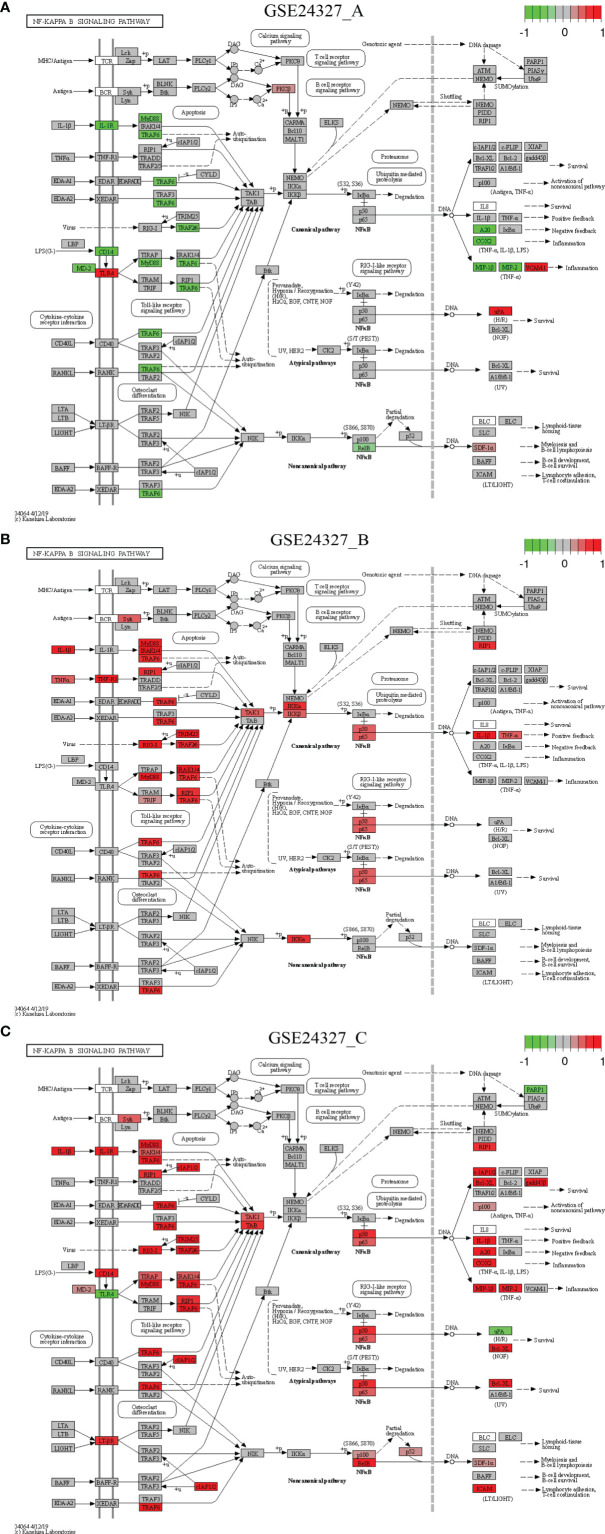
Comparison on the rendered NF-κB signaling pathway (mmu04064). **(A)** GSE24327_A; **(B)** GSE24327_B; **(C)** GSE24327_C.

**Figure 5:**
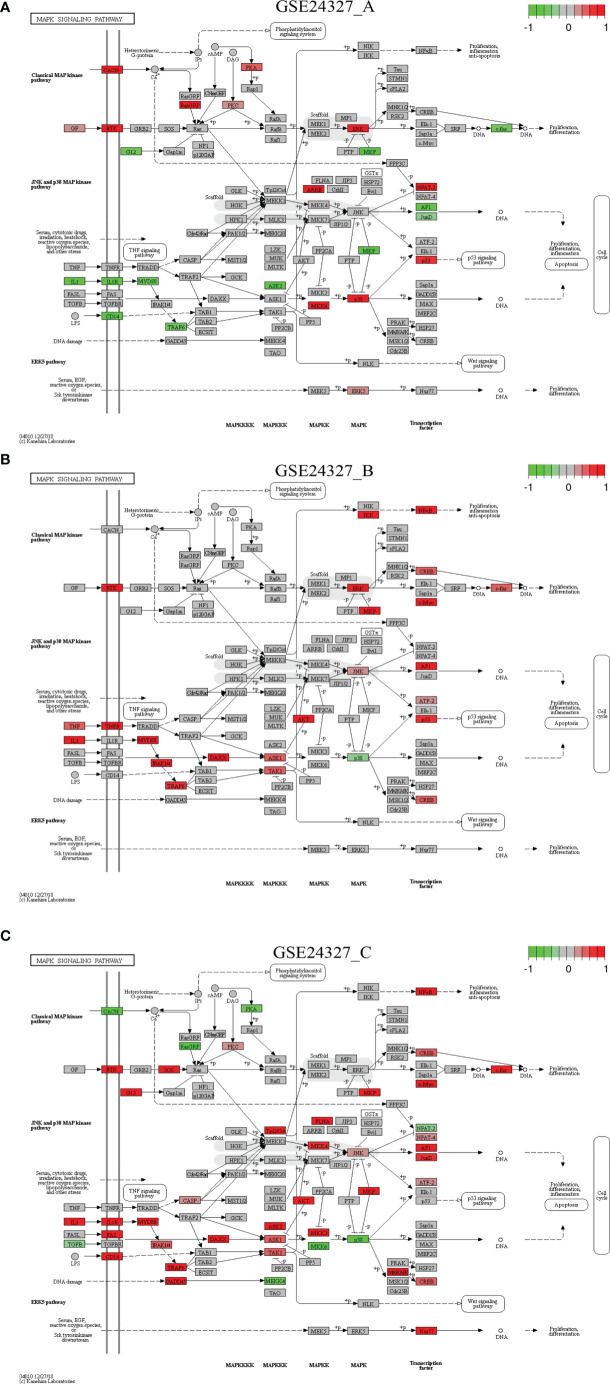
Comparison on the rendered MAPK signaling pathway (mmu04010). **(A)** GSE24327_A; **(B)** GSE24327_B; **(C)** GSE24327_C.

**Figure 6:**
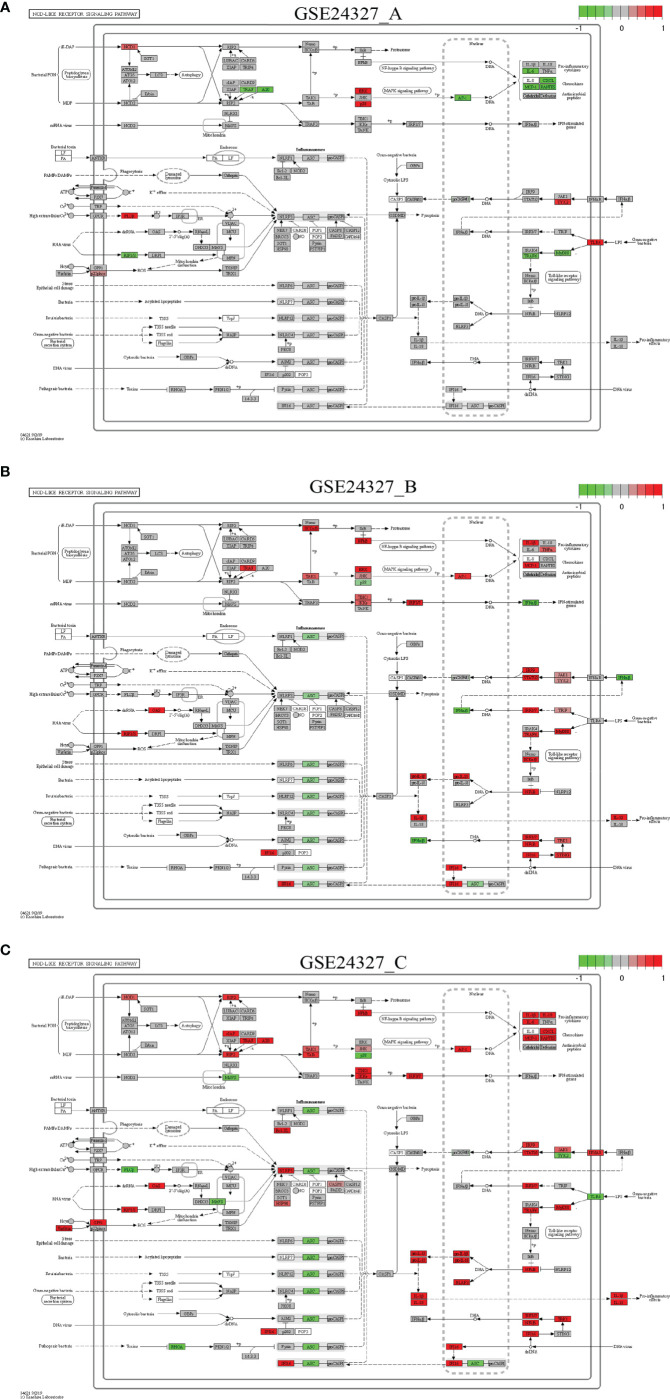
Comparison on the rendered NOD-like receptor signaling pathway (mmu04621). **(A)** GSE24327_A; **(B)** GSE24327_B; **(C)** GSE24327_C.

**Figure 7:**
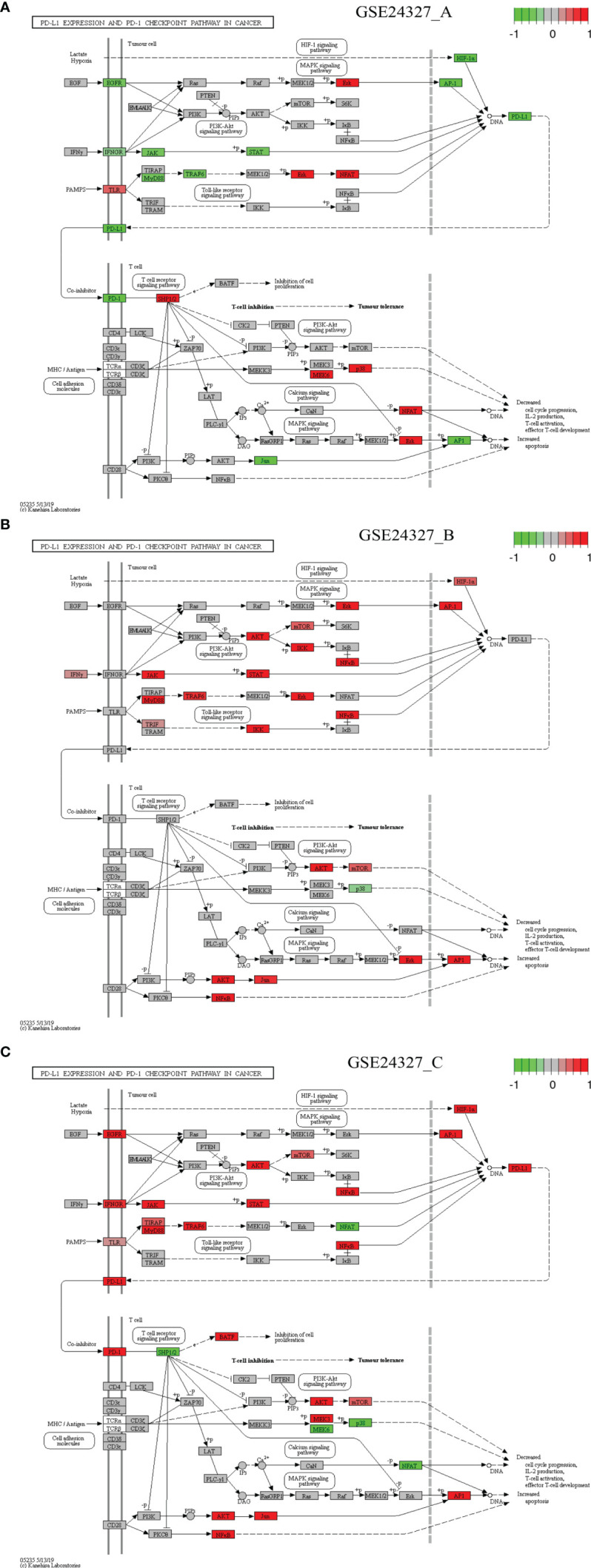
Comparison on the rendered PD-L1 expression and PD-1 checkpoint pathway in cancer (mmu05235). **(A)** GSE24327_A; **(B)** GSE24327_B; **(C)** GSE24327_C.

There are common features of comparisons on the target signaling pathways: **1**) the (**C**) treatment provokes the strongest innate immune responses at the systems-level at 12 hours after the CASP-surgery, **2**) the (**A**) or (**B**) treatment causes relatively weaker innate immune responses at the systems-level at 12 hours after the CASP-surgery, and **3**) some regulators on the target signaling pathways are dependent on the KO MyD88-deficiency, but others are independent of it.

*Case 1*: The Toll-like receptor signaling pathway (mmu04620) is the basal target pathway that is solely responsible for the KO MyD88 phenotype (**Figure 3**). It is a significant pathway (*pGFdr <*0.0001) identified from all three data (see **Table 1**). This pathway comprises 97 critical genes, ofs which 34 in (**A**), 50 in (**B**) and 52 in (**C**) treatment (see **Table 1**) are true DEGs that are coordinately, significantly, differentially up- or down-regulated at 12 hours after the CASP-surgery.

Firstly, in the (**C**) treatment, MyD88 is up-regulated, where the majority of ligands, receptors, adaptors and transducers (MD-2, TLR1, TLR2, CD14, INFαβR, CTSK, TIRAP, AKT, CASP8, IRAK1, TRAF6, TAK1, RIP1, IKK3/ϵ, TBK1, IRF7, IRF5, JNK, MKK4/7, Tp12, p105 and NFκB) are up-regulated. Only a few (TLR4, IRF3, MKK3/6 and p38) are down-regulated. Therefore, the downstream effectors [AP-1 (Jun, c-Jun), IL-1β, IL-6, IL12, RANTES (CCL5), MIP-1α (CCL3), MIP-1β (CCL4), CD80, CD86, IP-10 (CXCL10), MIG (CXCL9) and ITAC] are collectively up-regulated at the systems-level.

Secondly, for instance, in the (**A**) treatment, the majority of regulators are down-regulated, except for the few (TLR4, IKK3/ϵ, ERK, p38, IRF5 and MIG) that remain up-regulated. In particular, members of the major axis (MyD88, MD-2, CD14, TLR2, TRAF6 and AP-1) are drastically down-regulated and the downstream effectors (IL-6, IL-12, RANTES, MIP-1α, MIP-1β, CD80, CD86, IP-10 and ITAC) are drastically attenuated at the systems-level. These data suggest that the KO MyD88-deficiency had attenuated the expression level of major inflammatory effectors (IL-6, IL-12, RANTES, MIP-1α, MIP-1β, CD80, CD86, IP-10 and ITAC) at the systems-level in deficient mice.

*Case 2*: The NF-κB signaling pathway (mmu04064) is a composite target pathway, which embeds the Toll-like signaling pathway, B-cell receptor signaling pathway, T-cell receptor signaling pathway, and RIG-I-like receptor signaling pathway (**Figure 4**). It is a significant (*pGFdr <*0.0001) target pathway identified from all three data (**Table 1**). Among 96 critical genes, 22 in (**A**), 18 in (**B**) and 34 in (**C**) treatment (see **Table 1**) are true DEGs that are coordinately, significantly, differentially up- or down-regulated at 12 hours after the CASP-surgery.

Firstly, in the (**C**) treatment, MyD88 is up-regulated, of which the majority of ligands, receptors, adaptors and transducers (IL-1β, IL-1R, MD-2, CD14, LT-βR, MyD88, TRAF6, Syk, IRAK1/4, RIP1, RIG-1, TIRAP, cIAP1/2, TRIM25, TRAF2/6, TAK1, TAB, p50, p52, p65, p100, RelB, RIP1, Bcl-XL, gadd45β, IL-1β, A20, COX2, MIP-1β (CCL4), MIP-2 (CXCL2), SDF-1α (CXCL12) and ICAM (CD54)) are up-regulated. Only a few (TLR4, uPA and PARP-1) are down-regulated. These key regulators are related to inflammation, survival, myeloiesis, B-cell lymphopoiesis, lymphocyte adhesion and T-cell co-stimulation at the systems-level.

Secondly, in the (**A**) treatment, the majority of regulators [MD-2, CD14, IL-1R, MyD88, TRAF6, RelB, A20, COX2, MIP-1β (CCL4) and MIP-2 (CXCL2)] are down-regulated except for a few [TLR4, PKCβ, uPA, VCNM1 and SDF-1α (CXCL12)] that are up-regulated. These regulators are related to inflammation, survival, myeloiesis and B-cell lymphopoiesis at the systems-level.

*Case 3*: The MAPK signaling pathway (mmu04010) is a composite target pathway, which embeds the Toll-like signaling pathway, TNF signaling pathway, and p53 signaling pathway (**Figure 5**). It is identified as a significant (*pGFdr <*0.0001) target pathway from all three data (**Table 1**). Among 288 critical genes, 58 in (**A**), 39 in (**B**) and 80 in (**C**) treatment (see **Table 1**) are true DEGs that are coordinately, significantly, differentially up- or down-regulated at 12 hours after the CASP-surgery. The classic MAPK pathway differs from the JNK/p38 MAPK pathway because the latter further incorporates the Toll-like signaling pathway, TNF signaling pathway, p53 signaling pathway and ERK5 signaling pathway.

Firstly, in the (**C**) treatment: 1) on the classic MAPK pathway, regulators (RTK, G12, SOS, PKC, MKP, NFκB, CREB, c-Myc and c-Fos) are up-regulated, while a few (CACN, RasGRP and PKA) are down-regulated, and 2) on the JNK/p38 MAPK pathway, the majority of regulators (IL1, IL1R, CD14, MyD88, FAS, IRAK1/4, TRAF6, GADD45, CASP, DAXX, TAK1, ASK1, ASK2, Tpl2/Co, AKT, MKK3, MKK4, FLNA, JNK, MKP, MAFKAFK, CREB, NFAT4, AP1, JunD, ATF-2 and Nur77) are up-regulated, while only a few (TGFB, MEKK4, MKK6, p38, and NFAT2) are down-regulated. These key regulators positively contribute to cell cycle regulation, such as proliferation, differentiation, inflammation, and anti-apoptosis at the systems-level.

Secondly, for instance, in the (**A**) treatment: 1) on the classic MAPK pathway, regulators (GF, RTK, CACN, RasGRP, PKA, PKC, and ERK) are up-regulated, while a few (G12, MKP and c-Fos) are down-regulated, and 2) on the JNK/p38 MAPK pathway, regulators (ARRB, MKK6, p38, p53, NFAT2, and ERK5) are up-regulated, while a few (IL1, IL1R, CD14, MyD88, TRAF6, ASK2, MKP, AP1, and JunD) are down-regulated at the systems-level.

*Case 4*: The NOD-like receptor signaling pathway (mmu04621) is a composite target pathway, which embeds the Toll-like signaling pathway, NFκB signaling pathway and MAPK signaling pathway (**Figure 6**). It is identified as a significant (*pGFdr <*0.0001) target pathway from all three data (**Table 1**). Among 173 critical genes, 37 in (**A**), 56 in (**B**), and 63 in (**C**) treatment (see **Table 1**) are true DEGs that are coordinately, significantly, differentially up- or down-regulated at 12 hours after the CASP-surgery.

Firstly, in the **(C)** treatment, MyD88 is up-regulated, around which the majority of regulators (IRF3/7, TRAF6, NFκB, JAK1, IFNAR, IRF9, STAT1/2, TBK1, IF116, NIRP3, AP-1, JNK, TAK1, TAB, IKKϵ, RIP2, cIAP, TRAF, A20, RIF2, NOD1, Bcl-XL, NLRP3, CASP8, HSP90, RIP1/3, OAS, GP91 and Visfatin) are up-regulated. Only a few regulators (TLR4, TYK2, ASC, MAYS, PLCβ, MAVS, RHOA and p38) are down-regulated. The downstream effectors [IL-1β, IL-18, pro-IL-1β, pro-IL-18, IL-6, CXCL, MCP-1 (CCL2) and RANTES (CCL5)] are accordingly up-regulated. Note that ASC means antibody secreting cells (18).

Secondly, in the **(A)** treatment, only a few regulators (MyD88, TRAF6, TRAF, A20, AP-1 and RIP1/3) are down-regulated, while a few (TLR4, TYK2, NOD1, ERK, p38, PLCβ and p22phox) are up-regulated. The downstream effectors (IL-6, CXCL, MCP-1 and RANTES) are accordingly down-regulated at the systems-level.

*Case 5*: The PD-L1 expression and PD-1 checkpoint pathway in cancer (mmu05235) is a composite target signaling pathway, which embeds the Toll-like signaling pathway, MAPK signaling pathway, PI3K-Akt signaling pathway, HIF-1 signaling pathway, and T-cell receptor signaling pathway (**Figure 7**). It is a significant (*pGFdr <*0.0001) target pathway identified from all three data (**Table 1**). Among 88 critical genes, 29 in (**A**), 27 in (**B**), and 37 in (**C**) treatment (see **Table 1**) are true DEGs that are coordinately, significantly, differentially up- or down-regulated at 12 hours after the CASP-surgery.

Firstly, in the (**C**) treatment: 1) on the Toll-like receptor signaling pathway, a few regulators (MyD88, TLR, TIRAP, TRAF6 and NFκB) are up-regulated, while only NFAT is down-regulated; 2) on the HIF-1 signaling pathway, HIF-1α is up-regulated; 3) on the MAPK signaling pathway, only EGFR and AP-1 are up-regulated; 4) on the PI3K-AKT signaling pathway, a few regulators (IFNGR, JAK, STAT, AKT, mTOR and NFκB) are up-regulated. All of these key regulators positively contribute to the increased production of PD-L1; and 5) on the T-cell receptor signaling pathway, the major regulators (PD-L1, PD-1, BATF, AKT, mTOR, NFκB, MEK3, JUN and AP-1) are up-regulated, while a few (SHP1/2, MEK6, p38 and NFAT) are down-regulated. All of these key regulators positively contribute to the increased apoptosis but decreased cell cycle progression, IL-2 production, T-cell activation, and effector T-cell development at the systems-level.

Secondly, in the (**A**) treatment: 1) on the Toll-like receptor signaling pathway, a few regulators (TLR, ERK and NFAT) are up-regulated, while a few (MyD88 and TRAF6) are down-regulated; 2) on the HIF-1 signaling pathway, only HIF-1α is down-regulated; 3) on the MAPK signaling pathway, only EGFR and AP-1 are down-regulated, while only ERK is up-regulated; and 4) on the PI3K-AKT signaling pathway, a few regulators (IFNGR, JAK and STAT) are down-regulated. All of these key regulators contribute to the decreased production of PD-L1 and 5) on the T-cell receptor signaling pathway, a few regulators (PD-L1, PD-1, JUN and AP-1) are down-regulated, while a few (SHP1/2, MEK6, p38 and NFAT) are up-regulated. All of these key regulators inversely contribute to the increased apoptosis and decreased cell cycle progression, IL-2 production, T-cell activation, and effector T-cell development at the systems-level.

### The Infection-Related Composite Signaling Pathways Responsible for Systemic Syndromes

Each infection-related signaling pathway embeds the basal Toll-like receptor signaling pathway coupled with more than one of the other composite signaling pathways (**Supplementary Figures S1–S16**). 1) The viral infection-related pathways include Epstein-Barr virus (**Supplementary Figure S1**), Human immunodeficiency virus 1 (**Supplementary Figure S2**), Hepatitis B (**Supplementary Figure S3**), Measles (**Supplementary Figure S4**), Influenza A (**Supplementary Figure S5**) and Herpes simplex virus 1 (**Supplementary Figure S6**); 2) The bacterial infection-related pathways include **Tuberculosis** (**Supplementary Figure S7**), Pertussis (**Supplementary Figure S8**), Yersinia (**Supplementary Figure S9**), Salmonella (**Supplementary Figure S10**) and Legionellosis (**Supplementary Figure S11**): and 3) The parasitic infection-related pathways include Toxoplasmosis (**Supplementary Figure S12**), Chagas disease (**Supplementary Figure S13**), Leishmaniasis (**Supplementary Figure S14**), Malaria (**Supplementary Figure S15**) and African trypanosomiasis (**Supplementary Figure S16**). The majority of these pathways are identified as significant (*pGFdr <*0.0001 or 0.05) from the (**A**) and (**B**) treatment, respectively; only one pathway (Herpes simplex virus 1) and two pathways (Malaria and African trypanosomiasis) are identified as insignificant (*pGFdr >*0.05) from the (**A**) and (**B**) treatment, respectively. All of these pathways are identified as significant (*pGFdr <*0.0001) from the (**C**) treatment that compared septic wildtype with untreated wildtype mice. These data suggest possible death from systemic syndromes, which increase the difficulty of early diagnosis and prevention of sepsis because it is unlikely that such systemic syndromes can be precisely attributed to a specific type (viral, bacterial or parasitic) of infections.

### The Top-Ranked Intersected DEGs Responsible for Innate Immune Responses

To focus on the roles of individual key regulators among the thousands of true DEGs (**Supplementary Tables S1–S3**), we further conducted the Venn diagram analysis to extract top 50-ranked DEGs intersected across GSE24327_A, B and C data (**Table 2**). As a result, there are 82 distinct core DEGs responsible for the immune-cell growth, activation and function (**Table 2**), including 1) ligands, receptors and adaptors (MyD88 and Lepr); 2) signal transducers (Jak1~Jak3, Stat3, Mapk1~Mapk3, Mapk8~Mapk14, Rela, Jun, Fos, Mmp9, Osmr and Pdgfra); 3) transcriptional factors (Nfkb1, Hif1a, Bcl3 and Eif3e); and 4) downstream effectors, such as colony-stimulating factors (Csf3r and Csf2rb), interleukins (Il1a, Il2ra, Il6ra and Il15ra), and chemokines (Ccr1~Ccr10, Cxcr1~Cxcr6, Cx3cr1, Ccr1l1 and Xcr1).

**Table 2:** Top 50-ranked DEGs intersected across GSE24327_A, B and C data.

#	A only	B only	C only	A and B and C	A and B	A and C	B and C
1	Sdc1	Oas1b	Rnf125	**Ccr3**	Ccr3	Cxcl2	Irf7
2	Timp3	Bst2	Bcl2l11	**Cxcr2**	Cxcr2	Ccr3	Cxcl10
3	Ncf1	**Ifna6**	Mmp14	**Ccr2**	Ccr2	Cxcr2	Zbp1
4	Ncf4	**Ifna12**	Col4a1	**Ccl3**	Ccl3	Cxcl1	Eif3e
5	Cd8a	Chuk	Irs3	**Xcr1**	Xcr1	Ccr2	Eif2ak2
6	Gpr83	**Ifnb1**	Irs1	**Ccr7**	Ccr7	Ccl3	Ifit1
7	Pdgfc	**Ifna15**	Srebf1	Hif1a	Hif1a	Cxcl3	Stat2
8	Cacna2d1	**Ifnab**	Gsk3b	**Ccr1l1**	Ccr1l1	Cxcl5	Mx2
9	Cr2	**Tnf**	Itga5	**Cx3cr1**	Cx3cr1	Ccl4	Oas2
10	Klk1	**Ifnk**	Ddit4	**Ccr1**	Ccr1	Xcr1	Il1b
11	Flt3l	**Ifna9**	Col4a2	**Ccr6**	Ccr6	Ccr7	Il1r2
12	Rasgrp4	**Ifne**	Nr4a1	**Csf3r**	Eif3i	Hif1a	Oas3
13	Ncf2	**Ifna13**	Star	**Ccr8**	Eif2s3y	Ccr1l1	Il1rn
14	Dynll1	Ticam1	Fas	**Cxcr6**	Csf3r	Cx3cr1	Ccr1
15	H2-DMb2	**Ifna5**	Slc3a2	Jun	Ccr8	Ccr1	Oasl1
16	Ngf	**Ifna4**	Slc7a11	**Cxcr3**	Cxcr6	Ccr6	Oasl2
17	Parvg	**Ifng**	Plxnb3	Eif3e	Jun	Nfatc1	H2-T24
18	Cyba	**Il15**	Vdr	**Ccr9**	Eif2b1	Il6	Cdkn1a
19	2210010C04Rik	Irak4	Ripk2	Fos	Eif2b5	Csf3r	Ccl12
20	F2rl3	**Ifna2**	Rhod	Mapk3	Eif3g	Ccr8	Stat1
21	Clec7a	Spp1	Runx2	Myd88	Cxcr3	Il9r	Cxcr2
22	H2-Ob	**Ifna11**	Mmp13	**Ccr4**	Eif3e	Cxcr6	Irf9
23	Src	**Ifna14**	Plxna4	Mapk14	Eif3b	Ccl11	Clec4e
24	Vcam1	Ikbkg	Nos3	**Ccr5**	Eif2b3	Nfatc2	Clec4d
25	H2-Oa	**Tnfrsf1a**	Sema4c	**Csf2rb**	Ccr9	Jun	Socs3
26	Ngfr	Trim30d	Dffa	Jak3	Eif3d	Lifr	Il1a
27	Irak3	**Ifi207**	Mapkapk2	Mmp9	Eif2b2	Cxcr3	Bcl3
28	Chek1	Tmem173	Lmnb1	**Il6ra**	Eif3j2	Il27ra	Ccl22
29	Tcf7l2	Tapbp	Lilrb4a	Lepr	Eif3h	Eif3e	Dhx58
30	Prkcb	Socs7	Nlrp3	Jak2	Eif3f	Ccr9	Ifih1
31	Cd247	Ap1s2	Il18	Rela	Eif2b4	Cd209a	Eif2ak1
32	Fzd4	Nog	Cebpb	Mapk11	Eif3c	Il10	Rela
33	Pld2	Ppp2r1a	Lif	Nfkb1	Eif3a	Csf2rb2	Nfkb1
34	–	Nbl1	Parp1	**Cxcr4**	Fos	Fos	Map3k7
35	–	A430033K04Rik	Itga9	**Cxcr1**	Mapk3	Ccl2	Map3k5
36	–	Ap1g1	Pla1a	**Cxcr5**	Myd88	Mapk3	Ccr5
37	–	Ikbkb	Gnaq	**Ccr10**	Ccr4	Myd88	Cxcl9
38	–	Grem1	Itgb5	Stat3	Mapk14	Ccr4	Oas1a
39	–	Ppp2r2c	Lama5	**Il15ra**	Ccr5	Il20rb	Map3k3
40	–	Zfp936	Atp2b4	**Il1a**	Csf2rb	Il5ra	Eif2ak4
41	–	**Ifi206**	Tnxb	Mapk12	Jak3	Mapk14	Eif2ak3
42	–	Zfp108	Tuba3b	Mapk13	Mmp9	Csf2ra	Ddx58
43	–	Pias1	Casp8	Mapk1	Il6ra	Fosl1	Ccr6
44	–	Amhr2	Tuba3a	**Il2ra**	Lepr	Ccr5	Xcr1
45	–	Zfp874b	Tubal3	Bcl3	Jak2	Il2rb	Ep300
46	–	Socs6	Bid	Jak1	Rela	Il10rb	Irak1
47	–	–	Parp4	Mapk10	Mapk11	Ccl6	Traf6
48	–	–	Mmp16	Osmr	Nfkb1	Ccl5	Jun
49	–	–	Tuba4a	Pdgfra	Cxcr4	Csf2rb	Cxcr6
50	–	–	Casp7	Mapk8	Cxcr1	Jak3	Ikbke

Bold indicates massive cytokines (i.e., called “cytokine storm”). A= GSE24327_A (septic KO MyD88 vs. septic wildtype (WT)); B= GSE24327_B (septic KO MyD88 vs. untreated WT); C= GSE24327_C (septic WT vs. untreated WT).

Interestingly, the tumor necrosis factors (TNFs: Tnf and Tnfrsf1a) and the interferons (IFNs: Ifna2, Ifna4~Ifna6, Ifna9, Ifna11~Ifna15, Ifnab, Ifnb1, Ifne, Ifng, Ifnk, Ifi206, Ifi207) are categorized in the (**B**) treatment (septic KO MyD88 *vs*. untreated wildtype) (see Bonly in **Table 2**). In contrast, chemokines are intersected across all of the three data (see AandBandC in **Table 2**). It is well-known that TNFs stimulate immune-cell proliferation and activation; type I IFNs mediate antiviral immune responses, type II IFNs are responsible for antibacterial responses; and chemokines recruit different immune cells to the site of injury and/or infection. Taken together, our data suggest that the anti-viral (by Type I IFNs) and the anti-bacterial (by Type II IFNs) effects, as well as the recruitment (by chemokines) effects, have been strongly induced in spleen by the CASP-surgery on intestine just after 12 hours of the surgery, which strongly suggests a rapid progression of the CASP-model sepsis across remote organs in the body.

### Infiltration of Core Immune Cells Recruited by Key Regulators

**Figure 8:**
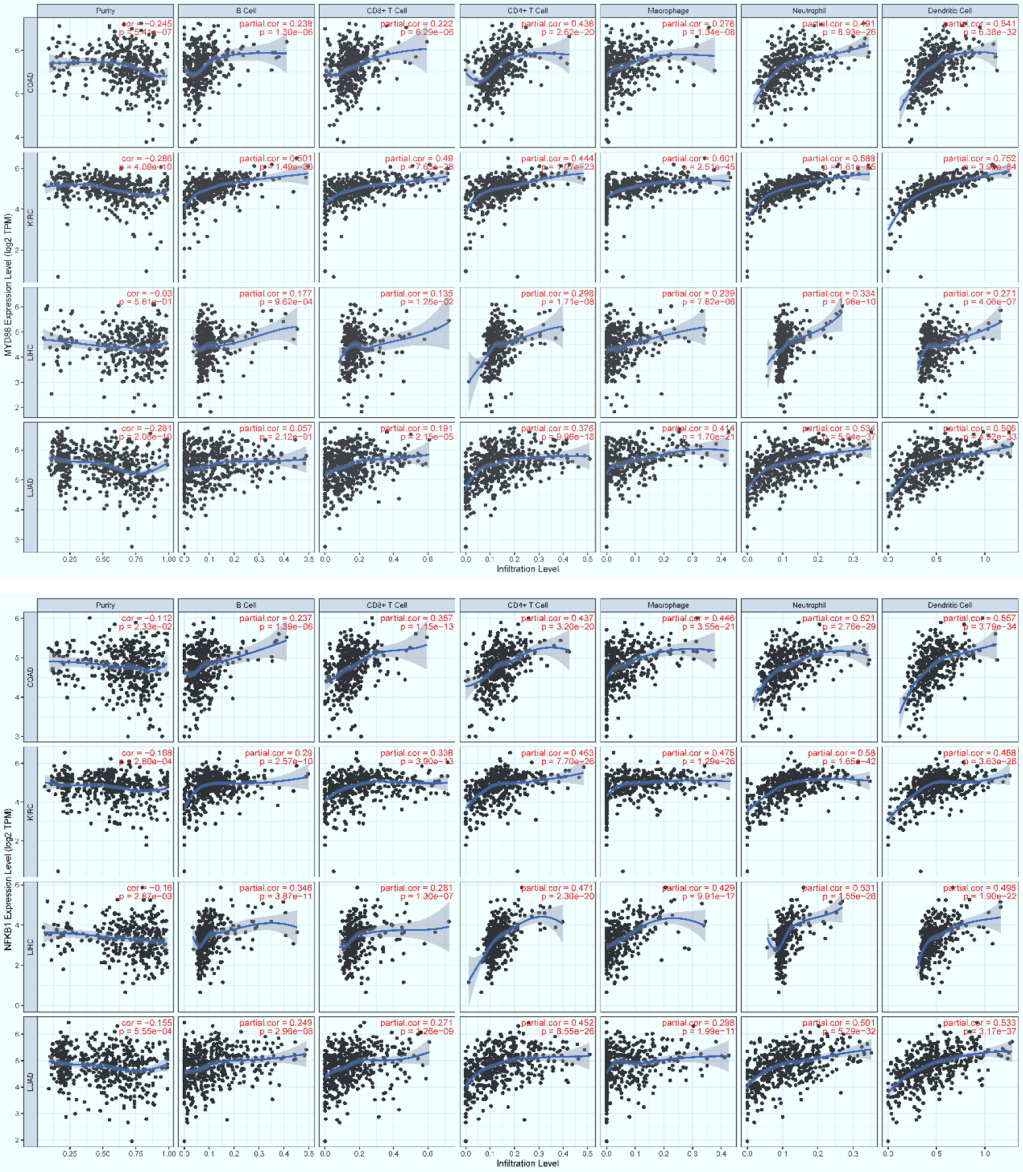
The positive, significant correlation between the infiltration level of core immune cells (X-axis) and the expression level of key regulators (Y-axis), MyD88 (top panel) and Nfkb1 (bottom panel). COAD: colon, KIRC: kidney, LIHC: liver, and LUAD: lung. *p* < 0.001***.

**Figure 9:**
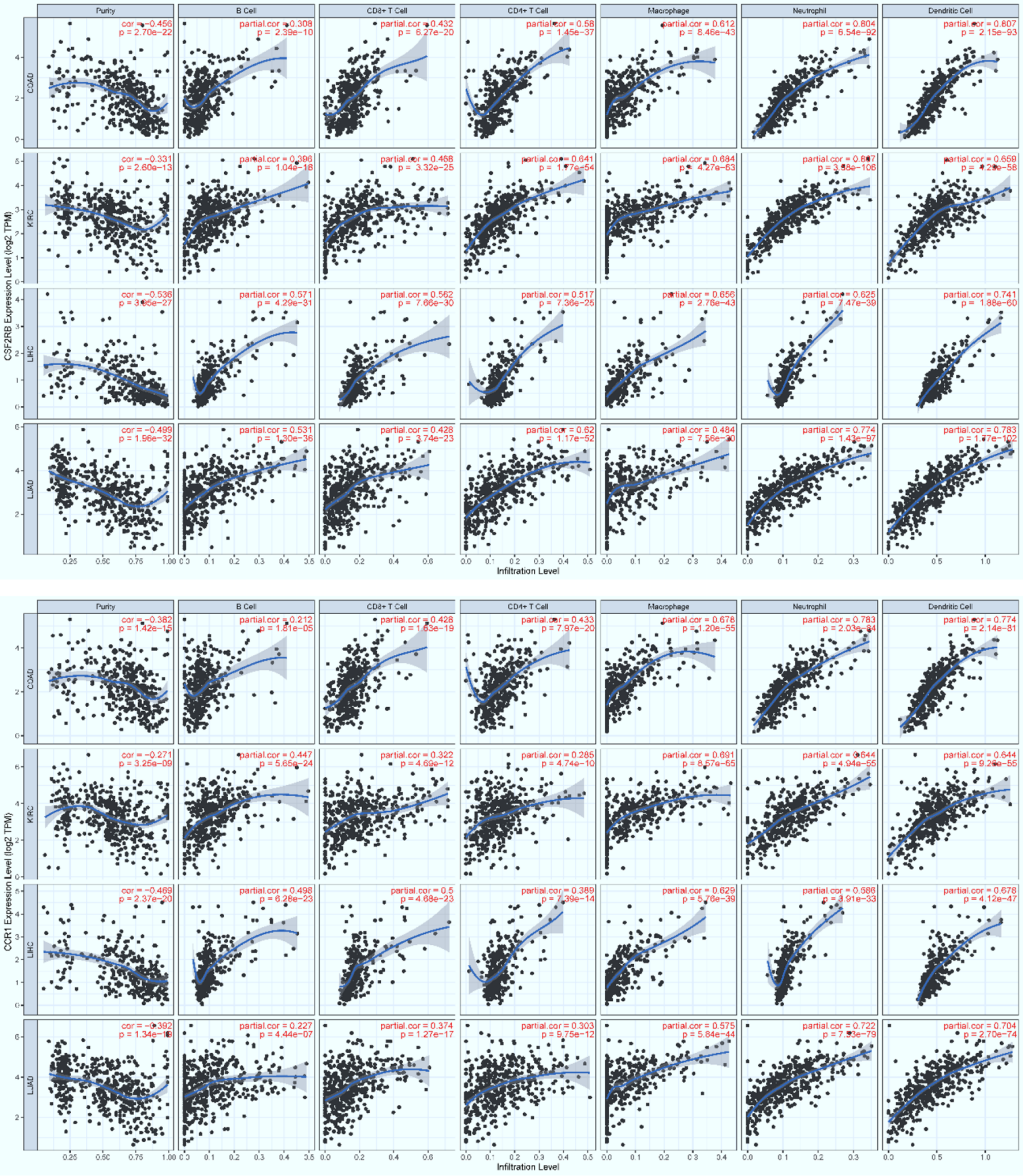
The positive, significant correlation between the infiltration level of core immune cells (X-axis) and the expression level of key regulators (Y-axis), Csf2rb (top panel) and Ccr1 (bottom panel). COAD: colon, KIRC: kidney, LIHC: liver, and LUAD: lung. *p* < 0.001***.

To examine possible infiltration of the core immune cells (CICs) (including B, CD4+T, CD8+T, Neutrophil, Macrophage and Dendritic cells) that would be recruited by the top 50-ranked regulators intersected across all of the three data (see **Table 2**), we searched them against multiple cancer cohorts, including colon (COAD), kidney (KIRC), liver (LIHC) and lung (LUAD) in database TIMER2.0 (51) at https://cistrome.shinyapps.io/timer. As a result, the expression level of each key regulator is positively and significantly (*p*<0.001) correlated with the infiltration level of CICs. Some examples are highlighted (**Figures 8**, **9**), and more are provided online (**Supplementary Figures S17–S20**). These data suggest that the high expression of such regulators would recruit such infiltrating CICs at the systems-level. Our results imply that post-operative failure of remote multi-organs (colon, kidney, liver and lung) are likely due to the infiltration of such core immune cells that are recruited by the massive key regulators (including chemokines) and triggered by the CASP-model sepsis at the systems-level in the body (possibly progressing *via* the axis of intestine (colon)–heart–spleen–liver–kidney–lung, etc.).

## Discussion

This study aimed to elucidate the systemic mechanisms underlying the standard CASP-model sepsis in the body through top-down data driven analysis on high-throughput functional genomics data. The success is attributed to the excellent GSE24327 GEO data with three experimental treatments (24): (a). septic KO MyD88 *vs*. septic WT (GSE24327_A); (b). septic KO MyD88 *vs*. untreated WT (GSE24327_B); and (c). septic WT *vs*. untreated WT (GSE24327_C). These data were generated from samples recovered from spleen at 12 hours after the CASP-surgery in mice, which were used for mRNAs profiling on microarray (24). From this, we have observed novel facts that have not been addressed thus far. Our data suggest that the leakage of intestinal contents effectively induced polymicrobial sepsis through provoking excessive innate immune responses at the systems-level (**Figures 3**–**7**; **Supplementary Figures S1–S16**) in wildtype mice, which usually caused post-operative failure of multi-organs (including colon, kidney, liver and lung). Moreover, the complex phenotypic syndromes, born by the identified and characterized 21 (KO MyD88-associated) target signaling pathways, contributed to the rapid progression of severe sepsis and even possible emerging death from complicated syndromes in post-operative mice, much beyond what has been perceived in literature (24). Our results update a significant advance in understanding the CASP-model sepsis mechanisms that have been explored for decades (4, 8, 24, 27, 31, 52–56).

**Figure 10:**
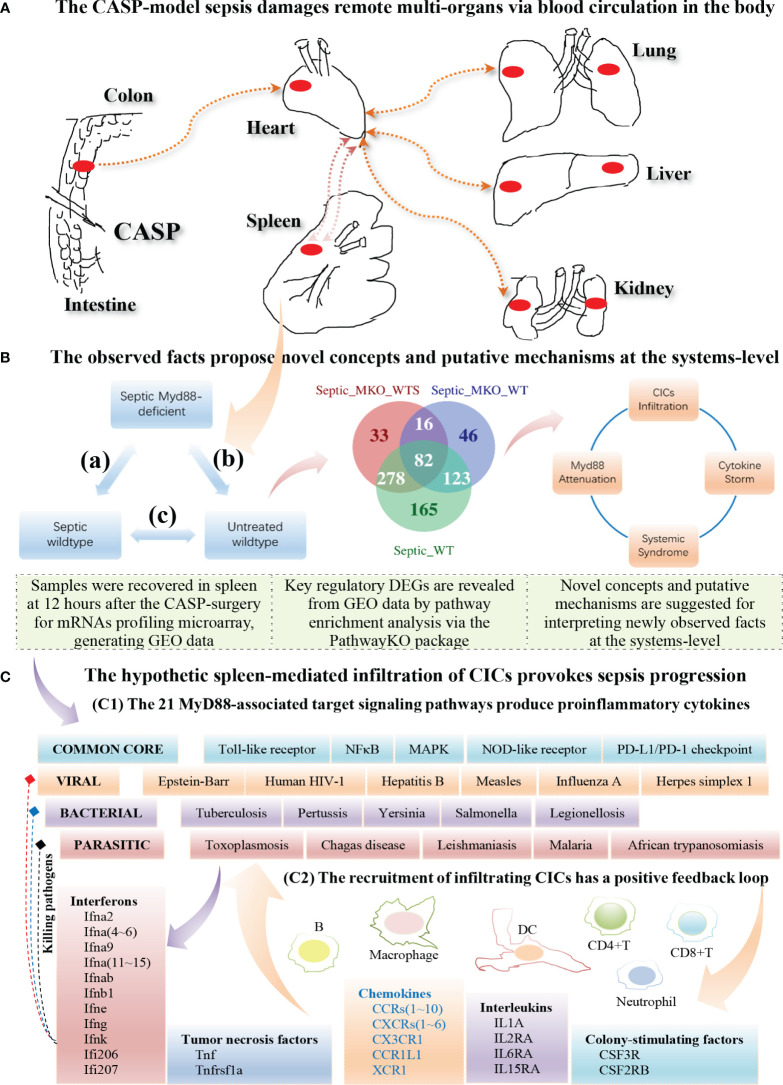
Hypothetic mechanisms underlying the innate immune responses at the systems-level triggered by the CASP-model sepsis at 12 hours after the CASP-surgery. **(A)** The CASP-model sepsis damages remote multi-organs through blood circulation in the body (top panel). **(B)** The observed facts propose novel concepts and putative mechanisms at the systems-level (middle panel). **(C)** The hypothetic spleen-mediated infiltration of core immune cells (CICs) (including B, CD8+T, CD4+T, Macrophage, Neutrophil and Dendritic cells) recruited by key cytokines (including CCR1, CSF2RB and IL2RA) likely drives the rapid progression of sepsis (bottom panel).

Based on the 21 KO MyD88-associated target signaling pathways, we conceptualize the systemic mechanisms underlying the innate immune responses triggered by the CASP-model sepsis (**Figure 10**). The core regulators intersected across all data (**Table 2**) are coordinately, significantly, and differentially expressed at the systems-level, in both wildtype and deficient mice, suggesting unknown mechanisms that have not been addressed thus far. Such core regulators (e.g., CCR1, CSF2RB and IL2RA) would recruit the infiltration of core immune cells (CICs) (including B, CD4+T, CD8+T, macrophage, neutrophil, and dendritic cells) of the immune system (**Figures 8**, **9**; **Supplementary Figures S17–S20**), likely initiated in spleen and transferred into remote multi-organs (progressing *via* the axis of colon–heart–spleen–liver–kidney–lung, etc.) through blood circulation in the body. The recruitment of infiltrating CICs fighting pathogens leaked from gut contents would result in severe sepsis progression across remote multi-organs after the CASP-surgery, contributing to the newly observed phenomena: “systemic syndrome” in (**C**) treatment, “cytokine storm” in (**B**) treatment, and “KO MyD88-attenuation” in (**A**) treatment in the current study, which are discussed as follows.

### Systemic Syndrome

“Systemic inflammatory response syndrome (SIRS)” has been perceived to be an inflammatory state affecting the whole body, responding to an infectious or noninfectious insult. It should have both pro- and anti-inflammatory components although its definition is simply referred to an “inflammatory” response (57). The (**C**) treatment (compared septic wildtype mice with untreated wildtype mice) displays SIRS because of the strongest innate immune responses (**Tables 1**, **2**; **Figures 3**–**7**; **Supplementary Figures S1–S16**), which suggest possible emerging death from systemic syndromes. We term this newly observed fact in wildtype mice as “systemic syndrome” (**Figure 10**). Our findings endorse the previous report that septic wildtype mice had the highest mortality rate (24). Moreover, the identified and characterized 21 significant (*pGFdr <*0.0001) signaling pathways (**Table 1**), much beyond the one determined by previous bench-experiments (24), provide a broader vision at the systems-level of the innate immune responses triggered by the CASP-surgery as early as 12 hours after the surgery causing severe sepsis (**Table 1**; **Figure 1**). However, we remind that these target pathways would not necessarily mean any co-existing at the same time in a single event of CASP-surgery or across all patients simultaneously. Rather, they would be due to signaling-crosstalk at the systems-level, sharing a similar set of true key regulators across the entirety of target pathways (**Tables 1**, **2**), as marked on these target signaling pathways (**Figures 3**–**7**; **Supplementary Figures S1–S16**), which may result in an emerging death from systemic syndromes (at high risk for the same or undistinguishable cause) in clinical practices. These data also suggest that it is unlikely that such systemic syndromes can be precisely attributed to a specific type (viral, bacterial or parasitic) of infections, thus increasing the difficulty of early diagnosis and prevention of severe sepsis. Our findings provide novel insights into mechanisms underlying complicated syndromes in a broader vision, which warrant validations at the systems-level in the future.

### Cytokine Storm

“Cytokine storm” is a fatal immune reaction, consisting of a positive feedback loop between cytokines and white blood cells (58–61), which significantly elevates the levels of various cytokines (including chemokines, interleukins, interferons, tumor necrosis factors, and transforming growth factors). No consensus definition is accepted due to disagreement on the distinction between the cytokine storm and a physiologic inflammatory response (62). Cytokine storm is also known as cytokine cascade or hypercytokinemia, which defines the immune disorders with systemic inflammation, hyperinflammation, and multiple organ failure (58). The increased production of cytokines controls infections and the sustained excessive elevation in the levels of certain cytokines, cause negative systemic effects such as organ damage. In the present study, we obtained core regulators (see **Table 2**) once overlapped the DEGs from the (**A**), (**B**) and (**C**) treatments (**Supplementary Tables S1–S3**), which are involved in immune-cell growth, activation and function. These core regulators include **1**) ligands, receptors and adaptors (MyD88 and Lepr); **2**) signal transducers (Jak1~Jak3, Mapk1~Mapk3, Mapk8~Mapk14, Stat3, Rela, Jun, Fos, Mmp9, Osmr and Pdgfra); **3**) transcriptional factors (Nfkb1, Hif1a, Bcl3 and Eif3e); and **4**) downstream effectors, such as interleukins (Il1a, Il2ra, Il6ra and Il15ra), chemokines (Ccr1~Ccr10, Cxcr1~Cxcr6, Cx3cr1, Ccr1l1 and Xcr1), and colony-stimulating factors (Csf3r and Csf2rb). Our data highlight these core regulators intersected across all data (see AandBandC in **Table 2**) in both wildtype and deficient mice. Intriguingly, interferons (Ifna2, Ifna4~Ifna6, Ifna9, Ifna11~Ifna15, Ifnab, Ifnb1, Ifne, Ifng, Ifnk, Ifi206 and Ifi207) and tumor necrosis factors (Tnf and Tnfrsf1a) only belong to the (**B**) treatment (see Bonly in **Table 2**), unlike interleukins (Il1a, Il2ra Il6ra and Il15ra), chemokines (Ccr1~Ccr10, Cxcr1~Cxcr6, Ccl3, Cx3cr1, Ccr1l1 and Xcr1) and colony-stimulating factors (Csf2rb and Csf3r) that are intersected across the (**A**), (**B**) and (**C**) treatments. We term such newly observed facts as “cytokine-storm” (**Figure 10**) in the (**B**) treatment that compared septic KO MyD88 mice with untreated wildtype mice (**Table 2**), similar to that in viral infections (13, 58, 62, 63). The majority of these pro-inflammatory cytokines (**Table 2**) are newly characterized in the present study although some of them coincide with literature evidence, e.g., Ifng (interferon-γ) in the CASP-model sepsis (24, 27); CCRs and Il2ra (soluble interleukin-2 receptor alpha is a marker of T-cell activation) in Covid-19 disease (58). We recommend that such enriched cytokines warrant bench-experimental validations at the systems-level in the future.

### MyD88-Deficiency Attenuation

The (**C**) treatment compared septic wildtype mice with untreated wildtype mice, which produced 648 DEGs (**Supplementary Table S3**), provoking the strongest innate immune responses. However, the (**A**) treatment that compared septic KO MyD88 mice with septic wildtype mice produced 408 DEGs (**Supplementary Table S1**), and the (**B**) treatment that compared septic KO MyD88 mice with untreated wildtype mice produced 266 DEGs (**Supplementary Table S2**), both yielding less number of DEGs and provoking relatively weaker innate immune responses. Obviously, the wildtype mice in the (**C**) treatment produced much more DEGs than the KO MyD88 deficient mice in the (**A**) and (**B**) treatment, respectively. We term these newly observed facts as “MyD88-deficiency attenuation” (**Figure 10**). Our results suggest that the absence of MyD88 balances the innate immune responses in a favorable manner by attenuating deleterious responses, thus preventing excessive cytokine release while maintaining intact protective IFNs responsible for anti-viral, anti-bacterial, and/or anti-parasitic activities in severe septic peritonitis (see **Table 2**; **Figure 10**) and as such, the post-operative survival rates of mice were increased (24). We conclude that it is the excessive innate immune response causing excessive inflammation that leads to the rapid progression of sepsis at the systems-level, which results in the fatal failure of remote multi-organs and possible death from systemic syndromes in post-operative mice. In contrast, the KO MyD88-deficiency has attenuated the excessive inflammation by directly reducing the production of cytokines through having impaired the 21 MyD88-associated target signaling pathways (**Table 1**; **Figures 3**–**7**; **Supplementary Figures S1–S16**; **Figure 10**).

### Immune-Cell Infiltration

The majority of core regulators intersected across all data (see AandBandC in **Table 2**) are chemokines. Chemokines are conceived to recruit core immune cells (CICs) of the immune system (including B, CD4+T, CD8+T, macrophage, neutrophil, and dendritic cells) to the on-site of infection (64) or sterile injury (including tumor progression) (51). Although no databases are available for testing our perspectives, we have attempted on utilizing the database TIMER2.0 (51) of multiple cancer cohorts since local injury caused by tumor-progression promotes distant infiltration of CICs. Therefore, we have observed a positive, significant (*p*<0.001) correlation (**Figures 8**, **9**; **Supplementary Figures S17–S20**) between the expression level of key regulators (e.g., MyD88, Nfkb1, Ccr1, Csf2rb, Jak3, Il2ra, Csf3r and Ccr7) and the infiltration level of CICs in multiple cancer cohorts (e.g., COAD for colon, KIRC for kidney, LIHC for liver, and LUAD for lung). Our findings tentatively suggest that the infiltration of CICs likely plays a critical role in promoting the progression of the CASP-model sepsis at the systems-level as early as at 12 hours after the CASP-surgery.

### Central Role of Spleen

The spleen is the largest lymphatic tissue in the body and is a center for the production and storage of lymphocytes (B lymphocytes, T lymphocytes and NK cells) of the immune system (65). The structure of the spleen enables it to remove older erythrocytes from the circulation and leads to the efficient removal of blood-borne microorganisms and cellular debris because macrophages, neutrophils, and dendritic cells engulf and ingest such toxic agents (65). The red pulp area of the spleen breaks down toxic agents; the white pulp area of the spleen surrounding the splenic arteries is lymphatic tissue, providing a storehouse for lymphocytes (65). We infer that once the polymicrobial agents leaked from gut contents during the CASP-surgery, they were transferred into the spleen, and the degraded debris and the provoked downstream immune effectors were, thereby, transferred into remote multi-organs through circulation of the blood filtrated from the spleen. In fact, all phenomena we have observed in the present study had factually occurred in spleen, rather than in intestine where the CASP-surgery was performed, since GSE24327 GEO data were generated from samples recovered from the spleen 12 hours after the CASP-surgery (24). Taken together, we hypothesize that it is the spleen-mediated infiltration of CICs *via* blood circulation across remote multi-organs in the body that drives the rapid progression of the CASP-model sepsis across remote multi-organs at the systems-level as early as at 12 hours after the CASP-surgery in the body (**Figure 10**). In other words, infiltrating CICs across remote multi-organs (including colon, heart, spleen, kidney, liver and lung) would be achieved by enhancing the expression levels of those key regulators at the systems-level (**Table 2**; **Figures 8**, **9**). Such that, the infiltration of CICs would be recruited by those true key regulators (including chemokines) and would result in the rapid progression of CASP-model sepsis in the body (**Table 2**; **Figures 8**–**10**). Most importantly, there is likely a positive feedback loop among such consequent events of sepsis progression (**Figure 10**), thus causing a high mortality rate in wildtype mice (**C**). Such a positive feedback loop is also initiated in MyD88-deficient mice (**B**) at the early stage of sepsis progression (**Table 2**; **Figures 8**–**10**), but later attenuated owing to the impaired MyD88-assoicated signaling pathways, which keeps a balance between the moderate innate immunity responses and the living status of deficient mice (**B**), leading to a low mortality rate (24). We recommend these hypotheses be tested by bench-experiments at the systems-level in the future.

## Conclusion

This study aimed to elucidate systemic mechanisms underlying the standard CASP-model sepsis in the body through top-down data driven analysis on high-throughput functional genomics data. The novelty of this article roots in six aspects. 1) The in-house PathwayKO package is effective in conducting cutting-edge knockout pathway enrichment analysis in order to identify and assess the entirety of knockout signaling pathways at the systems-level. 2) The real-world GEO data (GSE24327_A, B and C) generated from the spleen 12 hours after the CASP-surgery safeguard unbiased choice of samples for the CASP-model sepsis in mice (both MyD88-deficient and wildtype). Therefore, we can discuss the full range of polymicrobial (viral, bacterial and parasitic) sepsis in provoking severe innate immune responses, resulting in failure of distant multi-organs (including colon, liver, kidney and lung) at the systems-level. 3) The 21 target KO MyD88-associated signaling pathways create a broader vision, providing novel insights into mechanisms underlying the CASP-model sepsis. 4) The true key regulators (ligands, receptors, adaptors, transducers, transcriptional factors, and cytokines) that were coordinately, significantly, and differentially expressed at the systems-level, as marked on those true target signaling pathways, provide massive potential biomarkers that warrant experimental validations in the future. 5) Based on the newly observed facts in the CASP-model sepsis, we discuss “systemic syndrome”, “cytokine storm”, and “KO MyD88 attenuation”, as well as the proposed hypothesis of “spleen-mediated immune-cell infiltration” at the systems-level. 6) Our results provide novel angles in a broader vision towards a better understanding of the CASP-model sepsis in mice, which may serve as a model for humans, to ultimately guide formulating the research paradigms and composite strategies for the early diagnosis and prevention of severe sepsis.

## Data Availability Statement

Publicly available datasets were analyzed in this study. This data can be found here: https://www.ncbi.nlm.nih.gov/geo/query/acc.cgi?acc=GSE24327.

## Author Contributions

HA and YA designed the project. HA conducted computations and analyzed data. HA, BL, FM, and YA interpreted results, wrote manuscript and approved the final manuscript.

## Funding

This work was supported by a grant-in-aid from National Science and Technology Major Programs of China (2014ZX0801105B-002) and Supercomputing Program of National Natural Science Foundation of China (No. U1501501-534) to YA.

## Conflict of Interest

Author HA was employed by SINOMACH-IT.

The remaining authors declare that the research was conducted in the absence of any commercial or financial relationships that could be construed as a potential conflict of interest.

## Publisher’s Note

All claims expressed in this article are solely those of the authors and do not necessarily represent those of their affiliated organizations, or those of the publisher, the editors and the reviewers. Any product that may be evaluated in this article, or claim that may be made by its manufacturer, is not guaranteed or endorsed by the publisher.

## Abbreviations

GEO, gene expression omnibus; KEGG, Kyoto encyclopedia of genes and genome; DEGs, differentially expressed genes; ROC, receiver operating characteristic; AUC, area under the ROC curve; pAUC_SP, partial AUC focusing on the specificity; pAUC_SE, partial AUC focusing on the sensitivity; HES, high-edge-scores; WT, wildtype; KO, knockout; TLR, toll-like receptor; MyD88, myeloid differentiating factor 88 (encoded by *Myd88*).
