# Supplementary material for: CASP-Model Sepsis Triggers Systemic Innate Immune Responses Revealed by the Systems-Level Signaling Pathways
Source: Front Immunol. 2022 Jun 14;13:907646. doi: 10.3389/fimmu.2022.907646 (PMC9238352; doi:10.3389/fimmu.2022.907646)
Supplement: Supplementary file 1 [file DataSheet_1.pdf]

Table S1 | GSE24327_A_RMA_Myd88_HES1_DEGs

	ENTREZID	SYMBOL	GENENAME
1	20310	Cxcl2	chemokine (C-X-C motif) ligand 2
2	12771	Ccr3	chemokine (C-C motif) receptor 3
3	12765	Cxcr2	chemokine (C-X-C motif) receptor 2
4	14825	Cxcl1	chemokine (C-X-C motif) ligand 1
5	12772	Ccr2	chemokine (C-C motif) receptor 2
6	20302	Ccl3	chemokine (C-C motif) ligand 3
7	330122	Cxcl3	chemokine (C-X-C motif) ligand 3
8	20311	Cxcl5	chemokine (C-X-C motif) ligand 5
9	20303	Ccl4	chemokine (C-C motif) ligand 4
10	23832	Xcr1	chemokine (C motif) receptor 1
11	12775	Ccr7	chemokine (C-C motif) receptor 7
12	15251	Hif1a	hypoxia inducible factor 1, alpha subunit
13	12770	Ccr1l1	chemokine (C-C motif) receptor 1-like 1
14	13051	Cx3cr1	chemokine (C-X3-C motif) receptor 1
15	12768	Ccr1	chemokine (C-C motif) receptor 1
16	12458	Ccr6	chemokine (C-C motif) receptor 6
17	54709	Eif3i	eukaryotic translation initiation factor 3, subunit I
18	26908	Eif2s3y	eukaryotic translation initiation factor 2, subunit 3, structural gene Y-linked
19	18018	Nfatc1	nuclear factor of activated T cells, cytoplasmic, calcineurin dependent 1
20	16193	Il6	interleukin 6
21	12986	Csf3r	colony stimulating factor 3 receptor (granulocyte)
22	12776	Ccr8	chemokine (C-C motif) receptor 8
23	16199	Il9r	interleukin 9 receptor
24	80901	Cxcr6	chemokine (C-X-C motif) receptor 6
25	20292	Ccl11	chemokine (C-C motif) ligand 11
26	18019	Nfatc2	nuclear factor of activated T cells, cytoplasmic, calcineurin dependent 2
27	16476	Jun	jun proto-oncogene
28	209354	Eif2b1	eukaryotic translation initiation factor 2B, subunit 1 (alpha)
29	224045	Eif2b5	eukaryotic translation initiation factor 2B, subunit 5 epsilon
30	16880	Lifr	LIF receptor alpha
31	53356	Eif3g	eukaryotic translation initiation factor 3, subunit G
32	12766	Cxcr3	chemokine (C-X-C motif) receptor 3
33	50931	Il27ra	interleukin 27 receptor, alpha
34	16341	Eif3e	eukaryotic translation initiation factor 3, subunit E
35	27979	Eif3b	eukaryotic translation initiation factor 3, subunit B
36	108067	Eif2b3	eukaryotic translation initiation factor 2B, subunit 3
37	12769	Ccr9	chemokine (C-C motif) receptor 9
38	55944	Eif3d	eukaryotic translation initiation factor 3, subunit D
39	217715	Eif2b2	eukaryotic translation initiation factor 2B, subunit 2 beta
40	100042807	Eif3j2	eukaryotic translation initiation factor 3, subunit J2
41	68135	Eif3h	eukaryotic translation initiation factor 3, subunit H
42	66085	Eif3f	eukaryotic translation initiation factor 3, subunit F
43	13667	Eif2b4	eukaryotic translation initiation factor 2B, subunit 4 delta
44	56347	Eif3c	eukaryotic translation initiation factor 3, subunit C
45	13669	Eif3a	eukaryotic translation initiation factor 3, subunit A
46	170786	Cd209a	CD209a antigen
47	16153	Il10	interleukin 10
48	12984	Csf2rb2	colony stimulating factor 2 receptor, beta 2, low-affinity (granulocyte-macrophage)
49	14281	Fos	FBJ osteosarcoma oncogene
50	20296	Ccl2	chemokine (C-C motif) ligand 2
51	26417	Mapk3	mitogen-activated protein kinase 3
52	17874	Myd88	myeloid differentiation primary response gene 88
53	12773	Ccr4	chemokine (C-C motif) receptor 4
54	213208	Il20rb	interleukin 20 receptor beta
55	16192	Il5ra	interleukin 5 receptor, alpha
56	26416	Mapk14	mitogen-activated protein kinase 14
57	12982	Csf2ra	colony stimulating factor 2 receptor, alpha, low-affinity (granulocyte-macrophage)
58	14283	Fosl1	fos-like antigen 1

59	12774	Ccr5	chemokine (C-C motif) receptor 5
60	16185	Il2rb	interleukin 2 receptor, beta chain
61	16155	Il10rb	interleukin 10 receptor, beta
62	20305	Ccl6	chemokine (C-C motif) ligand 6
63	20304	Ccl5	chemokine (C-C motif) ligand 5
64	12983	Csf2rb	colony stimulating factor 2 receptor, beta, low-affinity (granulocyte-macrophage)
65	16453	Jak3	Janus kinase 3
66	17395	Mmp9	matrix metallopeptidase 9
67	13614	Edn1	endothelin 1
68	13649	Egfr	epidermal growth factor receptor
69	27056	Irf5	interferon regulatory factor 5
70	16194	Il6ra	interleukin 6 receptor, alpha
71	60504	Il21r	interleukin 21 receptor
72	16186	Il2rg	interleukin 2 receptor, gamma chain
73	16847	Lepr	leptin receptor
74	73181	Nfatc4	nuclear factor of activated T cells, cytoplasmic, calcineurin dependent 4
75	16452	Jak2	Janus kinase 2
76	19697	Rela	v-rel reticuloendotheliosis viral oncogene homolog A (avian)
77	19094	Mapk11	mitogen-activated protein kinase 11
78	209590	Il23r	interleukin 23 receptor
79	56221	Ccl24	chemokine (C-C motif) ligand 24
80	18033	Nfkb1	nuclear factor of kappa light polypeptide gene enhancer in B cells 1, p105
81	18021	Nfatc3	nuclear factor of activated T cells, cytoplasmic, calcineurin dependent 3
82	12767	Cxcr4	chemokine (C-X-C motif) receptor 4
83	227288	Cxcr1	chemokine (C-X-C motif) receptor 1
84	12145	Cxcr5	chemokine (C-X-C motif) receptor 5
85	12777	Ccr10	chemokine (C-C motif) receptor 10
86	20848	Stat3	signal transducer and activator of transcription 3
87	16169	Il15ra	interleukin 15 receptor, alpha chain
88	16478	Jund	jun D proto-oncogene
89	57914	Crlf2	cytokine receptor-like factor 2
90	16175	Il1a	interleukin 1 alpha
91	16177	Il1r1	interleukin 1 receptor, type I
92	29857	Mapk12	mitogen-activated protein kinase 12
93	237310	Il22ra2	interleukin 22 receptor, alpha 2
94	16154	Il10ra	interleukin 10 receptor, alpha
95	15979	Ifngr1	interferon gamma receptor 1
96	230828	Il22ra1	interleukin 22 receptor, alpha 1
97	14282	Fosb	FBJ osteosarcoma oncogene B
98	17480	Mpl	myeloproliferative leukemia virus oncogene
99	16162	Il12rb2	interleukin 12 receptor, beta 2
100	16195	Il6st	interleukin 6 signal transducer
101	16164	Il13ra1	interleukin 13 receptor, alpha 1
102	19698	Relb	avian reticuloendotheliosis viral (v-rel) oncogene related B
103	16165	Il13ra2	interleukin 13 receptor, alpha 2
104	16161	Il12rb1	interleukin 12 receptor, beta 1
105	26415	Mapk13	mitogen-activated protein kinase 13
106	237313	Il20ra	interleukin 20 receptor, alpha
107	16197	Il7r	interleukin 7 receptor
108	26413	Mapk1	mitogen-activated protein kinase 1
109	12606	Cebpa	CCAAT/enhancer binding protein (C/EBP), alpha
110	16963	Xcl1	chemokine (C motif) ligand 1
111	18034	Nfkb2	nuclear factor of kappa light polypeptide gene enhancer in B cells 2, p49/p100
112	11535	Adm	adrenomedullin
113	56089	Ramp3	receptor (calcitonin) activity modifying protein 3
114	12985	Csf3	colony stimulating factor 3 (granulocyte)
115	15975	Ifnar1	interferon (alpha and beta) receptor 1
116	19116	Prlr	prolactin receptor
117	16184	Il2ra	interleukin 2 receptor, alpha chain
118	12051	Bcl3	B cell leukemia/lymphoma 3
119	16188	Il3ra	interleukin 3 receptor, alpha chain
120	15976	Ifnar2	interferon (alpha and beta) receptor 2

121	22695	Zfp36	zinc finger protein 36
122	20306	Ccl7	chemokine (C-C motif) ligand 7
123	16451	Jak1	Janus kinase 1
124	14600	Ghr	growth hormone receptor
125	26414	Mapk10	mitogen-activated protein kinase 10
126	18414	Osmr	oncostatin M receptor
127	18595	Pdgfra	platelet derived growth factor receptor, alpha polypeptide
128	26419	Mapk8	mitogen-activated protein kinase 8
129	26420	Mapk9	mitogen-activated protein kinase 9
130	16190	Il4ra	interleukin 4 receptor, alpha
131	12804	Cntfr	ciliary neurotrophic factor receptor
132	13857	Epor	erythropoietin receptor
133	242700	Ifnlr1	interferon lambda receptor 1
134	18596	Pdgfrb	platelet derived growth factor receptor, beta polypeptide
135	15980	Ifngr2	interferon gamma receptor 2
136	216869	Arrb2	arrestin, beta 2
137	55985	Cxcl13	chemokine (C-X-C motif) ligand 13
138	57349	Ppbp	pro-platelet basic protein
139	14677	Gnai1	guanine nucleotide binding protein (G protein), alpha inhibiting 1
140	12475	Cd14	CD14 antigen
141	21898	Tlr4	toll-like receptor 4
142	18787	Serpine1	serine (or cysteine) peptidase inhibitor, clade E, member 1
143	18791	Plat	plasminogen activator, tissue
144	20301	Ccl27a	chemokine (C-C motif) ligand 27A
145	18796	Plcb2	phospholipase C, beta 2
146	17329	Cxcl9	chemokine (C-X-C motif) ligand 9
147	20308	Ccl9	chemokine (C-C motif) ligand 9
148	14678	Gnai2	guanine nucleotide binding protein (G protein), alpha inhibiting 2
149	109689	Arrb1	arrestin, beta 1
150	57266	Cxcl14	chemokine (C-X-C motif) ligand 14
151	17533	Mrc1	mannose receptor, C type 1
152	20293	Ccl12	chemokine (C-C motif) ligand 12
153	20315	Cxcl12	chemokine (C-X-C motif) ligand 12
154	20312	Cx3cl1	chemokine (C-X3-C motif) ligand 1
155	320129	Grk3	G protein-coupled receptor kinase 3
156	20300	Ccl25	chemokine (C-C motif) ligand 25
157	110355	Grk2	G protein-coupled receptor kinase 2
158	20295	Ccl17	chemokine (C-C motif) ligand 17
159	14679	Gnai3	guanine nucleotide binding protein (G protein), alpha inhibiting 3
160	66102	Cxcl16	chemokine (C-X-C motif) ligand 16
161	20307	Ccl8	chemokine (C-C motif) ligand 8
162	13618	Ednrb	endothelin receptor type B
163	19225	Ptgs2	prostaglandin-endoperoxide synthase 2
164	15945	Cxcl10	chemokine (C-X-C motif) ligand 10
165	26385	Grk6	G protein-coupled receptor kinase 6
166	56066	Cxcl11	chemokine (C-X-C motif) ligand 11
167	541307	Ccl26	chemokine (C-C motif) ligand 26
168	56838	Ccl28	chemokine (C-C motif) ligand 28
169	16181	Il1rn	interleukin 1 receptor antagonist
170	20299	Ccl22	chemokine (C-C motif) ligand 22
171	56744	Pf4	platelet factor 4
172	84112	Sucnr1	succinate receptor 1
173	24013	Grk1	G protein-coupled receptor kinase 1
174	20290	Ccl1	chemokine (C-C motif) ligand 1
175	14773	Grk5	G protein-coupled receptor kinase 5
176	20297	Ccl20	chemokine (C-C motif) ligand 20
177	14772	Grk4	G protein-coupled receptor kinase 4
178	20309	Cxcl15	chemokine (C-X-C motif) ligand 15
179	14129	Fcgr1	Fc receptor, IgG, high affinity I
180	14128	Fcer2a	Fc receptor, IgE, low affinity II, alpha polypeptide
181	12333	Capn1	calpain 1
182	18797	Plcb3	phospholipase C, beta 3
183	24088	Tlr2	toll-like receptor 2
184	16180	Il1rap	interleukin 1 receptor accessory protein

185	233079	Ffar2	free fatty acid receptor 2
186	18126	Nos2	nitric oxide synthase 2, inducible
187	107607	Nod1	nucleotide-binding oligomerization domain containing 1
188	21857	Timp1	tissue inhibitor of metalloproteinase 1
189	20852	Stat6	signal transducer and activator of transcription 6
190	14674	Gna13	guanine nucleotide binding protein, alpha 13
191	18798	Plcb4	phospholipase C, beta 4
192	19416	Rasd1	RAS, dexamethasone-induced 1
193	18747	Prkaca	protein kinase, cAMP dependent, catalytic, alpha
194	17087	Ly96	lymphocyte antigen 96
195	170776	Cd209c	CD209c antigen
196	20375	Spi1	spleen focus forming virus (SFFV) proviral integration oncogene
197	21950	Tnfsf9	tumor necrosis factor (ligand) superfamily, member 9
198	21942	Tnfrsf9	tumor necrosis factor receptor superfamily, member 9
199	54393	Gabbr1	gamma-aminobutyric acid (GABA) B receptor, 1
200	20969	Sdc1	syndecan 1
201	18788	Serpinb2	serine (or cysteine) peptidase inhibitor, clade B, member 2
202	239081	Tlr11	toll-like receptor 11
203	66513	Tab1	TGF-beta activated kinase 1/MAP3K7 binding protein 1
204	18125	Nos1	nitric oxide synthase 1, neuronal
205	170779	Cd209d	CD209d antigen
206	16598	Klf2	Kruppel-like factor 2 (lung)
207	12334	Capn2	calpain 2
208	14463	Gata4	GATA binding protein 4
209	21859	Timp3	tissue inhibitor of metalloproteinase 3
210	14702	Gng2	guanine nucleotide binding protein (G protein), gamma 2
211	19664	Rbpj	recombination signal binding protein for immunoglobulin kappa J region
212	80885	Hcar2	hydroxycarboxylic acid receptor 2
213	21825	Thbs1	thrombospondin 1
214	15200	Hbegf	heparin-binding EGF-like growth factor
215	170780	Cd209e	CD209e antigen
216	12519	Cd80	CD80 antigen
217	12477	Ctla4	cytotoxic T-lymphocyte-associated protein 4
218	16178	Il1r2	interleukin 1 receptor, type II
219	18795	Plcb1	phospholipase C, beta 1
220	69165	Cd209b	CD209b antigen
221	14701	Gng12	guanine nucleotide binding protein (G protein), gamma 12
222	54140	Avpr1a	arginine vasopressin receptor 1A
223	19204	Ptafr	platelet-activating factor receptor
224	14681	Gnao1	guanine nucleotide binding protein, alpha O
225	224129	Adcy5	adenylate cyclase 5
226	14131	Fcgr3	Fc receptor, IgG, low affinity III
227	70192	Cd209g	CD209g antigen
228	17534	Mrc2	mannose receptor, C type 2
229	18779	Pla2r1	phospholipase A2 receptor 1
230	17390	Mmp2	matrix metallopeptidase 2
231	69142	Cd209f	CD209f antigen
232	100038947	Sirpb1c	signal-regulatory protein beta 1C
233	20963	Syk	spleen tyrosine kinase
234	11998	Avp	arginine vasopressin
235	243270	Hcar1	hydrocarboxylic acid receptor 1
236	12061	Bdkrb1	bradykinin receptor, beta 1
237	12062	Bdkrb2	bradykinin receptor, beta 2
238	13617	Ednra	endothelin receptor type A
239	15170	Ptpn6	protein tyrosine phosphatase, non-receptor type 6
240	64337	Gng13	guanine nucleotide binding protein (G protein), gamma 13
241	14693	Gnb2	guanine nucleotide binding protein (G protein), beta 2
242	14710	Gngt2	guanine nucleotide binding protein (G protein), gamma transducing activity polypeptide 2
243	23939	Mapk7	mitogen-activated protein kinase 7
244	50723	Icosl	icos ligand
245	14061	F2	coagulation factor II
246	14673	Gna12	guanine nucleotide binding protein, alpha 12
247	239556	Cacna1i	calcium channel, voltage-dependent, alpha 1I subunit

248	19395	Rasgrp2	RAS, guanyl releasing protein 2
249	140570	Plxnb2	plexin B2
250	223881	Rnd1	Rho family GTPase 1
251	11606	Agt	angiotensinogen (serpin peptidase inhibitor, clade A, member 8)
252	14683	Gnas	GNAS (guanine nucleotide binding protein, alpha stimulating) complex locus
253	14704	Gng3	guanine nucleotide binding protein (G protein), gamma 3
254	328572	Ep300	E1A binding protein p300
255	19668	Rbpjl	recombination signal binding protein for immunoglobulin kappa J region-like
256	19218	Ptger3	prostaglandin E receptor 3 (subtype EP3)
257	11555	Adrb2	adrenergic receptor, beta 2
258	15557	Htr1f	5-hydroxytryptamine (serotonin) receptor 1F
259	17969	Ncf1	neutrophil cytosolic factor 1
260	70729	Nos1ap	nitric oxide synthase 1 (neuronal) adaptor protein
261	109648	Npy	neuropeptide Y
262	14697	Gnb5	guanine nucleotide binding protein (G protein), beta 5
263	15552	Htr1d	5-hydroxytryptamine (serotonin) receptor 1D
264	14700	Gng10	guanine nucleotide binding protein (G protein), gamma 10
265	18749	Prkacb	protein kinase, cAMP dependent, catalytic, beta
266	14706	Gng4	guanine nucleotide binding protein (G protein), gamma 4
267	11863	Arnt	aryl hydrocarbon receptor nuclear translocator
268	66066	Gng11	guanine nucleotide binding protein (G protein), gamma 11
269	14708	Gng7	guanine nucleotide binding protein (G protein), gamma 7
270	14695	Gnb3	guanine nucleotide binding protein (G protein), beta 3
271	14707	Gng5	guanine nucleotide binding protein (G protein), gamma 5
272	20605	Sstr1	somatostatin receptor 1
273	12914	Crebbp	CREB binding protein
274	208188	Ghsr	growth hormone secretagogue receptor
275	235320	Zbtb16	zinc finger and BTB domain containing 16
276	16480	Jup	junction plakoglobin
277	21929	Tnfaip3	tumor necrosis factor, alpha-induced protein 3
278	14696	Gnb4	guanine nucleotide binding protein (G protein), beta 4
279	14699	Gngt1	guanine nucleotide binding protein (G protein), gamma transducing activity polypeptide 1
280	14688	Gnb1	guanine nucleotide binding protein (G protein), beta 1
281	14709	Gng8	guanine nucleotide binding protein (G protein), gamma 8
282	13489	Drd2	dopamine receptor D2
283	18024	Nfe2l2	nuclear factor, erythroid derived 2, like 2
284	18815	Plg	plasminogen
285	11539	Adora1	adenosine A1 receptor
286	242425	Gabbr2	gamma-aminobutyric acid (GABA) B receptor, 2
287	12310	Calca	calcitonin/calcitonin-related polypeptide, alpha
288	12669	Chrm1	cholinergic receptor, muscarinic 1, CNS
289	116903	Calcb	calcitonin-related polypeptide, beta
290	20606	Sstr2	somatostatin receptor 2
291	18166	Npy1r	neuropeptide Y receptor Y1
292	15551	Htr1b	5-hydroxytryptamine (serotonin) receptor 1B
293	243764	Chrm2	cholinergic receptor, muscarinic 2, cardiac
294	18430	Oxtr	oxytocin receptor
295	60533	Cd274	CD274 antigen
296	18566	Pdcd1	programmed cell death 1
297	20609	Sstr5	somatostatin receptor 5
298	18845	Plxna2	plexin A2
299	16409	Itgam	integrin alpha M
300	15550	Htr1a	5-hydroxytryptamine (serotonin) receptor 1A
301	246256	Fcgr4	Fc receptor, IgG, low affinity IV
302	17972	Ncf4	neutrophil cytosolic factor 4
303	21687	Tek	TEK receptor tyrosine kinase
304	12946	Cr1l	complement component (3b/4b) receptor 1-like
305	16415	Itgb2l	integrin beta 2-like
306	387194	Mir199a-1	microRNA 199a-1
307	16414	Itgb2	integrin beta 2
308	21803	Tgfb1	transforming growth factor, beta 1

309	235611	Plxnb1	plexin B1
310	67405	Nts	neurotensin
311	18217	Ntsr2	neurotensin receptor 2
312	15042	H2-T24	histocompatibility 2, T region locus 24
313	12525	Cd8a	CD8 antigen, alpha chain
314	20698	Sphk1	sphingosine kinase 1
315	18216	Ntsr1	neurotensin receptor 1
316	12443	Ccnd1	cyclin D1
317	21923	Tnc	tenascin C
318	20971	Sdc4	syndecan 4
319	26427	Creb3l1	cAMP responsive element binding protein 3-like 1
320	14608	Gpr83	G protein-coupled receptor 83
321	54635	Pdgfc	platelet-derived growth factor, C polypeptide
322	14675	Gna14	guanine nucleotide binding protein, alpha 14
323	18792	Plau	plasminogen activator, urokinase
324	18793	Plaur	plasminogen activator, urokinase receptor
325	53945	Slc40a1	solute carrier family 40 (iron-regulated transporter), member 1
326	15203	Heph	hephaestin
327	12293	Cacna2d1	calcium channel, voltage-dependent, alpha2/delta subunit 1
328	12902	Cr2	complement receptor 2
329	231991	Creb5	cAMP responsive element binding protein 5
330	15234	Hgf	hepatocyte growth factor
331	16612	Klk1	kallikrein 1
332	14256	Flt3l	FMS-like tyrosine kinase 3 ligand
333	12389	Cav1	caveolin 1, caveolae protein
334	20849	Stat4	signal transducer and activator of transcription 4
335	12702	Socs3	suppressor of cytokine signaling 3
336	233046	Rasgrp4	RAS guanyl releasing protein 4
337	17970	Ncf2	neutrophil cytosolic factor 2
338	16421	Itgb7	integrin beta 7
339	12575	Cdkn1a	cyclin-dependent kinase inhibitor 1A (P21)
340	170768	Pfkfb3	6-phosphofructo-2-kinase/fructose-2,6-biphosphatase 3
341	22034	Traf6	TNF receptor-associated factor 6
342	12290	Cacna1e	calcium channel, voltage-dependent, R type, alpha 1E subunit
343	14672	Gna11	guanine nucleotide binding protein, alpha 11
344	16160	Il12b	interleukin 12b
345	226421	Rab7b	RAB7B, member RAS oncogene family
346	56455	Dynll1	dynein light chain LC8-type 1
347	26399	Map2k6	mitogen-activated protein kinase kinase 6
348	12265	Ciita	class II transactivator
349	15000	H2-DMb2	histocompatibility 2, class II, locus Mb2
350	16000	Igf1	insulin-like growth factor 1
351	18049	Ngf	nerve growth factor
352	21808	Tgfb2	transforming growth factor, beta 2
353	64099	Parvg	parvin, gamma
354	14127	Fcer1g	Fc receptor, IgE, high affinity I, gamma polypeptide
355	17086	Ncr1	natural cytotoxicity triggering receptor 1
356	12524	Cd86	CD86 antigen
357	16324	Inhbb	inhibin beta-B
358	11480	Acvr2a	activin receptor IIA
359	16985	Lsp1	lymphocyte specific 1
360	56532	Ripk3	receptor-interacting serine-threonine kinase 3
361	108099	Prkag2	protein kinase, AMP-activated, gamma 2 non-catalytic subunit
362	11601	Angpt2	angiopoietin 2
363	13057	Cyba	cytochrome b-245, alpha polypeptide
364	12562	Cdh5	cadherin 5
365	67373	2210010C04Rik	RIKEN cDNA 2210010C04 gene
366	14065	F2rl3	coagulation factor II (thrombin) receptor-like 3
367	208650	Cblb	Casitas B-lineage lymphoma b
368	56644	Clec7a	C-type lectin domain family 7, member a
369	15002	H2-Ob	histocompatibility 2, O region beta locus
370	20779	Src	Rous sarcoma oncogene
371	21809	Tgfb3	transforming growth factor, beta 3
372	14676	Gna15	guanine nucleotide binding protein, alpha 15

373	16643	Klrd1	killer cell lectin-like receptor, subfamily D, member 1
374	22339	Vegfa	vascular endothelial growth factor A
375	22329	Vcam1	vascular cell adhesion molecule 1
376	70686	Dusp16	dual specificity phosphatase 16
377	108097	Prkab2	protein kinase, AMP-activated, beta 2 non-catalytic subunit
378	67951	Tubb6	tubulin, beta 6 class V
379	15001	H2-Oa	histocompatibility 2, O region alpha locus
380	18846	Plxna3	plexin A3
381	18053	Ngfr	nerve growth factor receptor (TNFR superfamily, member 16)
382	73914	Irak3	interleukin-1 receptor-associated kinase 3
383	12649	Chek1	checkpoint kinase 1
384	22059	Trp53	transformation related protein 53
385	12912	Creb1	cAMP responsive element binding protein 1
386	329934	Foxo6	forkhead box O6
387	20620	Plk2	polo like kinase 2
388	12915	Atf6b	activating transcription factor 6 beta
389	112406	Egln2	egl-9 family hypoxia-inducible factor 2
390	54721	Tyk2	tyrosine kinase 2
391	18844	Plxna1	plexin A1
392	11479	Acvr1b	activin A receptor, type 1B
393	69632	Arhgef12	Rho guanine nucleotide exchange factor (GEF) 12
394	21416	Tcf7l2	transcription factor 7 like 2, T cell specific, HMG box
395	18751	Prkcb	protein kinase C, beta
396	19016	Pparg	peroxisome proliferator activated receptor gamma
397	11770	Fabp4	fatty acid binding protein 4, adipocyte
398	12503	Cd247	CD247 antigen
399	11848	Rhoa	ras homolog family member A
400	53608	Map3k6	mitogen-activated protein kinase kinase kinase 6
401	67653	4930544G11Rik	RIKEN cDNA 4930544G11 gene
402	12795	Plk3	polo like kinase 3
403	16706	Ksr1	kinase suppressor of ras 1
404	14186	Fgfr4	fibroblast growth factor receptor 4
405	14366	Fzd4	frizzled class receptor 4
406	14313	Fst	follistatin
407	12390	Cav2	caveolin 2
408	18806	Pld2	phospholipase D2

Table S2 | GSE24327_B_RMA_Myd88_HES1_DEGs

	ENTREZID	SYMBOL	GENENAME
1	54123	Irf7	interferon regulatory factor 7
2	13669	Eif3a	eukaryotic translation initiation factor 3, subunit A
3	26908	Eif2s3y	eukaryotic translation initiation factor 2, subunit 3, structural gene Y-linked
4	27979	Eif3b	eukaryotic translation initiation factor 3, subunit B
5	15945	Cxcl10	chemokine (C-X-C motif) ligand 10
6	55944	Eif3d	eukaryotic translation initiation factor 3, subunit D
7	56347	Eif3c	eukaryotic translation initiation factor 3, subunit C
8	224045	Eif2b5	eukaryotic translation initiation factor 2B, subunit 5 epsilon
9	217715	Eif2b2	eukaryotic translation initiation factor 2B, subunit 2 beta
10	68135	Eif3h	eukaryotic translation initiation factor 3, subunit H
11	58203	Zbp1	Z-DNA binding protein 1
12	209354	Eif2b1	eukaryotic translation initiation factor 2B, subunit 1 (alpha)
13	53356	Eif3g	eukaryotic translation initiation factor 3, subunit G
14	66085	Eif3f	eukaryotic translation initiation factor 3, subunit F
15	54709	Eif3i	eukaryotic translation initiation factor 3, subunit I
16	108067	Eif2b3	eukaryotic translation initiation factor 2B, subunit 3
17	100042807	Eif3j2	eukaryotic translation initiation factor 3, subunit J2
18	13667	Eif2b4	eukaryotic translation initiation factor 2B, subunit 4 delta
19	16341	Eif3e	eukaryotic translation initiation factor 3, subunit E
20	19106	Eif2ak2	eukaryotic translation initiation factor 2-alpha kinase 2
21	15957	Ifit1	interferon-induced protein with tetratricopeptide repeats 1
22	20847	Stat2	signal transducer and activator of transcription 2
23	17858	Mx2	MX dynamin-like GTPase 2
24	246728	Oas2	2'-5' oligoadenylate synthetase 2
25	16176	Il1b	interleukin 1 beta
26	16178	Il1r2	interleukin 1 receptor, type II
27	246727	Oas3	2'-5' oligoadenylate synthetase 3
28	16181	Il1rn	interleukin 1 receptor antagonist
29	12768	Ccr1	chemokine (C-C motif) receptor 1
30	231655	Oasl1	2'-5' oligoadenylate synthetase-like 1
31	23961	Oas1b	2'-5' oligoadenylate synthetase 1B
32	23962	Oasl2	2'-5' oligoadenylate synthetase-like 2
33	15042	H2-T24	histocompatibility 2, T region locus 24
34	12575	Cdkn1a	cyclin-dependent kinase inhibitor 1A (P21)
35	20293	Ccl12	chemokine (C-C motif) ligand 12
36	20846	Stat1	signal transducer and activator of transcription 1
37	12765	Cxcr2	chemokine (C-X-C motif) receptor 2
38	16391	Irf9	interferon regulatory factor 9
39	56619	Clec4e	C-type lectin domain family 4, member e
40	17474	Clec4d	C-type lectin domain family 4, member d
41	12702	Socs3	suppressor of cytokine signaling 3
42	16175	Il1a	interleukin 1 alpha
43	12051	Bcl3	B cell leukemia/lymphoma 3
44	20299	Ccl22	chemokine (C-C motif) ligand 22
45	80861	Dhx58	DEXH (Asp-Glu-X-His) box polypeptide 58
46	71586	Ifih1	interferon induced with helicase C domain 1
47	15467	Eif2ak1	eukaryotic translation initiation factor 2 alpha kinase 1
48	19697	Rela	v-rel reticuloendotheliosis viral oncogene homolog A (avian)
49	18033	Nfkb1	nuclear factor of kappa light polypeptide gene enhancer in B cells 1, p105
50	26409	Map3k7	mitogen-activated protein kinase kinase kinase 7
51	26408	Map3k5	mitogen-activated protein kinase kinase kinase 5
52	12774	Ccr5	chemokine (C-C motif) receptor 5
53	17329	Cxcl9	chemokine (C-X-C motif) ligand 9
54	246730	Oas1a	2'-5' oligoadenylate synthetase 1A
55	26406	Map3k3	mitogen-activated protein kinase kinase kinase 3
56	27103	Eif2ak4	eukaryotic translation initiation factor 2 alpha kinase 4
57	13666	Eif2ak3	eukaryotic translation initiation factor 2 alpha kinase 3
58	230073	Ddx58	DEAD (Asp-Glu-Ala-Asp) box polypeptide 58
59	12458	Ccr6	chemokine (C-C motif) receptor 6
60	23832	Xcr1	chemokine (C motif) receptor 1

61	328572	Ep300	E1A binding protein p300
62	16179	Irak1	interleukin-1 receptor-associated kinase 1
63	69550	Bst2	bone marrow stromal cell antigen 2
64	22034	Traf6	TNF receptor-associated factor 6
65	16476	Jun	jun proto-oncogene
66	15969	Ifna6	interferon alpha 6
67	242519	Ifna12	interferon alpha 12
68	12675	Chuk	conserved helix-loop-helix ubiquitous kinase
69	80901	Cxcr6	chemokine (C-X-C motif) receptor 6
70	56489	Ikbke	inhibitor of kappaB kinase epsilon
71	56480	Tbk1	TANK-binding kinase 1
72	13051	Cx3cr1	chemokine (C-X3-C motif) receptor 1
73	17869	Myc	myelocytomatosis oncogene
74	12767	Cxcr4	chemokine (C-X-C motif) receptor 4
75	15977	Ifnb1	interferon beta 1, fibroblast
76	23960	Oas1g	2'-5' oligoadenylate synthetase 1G
77	242517	Ifna15	interferon alpha 15
78	15974	Ifnab	interferon alpha B
79	21926	Tnf	tumor necrosis factor
80	12914	Crebbp	CREB binding protein
81	387510	Ifnk	interferon kappa
82	15972	Ifna9	interferon alpha 9
83	230405	Ifne	interferon epsilon
84	12771	Ccr3	chemokine (C-C motif) receptor 3
85	23797	Akt3	thymoma viral proto-oncogene 3
86	230396	Ifna13	interferon alpha 13
87	106759	Ticam1	toll-like receptor adaptor molecule 1
88	15968	Ifna5	interferon alpha 5
89	12769	Ccr9	chemokine (C-C motif) receptor 9
90	19766	Ripk1	receptor (TNFRSF)-interacting serine-threonine kinase 1
91	15967	Ifna4	interferon alpha 4
92	15978	Ifng	interferon gamma
93	16168	Il15	interleukin 15
94	18414	Osmr	oncostatin M receptor
95	12773	Ccr4	chemokine (C-C motif) receptor 4
96	54131	Irf3	interferon regulatory factor 3
97	266632	Irak4	interleukin-1 receptor-associated kinase 4
98	15965	Ifna2	interferon alpha 2
99	20750	Spp1	secreted phosphoprotein 1
100	15039	H2-T22	histocompatibility 2, T region locus 22
101	11651	Akt1	thymoma viral proto-oncogene 1
102	14281	Fos	FBJ osteosarcoma oncogene
103	11652	Akt2	thymoma viral proto-oncogene 2
104	12700	Cish	cytokine inducible SH2-containing protein
105	12145	Cxcr5	chemokine (C-X-C motif) receptor 5
106	16145	Igtp	interferon gamma induced GTPase
107	15964	Ifna11	interferon alpha 11
108	56532	Ripk3	receptor-interacting serine-threonine kinase 3
109	15944	Irgm1	immunity-related GTPase family M member 1
110	16453	Jak3	Janus kinase 3
111	404549	Ifna14	interferon alpha 14
112	16151	Ikbkg	inhibitor of kappaB kinase gamma
113	12770	Ccr1l1	chemokine (C-C motif) receptor 1-like 1
114	12777	Ccr10	chemokine (C-C motif) receptor 10
115	12772	Ccr2	chemokine (C-C motif) receptor 2
116	20848	Stat3	signal transducer and activator of transcription 3
117	12775	Ccr7	chemokine (C-C motif) receptor 7
118	12766	Cxcr3	chemokine (C-X-C motif) receptor 3
119	12776	Ccr8	chemokine (C-C motif) receptor 8
120	227288	Cxcr1	chemokine (C-X-C motif) receptor 1
121	16184	Il2ra	interleukin 2 receptor, alpha chain
122	12983	Csf2rb	colony stimulating factor 2 receptor, beta, low-affinity (granulocyte-macrophage)
123	16169	Il15ra	interleukin 15 receptor, alpha chain

124	12444	Ccnd2	cyclin D2
125	16452	Jak2	Janus kinase 2
126	20308	Ccl9	chemokine (C-C motif) ligand 9
127	17395	Mmp9	matrix metallopeptidase 9
128	12703	Socs1	suppressor of cytokine signaling 1
129	12445	Ccnd3	cyclin D3
130	110696	H2-M10.3	histocompatibility 2, M region locus 10.3
131	18854	Pml	promyelocytic leukemia
132	15040	H2-T23	histocompatibility 2, T region locus 23
133	12571	Cdk6	cyclin-dependent kinase 6
134	20852	Stat6	signal transducer and activator of transcription 6
135	18024	Nfe2l2	nuclear factor, erythroid derived 2, like 2
136	19252	Dusp1	dual specificity phosphatase 1
137	15024	H2-T10	histocompatibility 2, T region locus 10
138	22059	Trp53	transformation related protein 53
139	83383	Tfap4	transcription factor AP4
140	333715	H2-M10.2	histocompatibility 2, M region locus 10.2
141	110557	H2-Q6	histocompatibility 2, Q region locus 6
142	15015	H2-Q4	histocompatibility 2, Q region locus 4
143	56198	Heyl	hairy/enhancer-of-split related with YRPW motif-like
144	387139	Mir20a	microRNA 20a
145	26417	Mapk3	mitogen-activated protein kinase 3
146	21937	Tnfrsf1a	tumor necrosis factor receptor superfamily, member 1a
147	13163	Daxx	Fas death domain-associated protein
148	15251	Hif1a	hypoxia inducible factor 1, alpha subunit
149	209387	Trim30d	tripartite motif-containing 30D
150	208647	Creb3l2	cAMP responsive element binding protein 3-like 2
151	12915	Atf6b	activating transcription factor 6 beta
152	17128	Smad4	SMAD family member 4
153	17127	Smad3	SMAD family member 3
154	242316	Gdf6	growth differentiation factor 6
155	12168	Bmpr2	bone morphogenetic protein receptor, type II (serine/threonine kinase)
156	15208	Hes5	hes family bHLH transcription factor 5
157	15018	H2-Q7	histocompatibility 2, Q region locus 7
158	12443	Ccnd1	cyclin D1
159	329934	Foxo6	forkhead box O6
160	11911	Atf4	activating transcription factor 4
161	217069	Trim25	tripartite motif-containing 25
162	26413	Mapk1	mitogen-activated protein kinase 1
163	14972	H2-K1	histocompatibility 2, K1, K region
164	15214	Hey2	hairy/enhancer-of-split related with YRPW motif 2
165	20302	Ccl3	chemokine (C-C motif) ligand 3
166	224753	H2-M10.4	histocompatibility 2, M region locus 10.4
167	16337	Insr	insulin receptor
168	15006	H2-Q1	histocompatibility 2, Q region locus 1
169	15013	H2-Q2	histocompatibility 2, Q region locus 2
170	17126	Smad2	SMAD family member 2
171	667977	Gm8909	predicted gene 8909
172	208677	Creb3l3	cAMP responsive element binding protein 3-like 3
173	15213	Hey1	hairy/enhancer-of-split related with YRPW motif 1
174	15043	H2-T3	histocompatibility 2, T region locus 3
175	18712	Pim1	proviral integration site 1
176	11909	Atf2	activating transcription factor 2
177	16847	Lepr	leptin receptor
178	56066	Cxcl11	chemokine (C-X-C motif) ligand 11
179	224756	H2-M1	histocompatibility 2, M region locus 1
180	11863	Arnt	aryl hydrocarbon receptor nuclear translocator
181	224754	H2-M11	histocompatibility 2, M region locus 11
182	56458	Foxo1	forkhead box O1
183	14963	H2-Bl	histocompatibility 2, blastocyst
184	226691	Ifi207	interferon activated gene 207
185	72512	Tmem173	transmembrane protein 173
186	69717	Gm10499	predicted gene 10499
187	56438	Rbx1	ring-box 1

188	26420	Mapk9	mitogen-activated protein kinase 9
189	12448	Ccne2	cyclin E2
190	12913	Creb3	cAMP responsive element binding protein 3
191	22640	Zfp1	zinc finger protein 1
192	399549	H2-M10.6	histocompatibility 2, M region locus 10.6
193	14985	H2-M10.1	histocompatibility 2, M region locus 10.1
194	18595	Pdgfra	platelet derived growth factor receptor, alpha polypeptide
195	14991	H2-M3	histocompatibility 2, M region locus 3
196	14964	H2-D1	histocompatibility 2, D region locus 1
197	12567	Cdk4	cyclin-dependent kinase 4
198	15951	Ifi204	interferon activated gene 204
199	14990	H2-M2	histocompatibility 2, M region locus 2
200	21356	Tapbp	TAP binding protein
201	12427	Ccna1	cyclin A1
202	20851	Stat5b	signal transducer and activator of transcription 5B
203	27401	Skp2	S-phase kinase-associated protein 2 (p45)
204	56484	Foxo3	forkhead box O3
205	224761	H2-M10.5	histocompatibility 2, M region locus 10.5
206	17210	Mcl1	myeloid cell leukemia sequence 1
207	16194	Il6ra	interleukin 6 receptor, alpha
208	667803	H2-T-ps	histocompatibility 2, T region locus, pseudogene
209	231991	Creb5	cAMP responsive element binding protein 5
210	26414	Mapk10	mitogen-activated protein kinase 10
211	15205	Hes1	hes family bHLH transcription factor 1
212	29857	Mapk12	mitogen-activated protein kinase 12
213	240095	H2-M5	histocompatibility 2, M region locus 5
214	14939	Gzmb	granzyme B
215	192157	Socs7	suppressor of cytokine signaling 7
216	26965	Cul1	cullin 1
217	12912	Creb1	cAMP responsive element binding protein 1
218	723925	Mir106b	microRNA 106b
219	384783	Irs2	insulin receptor substrate 2
220	14127	Fcer1g	Fc receptor, IgE, high affinity I, gamma polypeptide
221	56717	Mtor	mechanistic target of rapamycin kinase
222	21402	Skp1a	S-phase kinase-associated protein 1A
223	12428	Ccna2	cyclin A2
224	16370	Irs4	insulin receptor substrate 4
225	26419	Mapk8	mitogen-activated protein kinase 8
226	12566	Cdk2	cyclin-dependent kinase 2
227	14997	H2-M9	histocompatibility 2, M region locus 9
228	26416	Mapk14	mitogen-activated protein kinase 14
229	78284	Creb3l4	cAMP responsive element binding protein 3-like 4
230	108012	Ap1s2	adaptor-related protein complex 1, sigma 2 subunit
231	12447	Ccne1	cyclin E1
232	26415	Mapk13	mitogen-activated protein kinase 13
233	26427	Creb3l1	cAMP responsive element binding protein 3-like 1
234	18121	Nog	noggin
235	12367	Casp3	caspase 3
236	15007	H2-Q10	histocompatibility 2, Q region locus 10
237	20849	Stat4	signal transducer and activator of transcription 4
238	54601	Foxo4	forkhead box O4
239	20850	Stat5a	signal transducer and activator of transcription 5A
240	17874	Myd88	myeloid differentiation primary response gene 88
241	58185	Rsad2	radical S-adenosyl methionine domain containing 2
242	11481	Acvr2b	activin receptor IIB
243	51792	Ppp2r1a	protein phosphatase 2, regulatory subunit A, alpha
244	12166	Bmpr1a	bone morphogenetic protein receptor, type 1A
245	17965	Nbl1	NBL1, DAN family BMP antagonist
246	19094	Mapk11	mitogen-activated protein kinase 11
247	243308	A430033K04Rik	RIKEN cDNA A430033K04 gene
248	20963	Syk	spleen tyrosine kinase
249	11765	Ap1g1	adaptor protein complex AP-1, gamma 1 subunit
250	16150	Ikbkb	inhibitor of kappaB kinase beta
251	16451	Jak1	Janus kinase 1

252	12804	Cntfr	ciliary neurotrophic factor receptor
253	23892	Grem1	gremlin 1, DAN family BMP antagonist
254	269643	Ppp2r2c	protein phosphatase 2, regulatory subunit B, gamma
255	668620	Zfp936	zinc finger protein 936
256	102639543	Ifi206	interferon activated gene 206
257	12167	Bmpr1b	bone morphogenetic protein receptor, type 1B
258	54678	Zfp108	zinc finger protein 108
259	66824	Pycard	PYD and CARD domain containing
260	56469	Pias1	protein inhibitor of activated STAT 1
261	54721	Tyk2	tyrosine kinase 2
262	16643	Klrd1	killer cell lectin-like receptor, subfamily D, member 1
263	12986	Csf3r	colony stimulating factor 3 receptor (granulocyte)
264	110542	Amhr2	anti-Mullerian hormone type 2 receptor
265	408067	Zfp874b	zinc finger protein 874b
266	54607	Socs6	suppressor of cytokine signaling 6

Table S3 | GSE24327_C_RMA_Myd88_HES1_DEGs

	ENTREZID	SYMBOL	GENENAME
1	20310	Cxcl2	chemokine (C-X-C motif) ligand 2
2	12771	Ccr3	chemokine (C-C motif) receptor 3
3	20302	Ccl3	chemokine (C-C motif) ligand 3
4	23832	Xcr1	chemokine (C motif) receptor 1
5	14825	Cxcl1	chemokine (C-X-C motif) ligand 1
6	20303	Ccl4	chemokine (C-C motif) ligand 4
7	20311	Cxcl5	chemokine (C-X-C motif) ligand 5
8	330122	Cxcl3	chemokine (C-X-C motif) ligand 3
9	15945	Cxcl10	chemokine (C-X-C motif) ligand 10
10	12772	Ccr2	chemokine (C-C motif) receptor 2
11	13051	Cx3cr1	chemokine (C-X3-C motif) receptor 1
12	15251	Hif1a	hypoxia inducible factor 1, alpha subunit
13	16175	Il1a	interleukin 1 alpha
14	16178	Il1r2	interleukin 1 receptor, type II
15	80901	Cxcr6	chemokine (C-X-C motif) receptor 6
16	16476	Jun	jun proto-oncogene
17	16193	Il6	interleukin 6
18	16169	Il15ra	interleukin 15 receptor, alpha chain
19	16181	Il1rn	interleukin 1 receptor antagonist
20	18414	Osmr	oncostatin M receptor
21	12765	Cxcr2	chemokine (C-X-C motif) receptor 2
22	12775	Ccr7	chemokine (C-C motif) receptor 7
23	12051	Bcl3	B cell leukemia/lymphoma 3
24	20292	Ccl11	chemokine (C-C motif) ligand 11
25	16199	Il9r	interleukin 9 receptor
26	20293	Ccl12	chemokine (C-C motif) ligand 12
27	20296	Ccl2	chemokine (C-C motif) ligand 2
28	17874	Myd88	myeloid differentiation primary response gene 88
29	16184	Il2ra	interleukin 2 receptor, alpha chain
30	14281	Fos	FBJ osteosarcoma oncogene
31	20848	Stat3	signal transducer and activator of transcription 3
32	16847	Lepr	leptin receptor
33	22695	Zfp36	zinc finger protein 36
34	170786	Cd209a	CD209a antigen
35	16153	Il10	interleukin 10
36	12770	Ccr1l1	chemokine (C-C motif) receptor 1-like 1
37	12769	Ccr9	chemokine (C-C motif) receptor 9
38	18595	Pdgfra	platelet derived growth factor receptor, alpha polypeptide
39	18019	Nfatc2	nuclear factor of activated T cells, cytoplasmic, calcineurin dependent 2
40	20308	Ccl9	chemokine (C-C motif) ligand 9
41	12776	Ccr8	chemokine (C-C motif) receptor 8
42	20299	Ccl22	chemokine (C-C motif) ligand 22
43	56066	Cxcl11	chemokine (C-X-C motif) ligand 11
44	16452	Jak2	Janus kinase 2
45	12804	Cntfr	ciliary neurotrophic factor receptor
46	16192	Il5ra	interleukin 5 receptor, alpha
47	16177	Il1r1	interleukin 1 receptor, type I
48	13649	Egfr	epidermal growth factor receptor
49	12773	Ccr4	chemokine (C-C motif) receptor 4
50	12983	Csf2rb	colony stimulating factor 2 receptor, beta, low-affinity (granulocyte-macrophage)
51	26416	Mapk14	mitogen-activated protein kinase 14
52	20306	Ccl7	chemokine (C-C motif) ligand 7
53	12767	Cxcr4	chemokine (C-X-C motif) receptor 4
54	14128	Fcer2a	Fc receptor, IgE, low affinity II, alpha polypeptide
55	16190	Il4ra	interleukin 4 receptor, alpha
56	19698	Relb	avian reticuloendotheliosis viral (v-rel) oncogene related B
57	12766	Cxcr3	chemokine (C-X-C motif) receptor 3

58	50931	Il27ra	interleukin 27 receptor, alpha
59	57914	Crlf2	cytokine receptor-like factor 2
60	16880	Lifr	LIF receptor alpha
61	16478	Jund	jun D proto-oncogene
62	21857	Timp1	tissue inhibitor of metalloproteinase 1
63	18033	Nfkb1	nuclear factor of kappa light polypeptide gene enhancer in B cells 1, p105
64	12774	Ccr5	chemokine (C-C motif) receptor 5
65	19697	Rela	v-rel reticuloendotheliosis viral oncogene homolog A (avian)
66	12768	Ccr1	chemokine (C-C motif) receptor 1
67	18021	Nfatc3	nuclear factor of activated T cells, cytoplasmic, calcineurin dependent 3
68	12777	Ccr10	chemokine (C-C motif) receptor 10
69	73181	Nfatc4	nuclear factor of activated T cells, cytoplasmic, calcineurin dependent 4
70	12145	Cxcr5	chemokine (C-X-C motif) receptor 5
71	29857	Mapk12	mitogen-activated protein kinase 12
72	18018	Nfatc1	nuclear factor of activated T cells, cytoplasmic, calcineurin dependent 1
73	66102	Cxcl16	chemokine (C-X-C motif) ligand 16
74	16197	Il7r	interleukin 7 receptor
75	12982	Csf2ra	colony stimulating factor 2 receptor, alpha, low-affinity (granulocyte-macrophage)
76	16195	Il6st	interleukin 6 signal transducer
77	227288	Cxcr1	chemokine (C-X-C motif) receptor 1
78	12458	Ccr6	chemokine (C-C motif) receptor 6
79	57349	Ppbp	pro-platelet basic protein
80	237310	Il22ra2	interleukin 22 receptor, alpha 2
81	17329	Cxcl9	chemokine (C-X-C motif) ligand 9
82	19094	Mapk11	mitogen-activated protein kinase 11
83	16164	Il13ra1	interleukin 13 receptor, alpha 1
84	16963	Xcl1	chemokine (C motif) ligand 1
85	16185	Il2rb	interleukin 2 receptor, beta chain
86	16165	Il13ra2	interleukin 13 receptor, alpha 2
87	16154	Il10ra	interleukin 10 receptor, alpha
88	16453	Jak3	Janus kinase 3
89	12575	Cdkn1a	cyclin-dependent kinase inhibitor 1A (P21)
90	16176	Il1b	interleukin 1 beta
91	15976	Ifnar2	interferon (alpha and beta) receptor 2
92	20305	Ccl6	chemokine (C-C motif) ligand 6
93	12475	Cd14	CD14 antigen
94	24088	Tlr2	toll-like receptor 2
95	15979	Ifngr1	interferon gamma receptor 1
96	213208	Il20rb	interleukin 20 receptor beta
97	16162	Il12rb2	interleukin 12 receptor, beta 2
98	16155	Il10rb	interleukin 10 receptor, beta
99	19106	Eif2ak2	eukaryotic translation initiation factor 2-alpha kinase 2
100	15957	Ifit1	interferon-induced protein with tetratricopeptide repeats 1
101	26420	Mapk9	mitogen-activated protein kinase 9
102	14600	Ghr	growth hormone receptor
103	16451	Jak1	Janus kinase 1
104	18034	Nfkb2	nuclear factor of kappa light polypeptide gene enhancer in B cells 2, p49/p100
105	237313	Il20ra	interleukin 20 receptor, alpha
106	14283	Fosl1	fos-like antigen 1
107	17480	Mpl	myeloproliferative leukemia virus oncogene
108	26414	Mapk10	mitogen-activated protein kinase 10
109	20304	Ccl5	chemokine (C-C motif) ligand 5
110	18596	Pdgfrb	platelet derived growth factor receptor, beta polypeptide
111	26413	Mapk1	mitogen-activated protein kinase 1
112	19116	Prlr	prolactin receptor
113	60504	Il21r	interleukin 21 receptor
114	26419	Mapk8	mitogen-activated protein kinase 8
115	27056	Irf5	interferon regulatory factor 5
116	16188	Il3ra	interleukin 3 receptor, alpha chain
117	14282	Fosb	FBJ osteosarcoma oncogene B

118	15980	Ifngr2	interferon gamma receptor 2
119	209590	Il23r	interleukin 23 receptor
120	26417	Mapk3	mitogen-activated protein kinase 3
121	246256	Fcgr4	Fc receptor, IgG, low affinity IV
122	19225	Ptgs2	prostaglandin-endoperoxide synthase 2
123	15975	Ifnar1	interferon (alpha and beta) receptor 1
124	13857	Epor	erythropoietin receptor
125	242700	Ifnlr1	interferon lambda receptor 1
126	12984	Csf2rb2	colony stimulating factor 2 receptor, beta 2, low-affinity (granulocyte-macrophage)
127	16186	Il2rg	interleukin 2 receptor, gamma chain
128	16194	Il6ra	interleukin 6 receptor, alpha
129	16161	Il12rb1	interleukin 12 receptor, beta 1
130	26415	Mapk13	mitogen-activated protein kinase 13
131	12986	Csf3r	colony stimulating factor 3 receptor (granulocyte)
132	12606	Cebpa	CCAAT/enhancer binding protein (C/EBP), alpha
133	230828	Il22ra1	interleukin 22 receptor, alpha 1
134	387139	Mir20a	microRNA 20a
135	20847	Stat2	signal transducer and activator of transcription 2
136	12702	Socs3	suppressor of cytokine signaling 3
137	55985	Cxcl13	chemokine (C-X-C motif) ligand 13
138	56619	Clec4e	C-type lectin domain family 4, member e
139	17474	Clec4d	C-type lectin domain family 4, member d
140	20295	Ccl17	chemokine (C-C motif) ligand 17
141	56221	Ccl24	chemokine (C-C motif) ligand 24
142	170779	Cd209d	CD209d antigen
143	69165	Cd209b	CD209b antigen
144	54123	Irf7	interferon regulatory factor 7
145	17858	Mx2	MX dynamin-like GTPase 2
146	26385	Grk6	G protein-coupled receptor kinase 6
147	21898	Tlr4	toll-like receptor 4
148	14677	Gnai1	guanine nucleotide binding protein (G protein), alpha inhibiting 1
149	21929	Tnfaip3	tumor necrosis factor, alpha-induced protein 3
150	56838	Ccl28	chemokine (C-C motif) ligand 28
151	20312	Cx3cl1	chemokine (C-X3-C motif) ligand 1
152	20309	Cxcl15	chemokine (C-X-C motif) ligand 15
153	14679	Gnai3	guanine nucleotide binding protein (G protein), alpha inhibiting 3
154	20375	Spi1	spleen focus forming virus (SFFV) proviral integration oncogene
155	14772	Grk4	G protein-coupled receptor kinase 4
156	12519	Cd80	CD80 antigen
157	12477	Ctla4	cytotoxic T-lymphocyte-associated protein 4
158	20290	Ccl1	chemokine (C-C motif) ligand 1
159	24013	Grk1	G protein-coupled receptor kinase 1
160	20315	Cxcl12	chemokine (C-X-C motif) ligand 12
161	13614	Edn1	endothelin 1
162	12985	Csf3	colony stimulating factor 3 (granulocyte)
163	20300	Ccl25	chemokine (C-C motif) ligand 25
164	20297	Ccl20	chemokine (C-C motif) ligand 20
165	67664	Rnf125	ring finger protein 125
166	71586	Ifih1	interferon induced with helicase C domain 1
167	14678	Gnai2	guanine nucleotide binding protein (G protein), alpha inhibiting 2
168	20301	Ccl27a	chemokine (C-C motif) ligand 27A
169	216869	Arrb2	arrestin, beta 2
170	18787	Serpine1	serine (or cysteine) peptidase inhibitor, clade E, member 1
171	18791	Plat	plasminogen activator, tissue
172	110355	Grk2	G protein-coupled receptor kinase 2
173	57266	Cxcl14	chemokine (C-X-C motif) ligand 14
174	56744	Pf4	platelet factor 4
175	109689	Arrb1	arrestin, beta 1
176	19252	Dusp1	dual specificity phosphatase 1
177	20307	Ccl8	chemokine (C-C motif) ligand 8

178	14773	Grk5	G protein-coupled receptor kinase 5
179	541307	Ccl26	chemokine (C-C motif) ligand 26
180	17087	Ly96	lymphocyte antigen 96
181	18845	Plxna2	plexin A2
182	223881	Rnd1	Rho family GTPase 1
183	320129	Grk3	G protein-coupled receptor kinase 3
184	384783	Irs2	insulin receptor substrate 2
185	66513	Tab1	TGF-beta activated kinase 1/MAP3K7 binding protein 1
186	170768	Pfkfb3	6-phosphofructo-2-kinase/fructose-2,6-biphosphatase 3
187	18126	Nos2	nitric oxide synthase 2, inducible
188	60533	Cd274	CD274 antigen
189	20849	Stat4	signal transducer and activator of transcription 4
190	19664	Rbpj	recombination signal binding protein for immunoglobulin kappa J region
191	21950	Tnfsf9	tumor necrosis factor (ligand) superfamily, member 9
192	21942	Tnfrsf9	tumor necrosis factor receptor superfamily, member 9
193	170776	Cd209c	CD209c antigen
194	19668	Rbpjl	recombination signal binding protein for immunoglobulin kappa J region-like
195	16337	Insr	insulin receptor
196	15467	Eif2ak1	eukaryotic translation initiation factor 2 alpha kinase 1
197	18779	Pla2r1	phospholipase A2 receptor 1
198	17533	Mrc1	mannose receptor, C type 1
199	26427	Creb3l1	cAMP responsive element binding protein 3-like 1
200	14127	Fcer1g	Fc receptor, IgE, high affinity I, gamma polypeptide
201	170780	Cd209e	CD209e antigen
202	231655	Oasl1	2'-5' oligoadenylate synthetase-like 1
203	107607	Nod1	nucleotide-binding oligomerization domain containing 1
204	80861	Dhx58	DEXH (Asp-Glu-X-His) box polypeptide 58
205	69142	Cd209f	CD209f antigen
206	12333	Capn1	calpain 1
207	26399	Map2k6	mitogen-activated protein kinase kinase 6
208	12334	Capn2	calpain 2
209	14463	Gata4	GATA binding protein 4
210	19204	Ptafr	platelet-activating factor receptor
211	15015	H2-Q4	histocompatibility 2, Q region locus 4
212	16180	Il1rap	interleukin 1 receptor accessory protein
213	110557	H2-Q6	histocompatibility 2, Q region locus 6
214	15170	Ptpn6	protein tyrosine phosphatase, non-receptor type 6
215	15039	H2-T22	histocompatibility 2, T region locus 22
216	11535	Adm	adrenomedullin
217	56089	Ramp3	receptor (calcitonin) activity modifying protein 3
218	54140	Avpr1a	arginine vasopressin receptor 1A
219	18798	Plcb4	phospholipase C, beta 4
220	20852	Stat6	signal transducer and activator of transcription 6
221	17534	Mrc2	mannose receptor, C type 2
222	18566	Pdcd1	programmed cell death 1
223	14674	Gna13	guanine nucleotide binding protein, alpha 13
224	11863	Arnt	aryl hydrocarbon receptor nuclear translocator
225	20846	Stat1	signal transducer and activator of transcription 1
226	70192	Cd209g	CD209g antigen
227	328572	Ep300	E1A binding protein p300
228	16391	Irf9	interferon regulatory factor 9
229	22034	Traf6	TNF receptor-associated factor 6
230	20971	Sdc4	syndecan 4
231	12389	Cav1	caveolin 1, caveolae protein
232	21825	Thbs1	thrombospondin 1
233	12914	Crebbp	CREB binding protein
234	12125	Bcl2l11	BCL2-like 11 (apoptosis facilitator)
235	84112	Sucnr1	succinate receptor 1
236	14990	H2-M2	histocompatibility 2, M region locus 2
237	12571	Cdk6	cyclin-dependent kinase 6

238	11911	Atf4	activating transcription factor 4
239	54721	Tyk2	tyrosine kinase 2
240	18024	Nfe2l2	nuclear factor, erythroid derived 2, like 2
241	20850	Stat5a	signal transducer and activator of transcription 5A
242	239081	Tlr11	toll-like receptor 11
243	15200	Hbegf	heparin-binding EGF-like growth factor
244	235611	Plxnb1	plexin B1
245	12524	Cd86	CD86 antigen
246	110696	H2-M10.3	histocompatibility 2, M region locus 10.3
247	53608	Map3k6	mitogen-activated protein kinase kinase kinase 6
248	12443	Ccnd1	cyclin D1
249	15024	H2-T10	histocompatibility 2, T region locus 10
250	56717	Mtor	mechanistic target of rapamycin kinase
251	17387	Mmp14	matrix metallopeptidase 14 (membrane-inserted)
252	13666	Eif2ak3	eukaryotic translation initiation factor 2 alpha kinase 3
253	246727	Oas3	2'-5' oligoadenylate synthetase 3
254	12826	Col4a1	collagen, type IV, alpha 1
255	224129	Adcy5	adenylate cyclase 5
256	22059	Trp53	transformation related protein 53
257	20851	Stat5b	signal transducer and activator of transcription 5B
258	27103	Eif2ak4	eukaryotic translation initiation factor 2 alpha kinase 4
259	15018	H2-Q7	histocompatibility 2, Q region locus 7
260	16369	Irs3	insulin receptor substrate 3
261	16341	Eif3e	eukaryotic translation initiation factor 3, subunit E
262	231991	Creb5	cAMP responsive element binding protein 5
263	58203	Zbp1	Z-DNA binding protein 1
264	16370	Irs4	insulin receptor substrate 4
265	17126	Smad2	SMAD family member 2
266	13617	Ednra	endothelin receptor type A
267	11651	Akt1	thymoma viral proto-oncogene 1
268	333715	H2-M10.2	histocompatibility 2, M region locus 10.2
269	15042	H2-T24	histocompatibility 2, T region locus 24
270	21402	Skp1a	S-phase kinase-associated protein 1A
271	26408	Map3k5	mitogen-activated protein kinase kinase kinase 5
272	667977	Gm8909	predicted gene 8909
273	15214	Hey2	hairy/enhancer-of-split related with YRPW motif 2
274	15205	Hes1	hes family bHLH transcription factor 1
275	15208	Hes5	hes family bHLH transcription factor 5
276	23797	Akt3	thymoma viral proto-oncogene 3
277	16367	Irs1	insulin receptor substrate 1
278	16409	Itgam	integrin alpha M
279	69717	Gm10499	predicted gene 10499
280	667803	H2-T-ps	histocompatibility 2, T region locus, pseudogene
281	15040	H2-T23	histocompatibility 2, T region locus 23
282	20787	Srebf1	sterol regulatory element binding transcription factor 1
283	56637	Gsk3b	glycogen synthase kinase 3 beta
284	12444	Ccnd2	cyclin D2
285	208647	Creb3l2	cAMP responsive element binding protein 3-like 2
286	83383	Tfap4	transcription factor AP4
287	20963	Syk	spleen tyrosine kinase
288	17128	Smad4	SMAD family member 4
289	26965	Cul1	cullin 1
290	14129	Fcgr1	Fc receptor, IgG, high affinity I
291	11652	Akt2	thymoma viral proto-oncogene 2
292	12428	Ccna2	cyclin A2
293	14985	H2-M10.1	histocompatibility 2, M region locus 10.1
294	15043	H2-T3	histocompatibility 2, T region locus 3
295	15213	Hey1	hairy/enhancer-of-split related with YRPW motif 1
296	12367	Casp3	caspase 3
297	15013	H2-Q2	histocompatibility 2, Q region locus 2
298	14964	H2-D1	histocompatibility 2, D region locus 1

299	11909	Atf2	activating transcription factor 2
300	27401	Skp2	S-phase kinase-associated protein 2 (p45)
301	246728	Oas2	2'-5' oligoadenylate synthetase 2
302	17127	Smad3	SMAD family member 3
303	16402	Itga5	integrin alpha 5 (fibronectin receptor alpha)
304	12913	Creb3	cAMP responsive element binding protein 3
305	50723	Icosl	icos ligand
306	74747	Ddit4	DNA-damage-inducible transcript 4
307	12445	Ccnd3	cyclin D3
308	56198	Heyl	hairy/enhancer-of-split related with YRPW motif-like
309	208677	Creb3l3	cAMP responsive element binding protein 3-like 3
310	18796	Plcb2	phospholipase C, beta 2
311	224754	H2-M11	histocompatibility 2, M region locus 11
312	243270	Hcar1	hydrocarboxylic acid receptor 1
313	224753	H2-M10.4	histocompatibility 2, M region locus 10.4
314	224756	H2-M1	histocompatibility 2, M region locus 1
315	14972	H2-K1	histocompatibility 2, K1, K region
316	224761	H2-M10.5	histocompatibility 2, M region locus 10.5
317	12912	Creb1	cAMP responsive element binding protein 1
318	240095	H2-M5	histocompatibility 2, M region locus 5
319	78284	Creb3l4	cAMP responsive element binding protein 3-like 4
320	12566	Cdk2	cyclin-dependent kinase 2
321	15006	H2-Q1	histocompatibility 2, Q region locus 1
322	329934	Foxo6	forkhead box O6
323	54601	Foxo4	forkhead box O4
324	15007	H2-Q10	histocompatibility 2, Q region locus 10
325	14963	H2-Bl	histocompatibility 2, blastocyst
326	26409	Map3k7	mitogen-activated protein kinase kinase kinase 7
327	12447	Ccne1	cyclin E1
328	12427	Ccna1	cyclin A1
329	12827	Col4a2	collagen, type IV, alpha 2
330	56458	Foxo1	forkhead box O1
331	12915	Atf6b	activating transcription factor 6 beta
332	14991	H2-M3	histocompatibility 2, M region locus 3
333	56484	Foxo3	forkhead box O3
334	14997	H2-M9	histocompatibility 2, M region locus 9
335	12567	Cdk4	cyclin-dependent kinase 4
336	399549	H2-M10.6	histocompatibility 2, M region locus 10.6
337	56438	Rbx1	ring-box 1
338	12448	Ccne2	cyclin E2
339	723925	Mir106b	microRNA 106b
340	17395	Mmp9	matrix metallopeptidase 9
341	12562	Cdh5	cadherin 5
342	15370	Nr4a1	nuclear receptor subfamily 4, group A, member 1
343	20845	Star	steroidogenic acute regulatory protein
344	56532	Ripk3	receptor-interacting serine-threonine kinase 3
345	14676	Gna15	guanine nucleotide binding protein, alpha 15
346	14131	Fcgr3	Fc receptor, IgG, low affinity III
347	12390	Cav2	caveolin 2
348	13618	Ednrb	endothelin receptor type B
349	12946	Cr1l	complement component (3b/4b) receptor 1-like
350	387194	Mir199a-1	microRNA 199a-1
351	16414	Itgb2	integrin beta 2
352	14102	Fas	Fas (TNF receptor superfamily member 6)
353	16415	Itgb2l	integrin beta 2-like
354	23939	Mapk7	mitogen-activated protein kinase 7
355	17254	Slc3a2	solute carrier family 3 (activators of dibasic and neutral amino acid transport), member 2
356	26570	Slc7a11	solute carrier family 7 (cationic amino acid transporter, y+ system), member 11
357	67653	4930544G11Rik	RIKEN cDNA 4930544G11 gene
358	140571	Plxnb3	plexin B3

359	112406	Egln2	egl-9 family hypoxia-inducible factor 2
360	16421	Itgb7	integrin beta 7
361	22337	Vdr	vitamin D (1,25-dihydroxyvitamin D3) receptor
362	18844	Plxna1	plexin A1
363	192656	Ripk2	receptor (TNFRSF)-interacting serine-threonine kinase 2
364	18797	Plcb3	phospholipase C, beta 3
365	11854	Rhod	ras homolog family member D
366	12393	Runx2	runt related transcription factor 2
367	17386	Mmp13	matrix metallopeptidase 13
368	235320	Zbtb16	zinc finger and BTB domain containing 16
369	16480	Jup	junction plakoglobin
370	18846	Plxna3	plexin A3
371	243743	Plxna4	plexin A4
372	140570	Plxnb2	plexin B2
373	12061	Bdkrb1	bradykinin receptor, beta 1
374	242316	Gdf6	growth differentiation factor 6
375	12168	Bmpr2	bone morphogenetic protein receptor, type II (serine/threonine kinase)
376	26406	Map3k3	mitogen-activated protein kinase kinase kinase 3
377	100038947	Sirpb1c	signal-regulatory protein beta 1C
378	18127	Nos3	nitric oxide synthase 3, endothelial cell
379	17869	Myc	myelocytomatosis oncogene
380	53945	Slc40a1	solute carrier family 40 (iron-regulated transporter), member 1
381	15203	Heph	hephaestin
382	13163	Daxx	Fas death domain-associated protein
383	20353	Sema4c	sema domain, immunoglobulin domain (Ig), transmembrane domain (TM) and short cytoplasmic domain, (semaphorin) 4C
384	11848	Rhoa	ras homolog family member A
385	14939	Gzmb	granzyme B
386	13347	Dffa	DNA fragmentation factor, alpha subunit
387	17164	Mapkapk2	MAP kinase-activated protein kinase 2
388	18795	Plcb1	phospholipase C, beta 1
389	17390	Mmp2	matrix metallopeptidase 2
390	16906	Lmnb1	lamin B1
391	17210	Mcl1	myeloid cell leukemia sequence 1
392	14728	Lilrb4a	leukocyte immunoglobulin-like receptor, subfamily B, member 4A
393	230073	Ddx58	DEAD (Asp-Glu-Ala-Asp) box polypeptide 58
394	14061	F2	coagulation factor II
395	16000	Igf1	insulin-like growth factor 1
396	12795	Plk3	polo like kinase 3
397	216799	Nlrp3	NLR family, pyrin domain containing 3
398	54131	Irf3	interferon regulatory factor 3
399	14672	Gna11	guanine nucleotide binding protein, alpha 11
400	11998	Avp	arginine vasopressin
401	108099	Prkag2	protein kinase, AMP-activated, gamma 2 non-catalytic subunit
402	14675	Gna14	guanine nucleotide binding protein, alpha 14
403	19416	Rasd1	RAS, dexamethasone-induced 1
404	14701	Gng12	guanine nucleotide binding protein (G protein), gamma 12
405	54393	Gabbr1	gamma-aminobutyric acid (GABA) B receptor, 1
406	67405	Nts	neurotensin
407	18216	Ntsr1	neurotensin receptor 1
408	16173	Il18	interleukin 18
409	12608	Cebpb	CCAAT/enhancer binding protein (C/EBP), beta
410	12265	Ciita	class II transactivator
411	19016	Pparg	peroxisome proliferator activated receptor gamma
412	11770	Fabp4	fatty acid binding protein 4, adipocyte
413	16878	Lif	leukemia inhibitory factor
414	11545	Parp1	poly (ADP-ribose) polymerase family, member 1
415	18747	Prkaca	protein kinase, cAMP dependent, catalytic, alpha
416	104099	Itga9	integrin alpha 9
417	14683	Gnas	GNAS (guanine nucleotide binding protein, alpha stimulating) complex locus
418	16324	Inhbb	inhibin beta-B

419	11479	Acvr1b	activin A receptor, type 1B
420	18788	Serpinb2	serine (or cysteine) peptidase inhibitor, clade B, member 2
421	85031	Pla1a	phospholipase A1 member A
422	11606	Agt	angiotensinogen (serpin peptidase inhibitor, clade A, member 8)
423	14673	Gna12	guanine nucleotide binding protein, alpha 12
424	14682	Gnaq	guanine nucleotide binding protein, alpha q polypeptide
425	109648	Npy	neuropeptide Y
426	16419	Itgb5	integrin beta 5
427	16776	Lama5	laminin, alpha 5
428	381290	Atp2b4	ATPase, Ca++ transporting, plasma membrane 4
429	81877	Tnxb	tenascin XB
430	22147	Tuba3b	tubulin, alpha 3B
431	11555	Adrb2	adrenergic receptor, beta 2
432	116903	Calcb	calcitonin-related polypeptide, beta
433	12370	Casp8	caspase 8
434	22144	Tuba3a	tubulin, alpha 3A
435	80885	Hcar2	hydroxycarboxylic acid receptor 2
436	238463	Tubal3	tubulin, alpha-like 3
437	12122	Bid	BH3 interacting domain death agonist
438	16706	Ksr1	kinase suppressor of ras 1
439	328417	Parp4	poly (ADP-ribose) polymerase family, member 4
440	12310	Calca	calcitonin/calcitonin-related polypeptide, alpha
441	17389	Mmp16	matrix metallopeptidase 16
442	233079	Ffar2	free fatty acid receptor 2
443	11480	Acvr2a	activin receptor IIA
444	21923	Tnc	tenascin C
445	22145	Tuba4a	tubulin, alpha 4A
446	12669	Chrm1	cholinergic receptor, muscarinic 1, CNS
447	12369	Casp7	caspase 7
448	15894	Icam1	intercellular adhesion molecule 1
449	18430	Oxtr	oxytocin receptor
450	12512	Cd63	CD63 antigen
451	18217	Ntsr2	neurotensin receptor 2
452	17086	Ncr1	natural cytotoxicity triggering receptor 1
453	20620	Plk2	polo like kinase 2
454	16907	Lmnb2	lamin B2
455	208188	Ghsr	growth hormone secretagogue receptor
456	20606	Sstr2	somatostatin receptor 2
457	22146	Tuba1c	tubulin, alpha 1C
458	18712	Pim1	proviral integration site 1
459	22339	Vegfa	vascular endothelial growth factor A
460	22142	Tuba1a	tubulin, alpha 1A
461	18952	4-Sep	septin 4
462	11797	Birc2	baculoviral IAP repeat-containing 2
463	58185	Rsad2	radical S-adenosyl methionine domain containing 2
464	110196	Fdps	farnesyl diphosphate synthetase
465	16905	Lmna	lamin A
466	70686	Dusp16	dual specificity phosphatase 16
467	11546	Parp2	poly (ADP-ribose) polymerase family, member 2
468	53857	Tuba8	tubulin, alpha 8
469	235587	Parp3	poly (ADP-ribose) polymerase family, member 3
470	12048	Bcl2l1	BCL2-like 1
471	18166	Npy1r	neuropeptide Y receptor Y1
472	15552	Htr1d	5-hydroxytryptamine (serotonin) receptor 1D
473	15550	Htr1a	5-hydroxytryptamine (serotonin) receptor 1A
474	22640	Zfp1	zinc finger protein 1
475	11539	Adora1	adenosine A1 receptor
476	243764	Chrm2	cholinergic receptor, muscarinic 2, cardiac
477	14284	Fosl2	fos-like antigen 2
478	13688	Eif4ebp2	eukaryotic translation initiation factor 4E binding protein 2
479	20656	Sod2	superoxide dismutase 2, mitochondrial

480	69632	Arhgef12	Rho guanine nucleotide exchange factor (GEF) 12
481	15551	Htr1b	5-hydroxytryptamine (serotonin) receptor 1B
482	18143	Npas2	neuronal PAS domain protein 2
483	18626	Per1	period circadian clock 1
484	20609	Sstr5	somatostatin receptor 5
485	14709	Gng8	guanine nucleotide binding protein (G protein), gamma 8
486	12290	Cacna1e	calcium channel, voltage-dependent, R type, alpha 1E subunit
487	19395	Rasgrp2	RAS, guanyl releasing protein 2
488	246730	Oas1a	2'-5' oligoadenylate synthetase 1A
489	242425	Gabbr2	gamma-aminobutyric acid (GABA) B receptor, 2
490	19218	Ptger3	prostaglandin E receptor 3 (subtype EP3)
491	15557	Htr1f	5-hydroxytryptamine (serotonin) receptor 1F
492	13489	Drd2	dopamine receptor D2
493	12842	Col1a1	collagen, type I, alpha 1
494	20339	Sele	selectin, endothelial cell
495	11601	Angpt2	angiopoietin 2
496	14257	Flt4	FMS-like tyrosine kinase 4
497	12062	Bdkrb2	bradykinin receptor, beta 2
498	12028	Bax	BCL2-associated X protein
499	16196	Il7	interleukin 7
500	20605	Sstr1	somatostatin receptor 1
501	241113	Prkag3	protein kinase, AMP-activated, gamma 3 non-catalytic subunit
502	239556	Cacna1i	calcium channel, voltage-dependent, alpha 1I subunit
503	105787	Prkaa1	protein kinase, AMP-activated, alpha 1 catalytic subunit
504	15234	Hgf	hepatocyte growth factor
505	13537	Dusp2	dual specificity phosphatase 2
506	12703	Socs1	suppressor of cytokine signaling 1
507	20346	Sema3a	sema domain, immunoglobulin domain (Ig), short basic domain, secreted, (semaphorin) 3A
508	18186	Nrp1	neuropilin 1
509	13616	Edn3	endothelin 3
510	14707	Gng5	guanine nucleotide binding protein (G protein), gamma 5
511	15376	Foxa2	forkhead box A2
512	226421	Rab7b	RAB7B, member RAS oncogene family
513	67951	Tubb6	tubulin, beta 6 class V
514	16160	Il12b	interleukin 12b
515	19079	Prkab1	protein kinase, AMP-activated, beta 1 non-catalytic subunit
516	241226	Itga8	integrin alpha 8
517	232801	Lilra5	leukocyte immunoglobulin-like receptor, subfamily A (with TM domain), member 5
518	15944	Irgm1	immunity-related GTPase family M member 1
519	12166	Bmpr1a	bone morphogenetic protein receptor, type 1A
520	14696	Gnb4	guanine nucleotide binding protein (G protein), beta 4
521	18106	Cd244a	CD244 molecule A
522	26904	Sh2d1b1	SH2 domain containing 1B1
523	18125	Nos1	nitric oxide synthase 1, neuronal
524	16643	Klrd1	killer cell lectin-like receptor, subfamily D, member 1
525	16598	Klf2	Kruppel-like factor 2 (lung)
526	19017	Ppargc1a	peroxisome proliferative activated receptor, gamma, coactivator 1 alpha
527	108079	Prkaa2	protein kinase, AMP-activated, alpha 2 catalytic subunit
528	74568	Mlkl	mixed lineage kinase domain-like
529	14186	Fgfr4	fibroblast growth factor receptor 4
530	56480	Tbk1	TANK-binding kinase 1
531	13039	Ctsl	cathepsin L
532	16985	Lsp1	lymphocyte specific 1
533	23962	Oasl2	2'-5' oligoadenylate synthetase-like 2
534	23960	Oas1g	2'-5' oligoadenylate synthetase 1G
535	14699	Gngt1	guanine nucleotide binding protein (G protein), gamma transducing activity polypeptide 1
536	20698	Sphk1	sphingosine kinase 1
537	21687	Tek	TEK receptor tyrosine kinase
538	19082	Prkag1	protein kinase, AMP-activated, gamma 1 non-catalytic subunit

539	14693	Gnb2	guanine nucleotide binding protein (G protein), beta 2
540	108097	Prkab2	protein kinase, AMP-activated, beta 2 non-catalytic subunit
541	53314	Batf	basic leucine zipper transcription factor, ATF-like
542	14704	Gng3	guanine nucleotide binding protein (G protein), gamma 3
543	103583	Fbxw11	F-box and WD-40 domain protein 11
544	21809	Tgfb3	transforming growth factor, beta 3
545	26397	Map2k3	mitogen-activated protein kinase kinase 3
546	19124	Procr	protein C receptor, endothelial
547	21824	Thbd	thrombomodulin
548	66066	Gng11	guanine nucleotide binding protein (G protein), gamma 11
549	11520	Plin2	perilipin 2
550	240047	Mmp25	matrix metallopeptidase 25
551	14702	Gng2	guanine nucleotide binding protein (G protein), gamma 2
552	14708	Gng7	guanine nucleotide binding protein (G protein), gamma 7
553	57875	Angptl4	angiopoietin-like 4
554	75608	Chmp4b	charged multivesicular body protein 4B
555	545486	Tubb1	tubulin, beta 1 class VI
556	14933	Gk	glycerol kinase
557	15951	Ifi204	interferon activated gene 204
558	66824	Pycard	PYD and CARD domain containing
559	64337	Gng13	guanine nucleotide binding protein (G protein), gamma 13
560	208650	Cblb	Casitas B-lineage lymphoma b
561	21803	Tgfb1	transforming growth factor, beta 1
562	21808	Tgfb2	transforming growth factor, beta 2
563	226519	Lamc1	laminin, gamma 1
564	14706	Gng4	guanine nucleotide binding protein (G protein), gamma 4
565	70729	Nos1ap	nitric oxide synthase 1 (neuronal) adaptor protein
566	18805	Pld1	phospholipase D1
567	14695	Gnb3	guanine nucleotide binding protein (G protein), beta 3
568	117149	Tirap	toll-interleukin 1 receptor (TIR) domain-containing adaptor protein
569	11481	Acvr2b	activin receptor IIB
570	14700	Gng10	guanine nucleotide binding protein (G protein), gamma 10
571	14688	Gnb1	guanine nucleotide binding protein (G protein), beta 1
572	14697	Gnb5	guanine nucleotide binding protein (G protein), beta 5
573	50790	Acsl4	acyl-CoA synthetase long-chain family member 4
574	14681	Gnao1	guanine nucleotide binding protein, alpha O
575	66724	Tab3	TGF-beta activated kinase 1/MAP3K7 binding protein 3
576	18815	Plg	plasminogen
577	12505	Cd44	CD44 antigen
578	15519	Hsp90aa1	heat shock protein 90, alpha (cytosolic), class A member 1
579	286940	Flnb	filamin, beta
580	14710	Gngt2	guanine nucleotide binding protein (G protein), gamma transducing activity polypeptide 2
581	12315	Calm3	calmodulin 3
582	18749	Prkacb	protein kinase, cAMP dependent, catalytic, beta
583	18854	Pml	promyelocytic leukemia
584	18119	Nodal	nodal
585	66911	Nudt16l1	nudix (nucleoside diphosphate linked moiety X)-type motif 16-like 1
586	21826	Thbs2	thrombospondin 2
587	20662	Sos1	SOS Ras/Rac guanine nucleotide exchange factor 1
588	12167	Bmpr1b	bone morphogenetic protein receptor, type 1B
589	494124	Calm5	calmodulin 5
590	16456	F11r	F11 receptor
591	59027	Nampt	nicotinamide phosphoribosyltransferase
592	13058	Cybb	cytochrome b-245, beta polypeptide
593	18792	Plau	plasminogen activator, urokinase
594	18793	Plaur	plasminogen activator, urokinase receptor
595	110891	Slc8a2	solute carrier family 8 (sodium/calcium exchanger), member 2
596	26410	Map3k8	mitogen-activated protein kinase kinase kinase 8
597	26398	Map2k4	mitogen-activated protein kinase kinase 4
598	56489	Ikbke	inhibitor of kappaB kinase epsilon

599	17000	Ltbr	lymphotoxin B receptor
600	14083	Ptk2	PTK2 protein tyrosine kinase 2
601	80859	Nfkbiz	nuclear factor of kappa light polypeptide gene enhancer in B cells inhibitor, zeta
602	17873	Gadd45b	growth arrest and DNA-damage-inducible 45 beta
603	26407	Map3k4	mitogen-activated protein kinase kinase kinase 4
604	16416	Itgb3	integrin beta 3
605	11477	Acvr1	activin A receptor, type 1
606	269275	Acvr1c	activin A receptor, type IC
607	75600	Calml4	calmodulin-like 4
608	13660	Ehd1	EH-domain containing 1
609	75767	Rab11fip1	RAB11 family interacting protein 1 (class I)
610	15277	Hk2	hexokinase 2
611	12824	Col2a1	collagen, type II, alpha 1
612	16145	Igtp	interferon gamma induced GTPase
613	80796	Calm4	calmodulin 4
614	18750	Prkca	protein kinase C, alpha
615	14313	Fst	follistatin
616	76373	Zfp773	zinc finger protein 773
617	240672	Dusp5	dual specificity phosphatase 5
618	22042	Tfrc	transferrin receptor
619	12833	Col6a1	collagen, type VI, alpha 1
620	11676	Aldoc	aldolase C, fructose-bisphosphate
621	94216	Col4a6	collagen, type IV, alpha 6
622	12845	Comp	cartilage oligomeric matrix protein
623	15194	Htt	huntingtin
624	16513	Kcnj10	potassium inwardly-rectifying channel, subfamily J, member 10
625	19303	Pxn	paxillin
626	12479	Cd1d1	CD1d1 antigen
627	217069	Trim25	tripartite motif-containing 25
628	56421	Pfkp	phosphofructokinase, platelet
629	13038	Ctsk	cathepsin K
630	16179	Irak1	interleukin-1 receptor-associated kinase 1
631	19766	Ripk1	receptor (TNFRSF)-interacting serine-threonine kinase 1
632	52118	Pvr	poliovirus receptor
633	100043314	Tigit	T cell immunoreceptor with Ig and ITIM domains
634	18514	Pbx1	pre B cell leukemia homeobox 1
635	228607	Mavs	mitochondrial antiviral signaling protein
636	12700	Cish	cytokine inducible SH2-containing protein
637	12828	Col4a3	collagen, type IV, alpha 3
638	12830	Col4a5	collagen, type IV, alpha 5
639	14268	Fn1	fibronectin 1
640	216233	Socs2	suppressor of cytokine signaling 2
641	21827	Thbs3	thrombospondin 3
642	67972	Atp2b1	ATPase, Ca++ transporting, plasma membrane 1
643	12840	Col9a2	collagen, type IX, alpha 2
644	381810	Lpar5	lysophosphatidic acid receptor 5
645	320207	Pik3r5	phosphoinositide-3-kinase regulatory subunit 5
646	21960	Tnr	tenascin R
647	268902	Robo2	roundabout guidance receptor 2
648	259302	Srgap3	SLIT-ROBO Rho GTPase activating protein 3

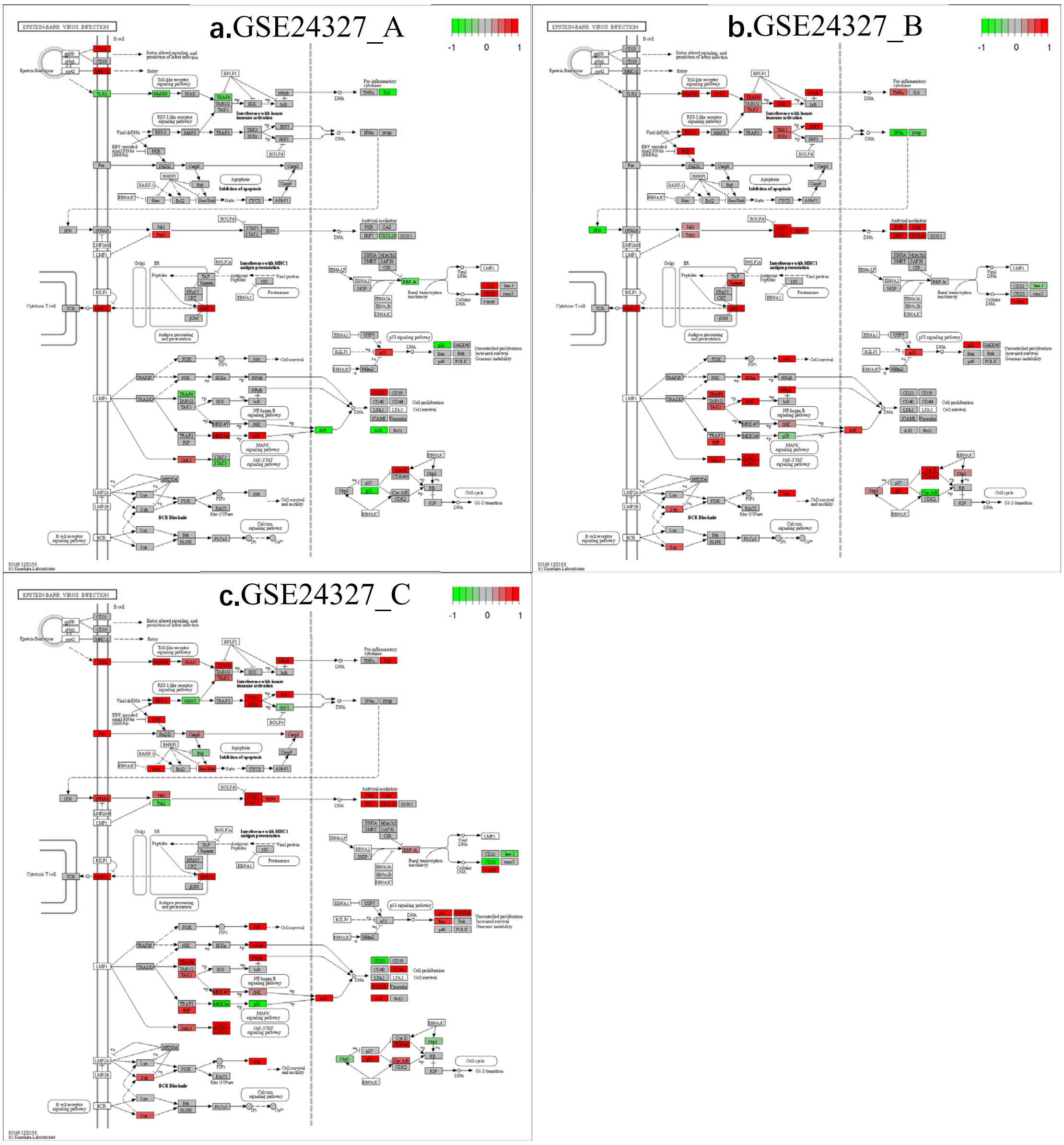

Figure S1 | Comparison on the rendered Epstein-Barr virus infection signaling pathway (mmu05169)

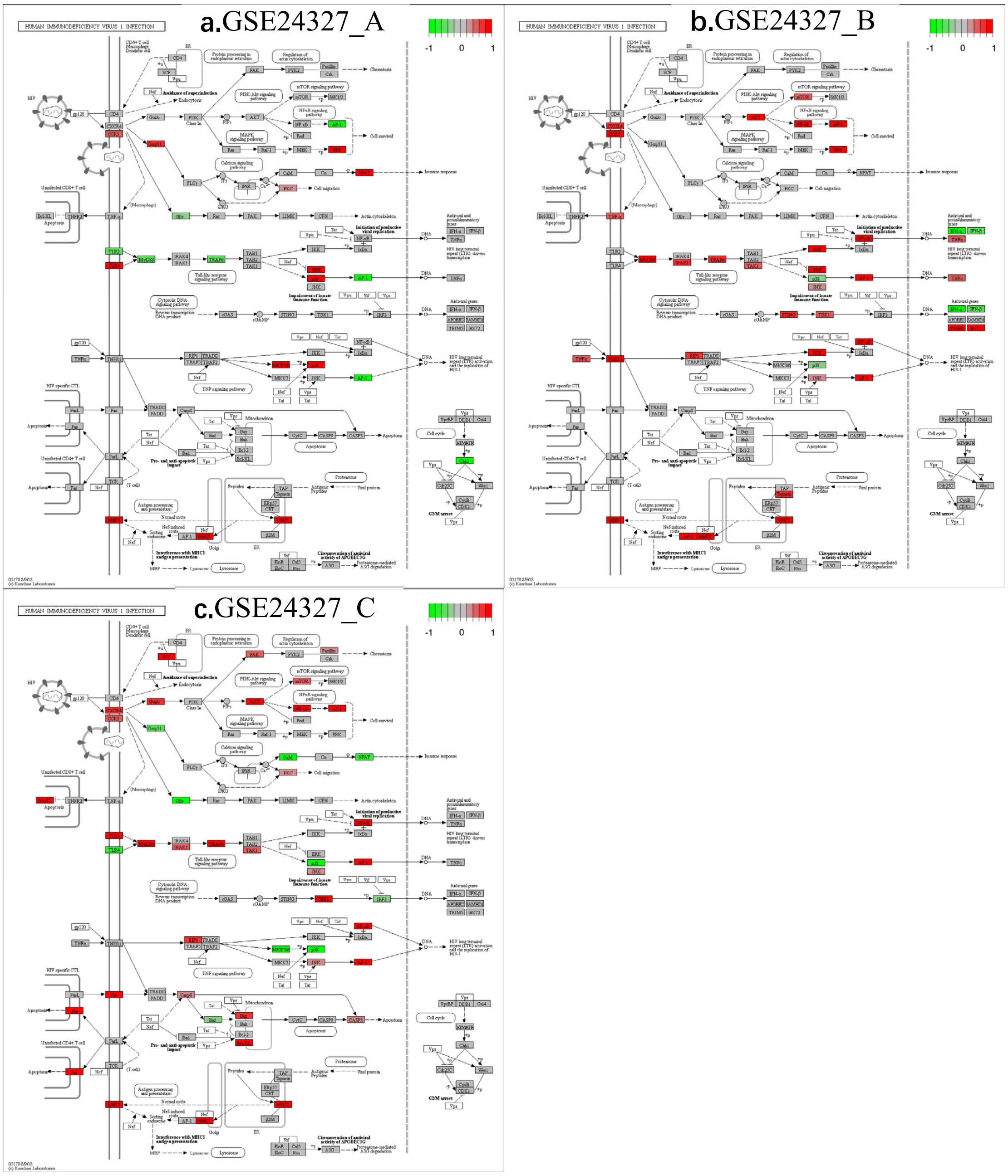

Figure S2 | Comparison on the rendered Human immunodeficiency virus 1 infection signaling pathway (mmu05170)

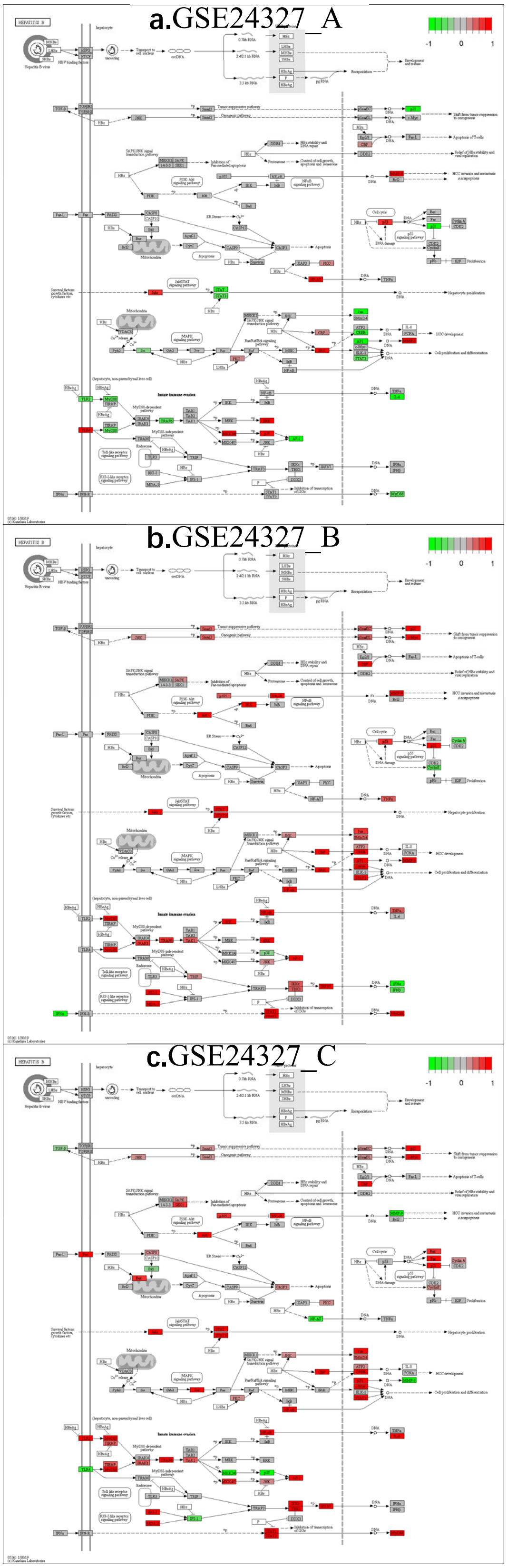

Figure S3 | Comparison on the rendered Hepatitis B signaling pathway (mmu05161)

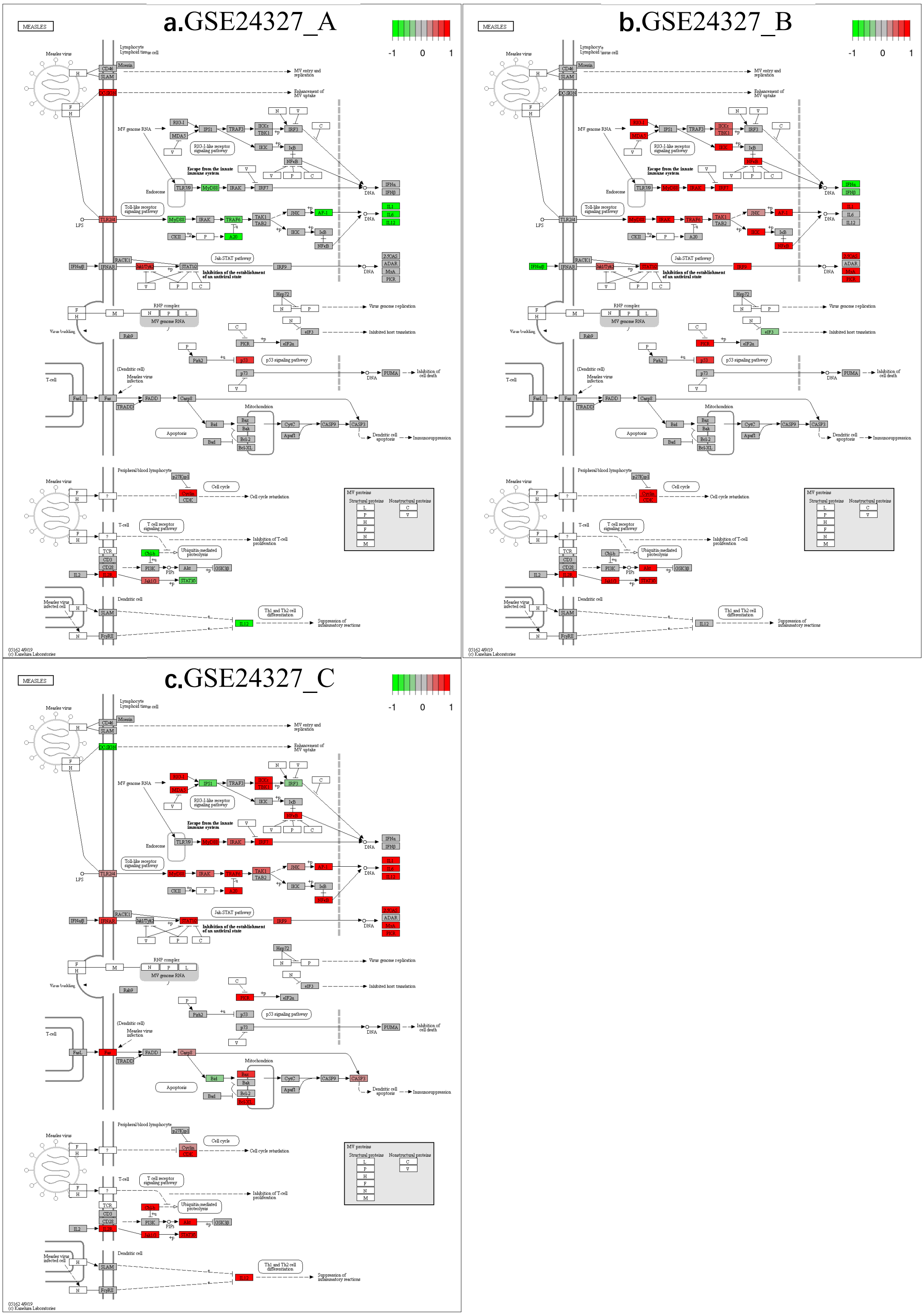

Figure S4 | Comparison on the rendered Measles signaling pathway (mmu05162)

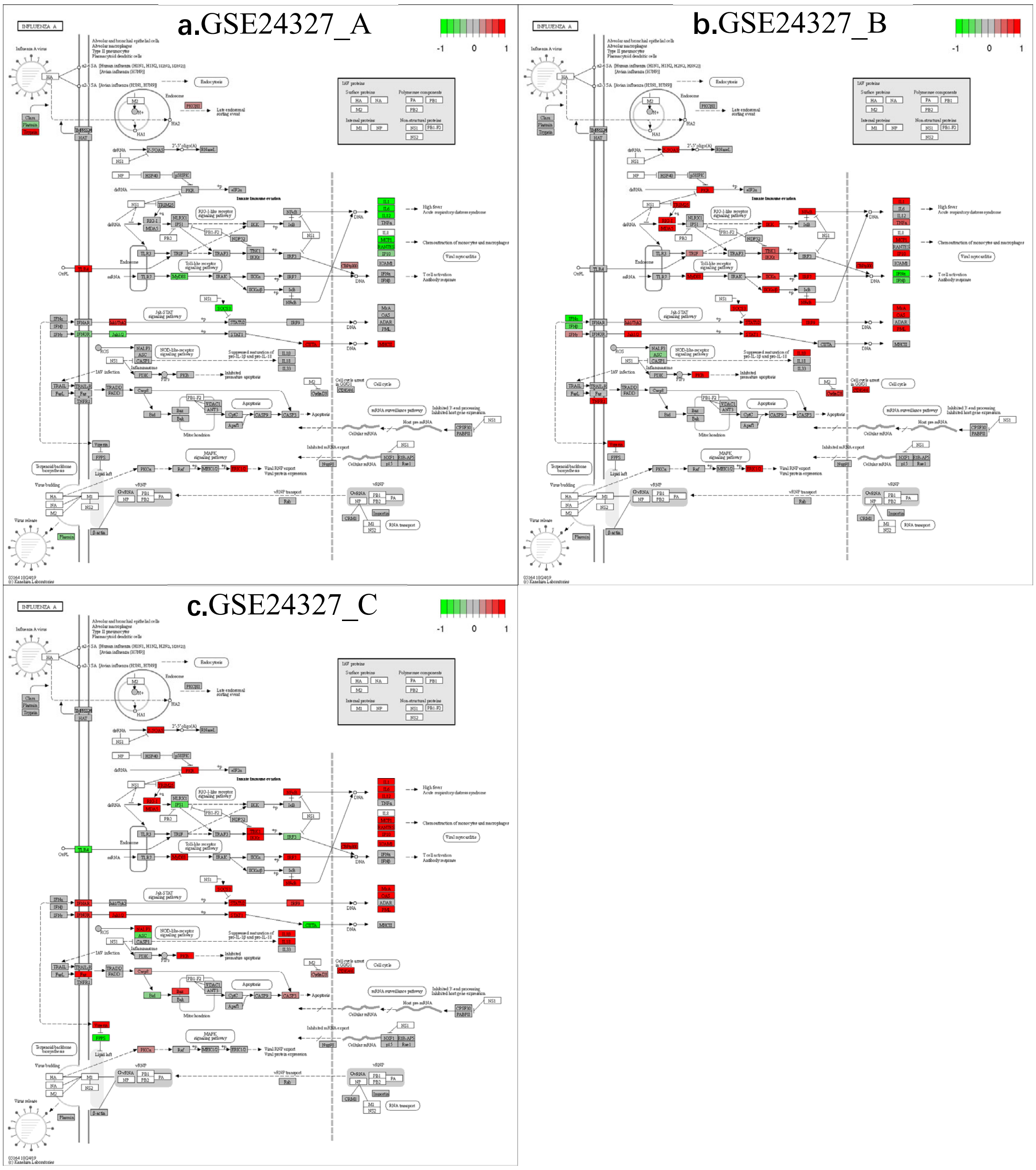

Figure S5 | Comparison on the rendered Influenza A signaling pathway (mmu05164)

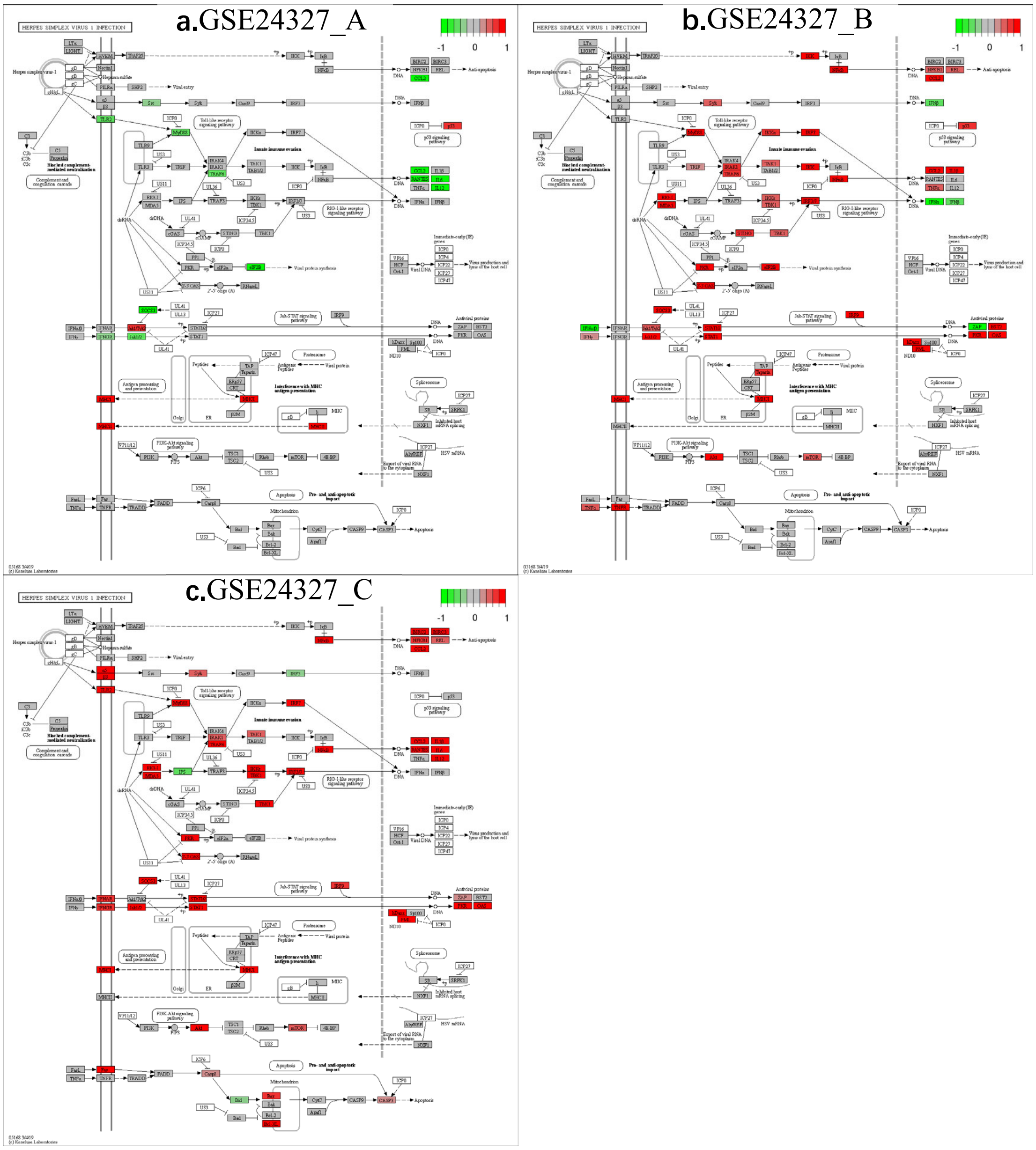

Figure S6 | Comparison on the rendered Herpes simplex virus 1 infection signaling pathway (mmu05168)

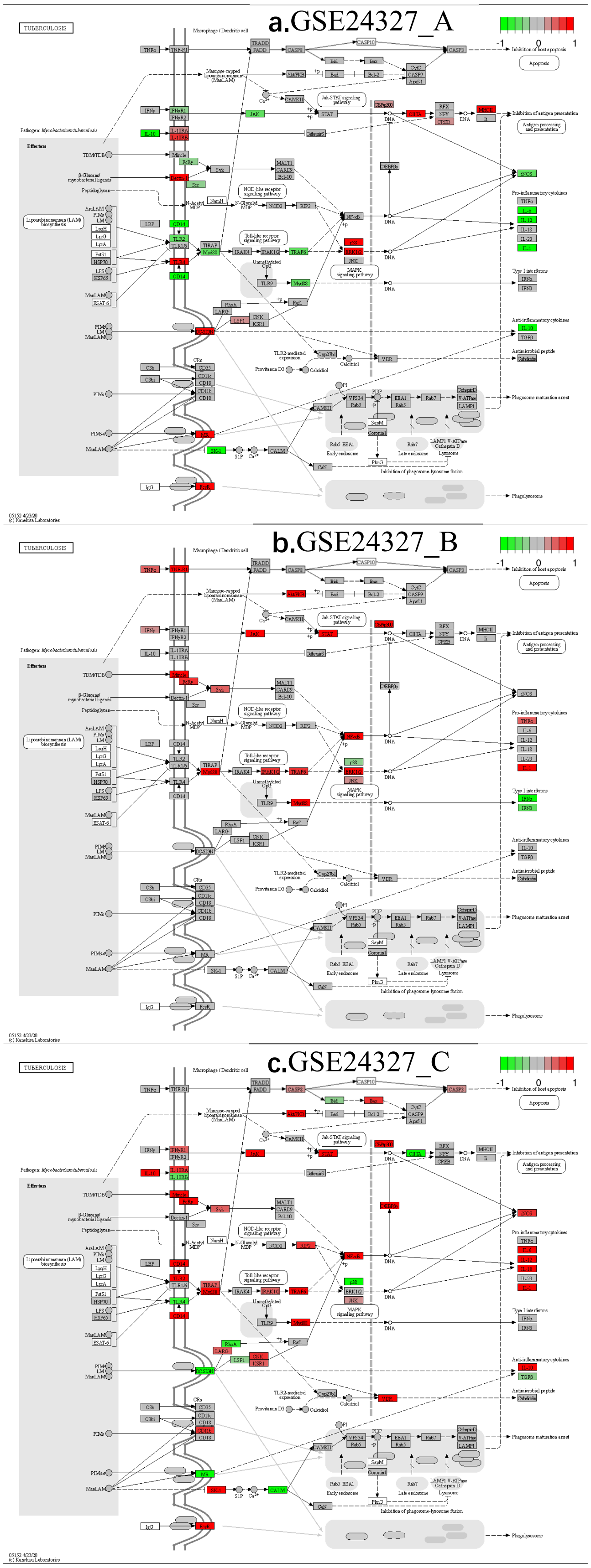

Figure S7 | Comparison on the rendered Tuberculosis signaling pathway (mmu05152)

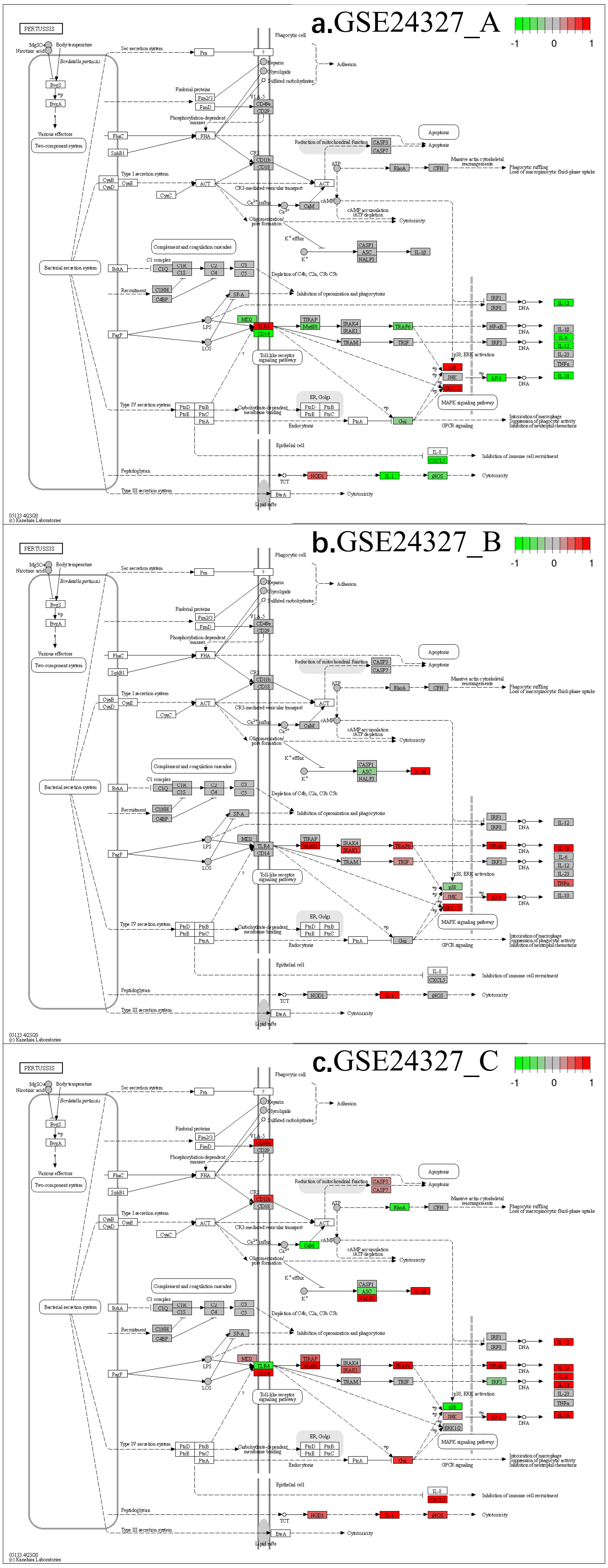

Figure S8 | Comparison on the rendered Pertussis signaling pathway (mmu05133).

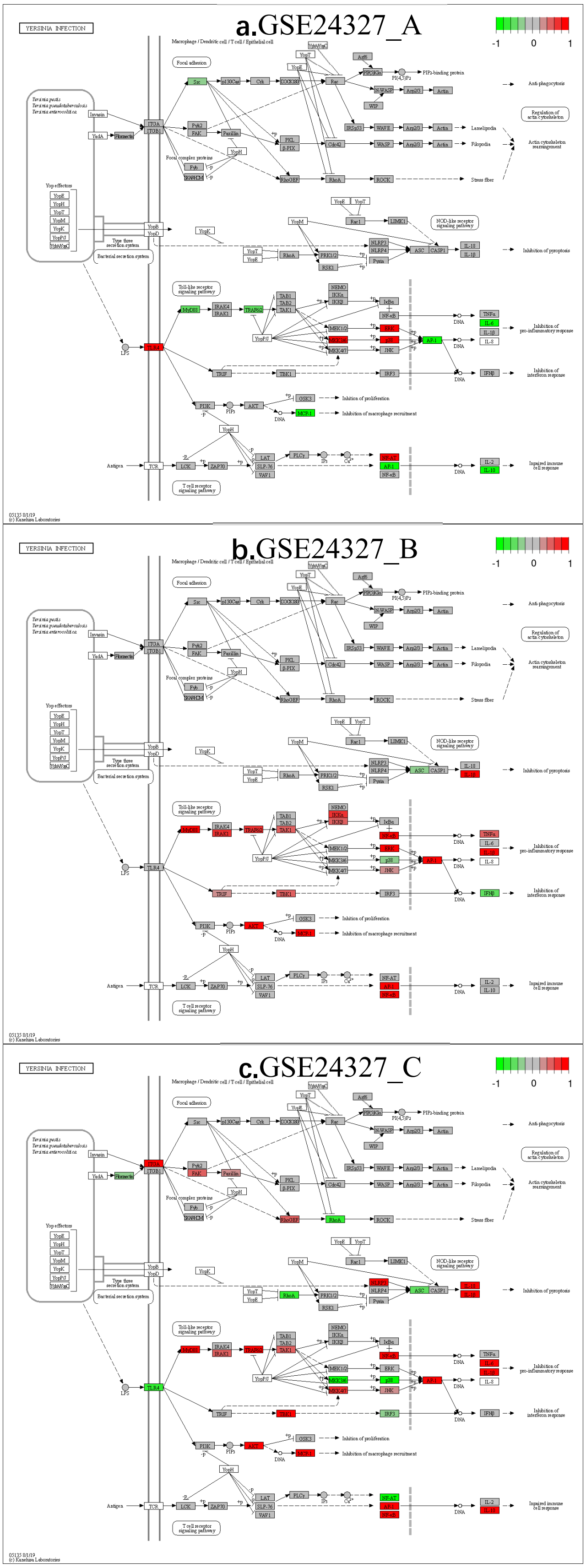

Figure S9 | Comparison on the rendered Yersinia infection signaling pathway (mmu05135).

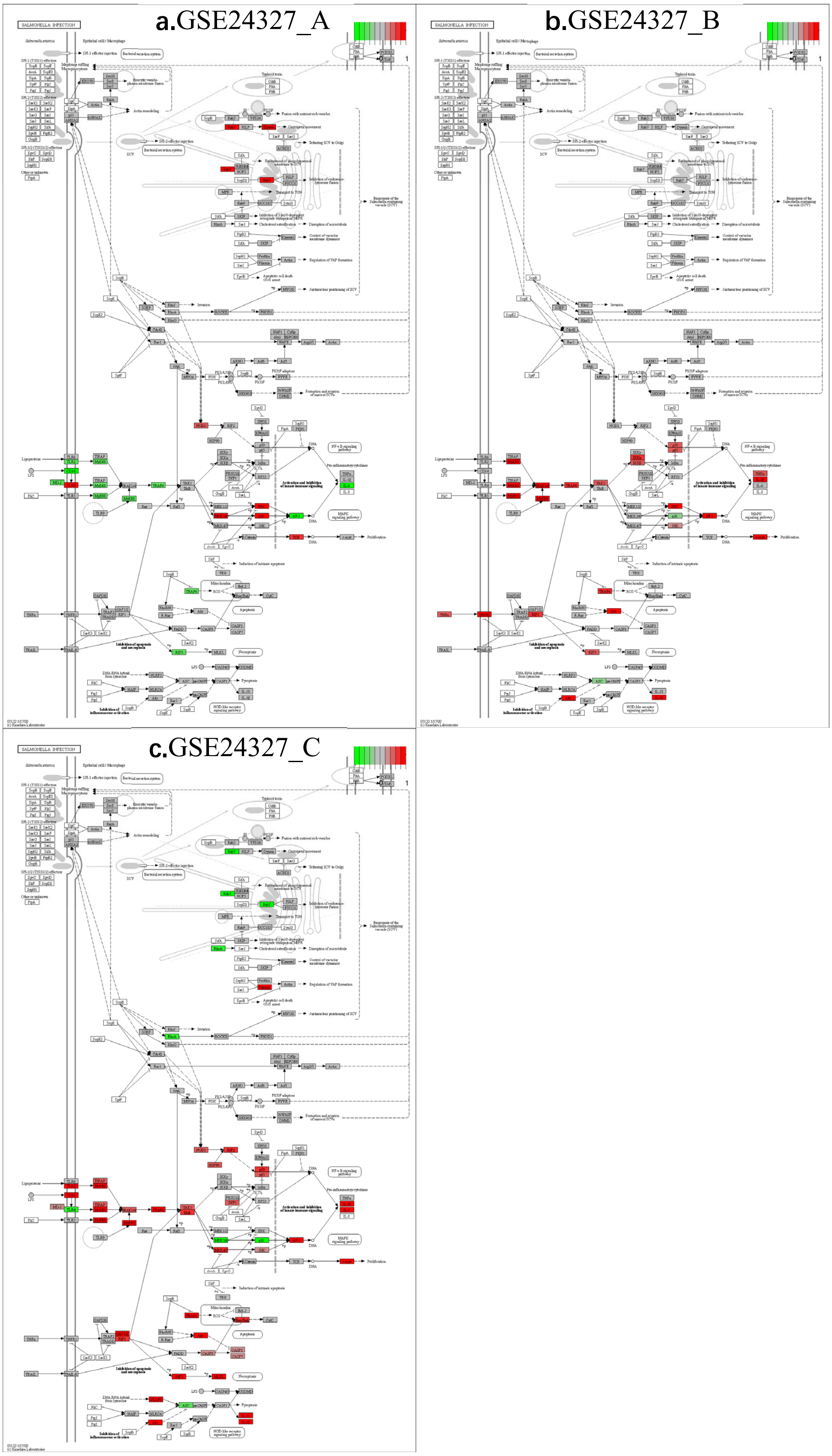

Figure S10 | Comparison on the rendered Salmonella infection signaling pathway (mmu05132).

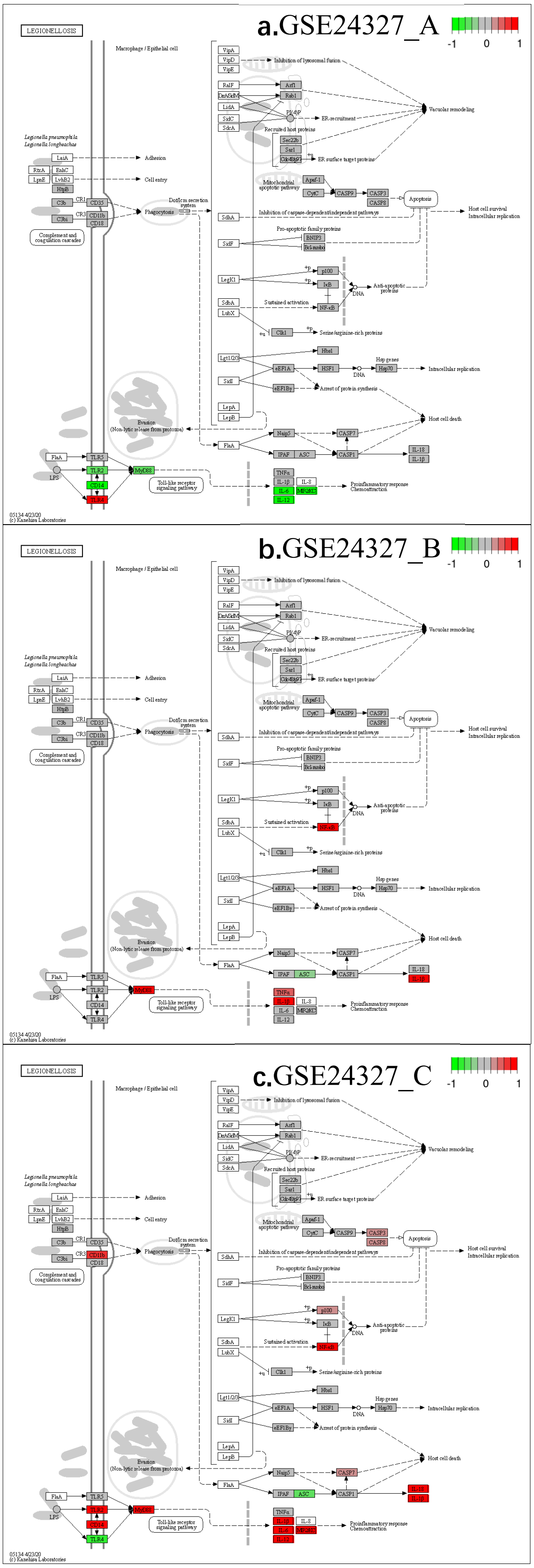

Figure S11 | Comparison on the rendered Legionellosis signaling pathway (mmu05134).

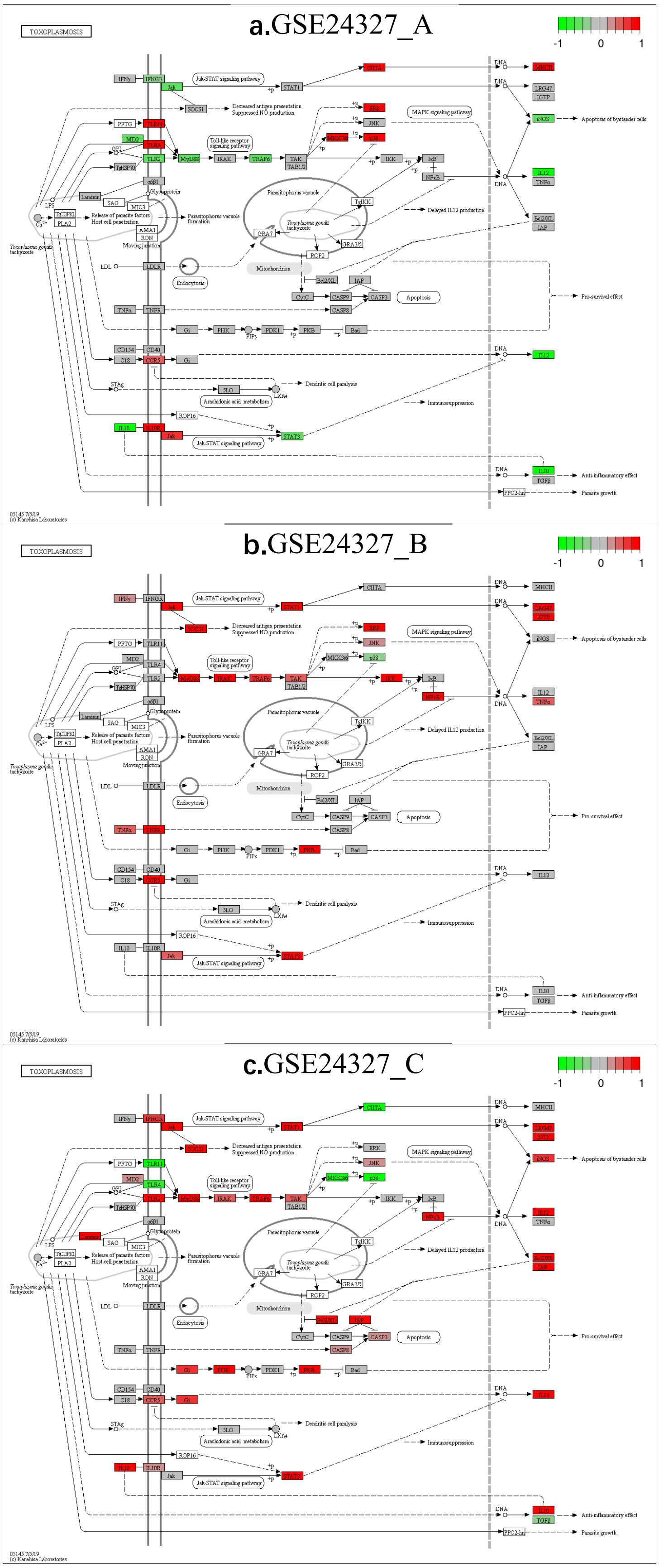

Figure S12 | Comparison on the rendered Toxoplasmosis signaling pathway (mmu05145)

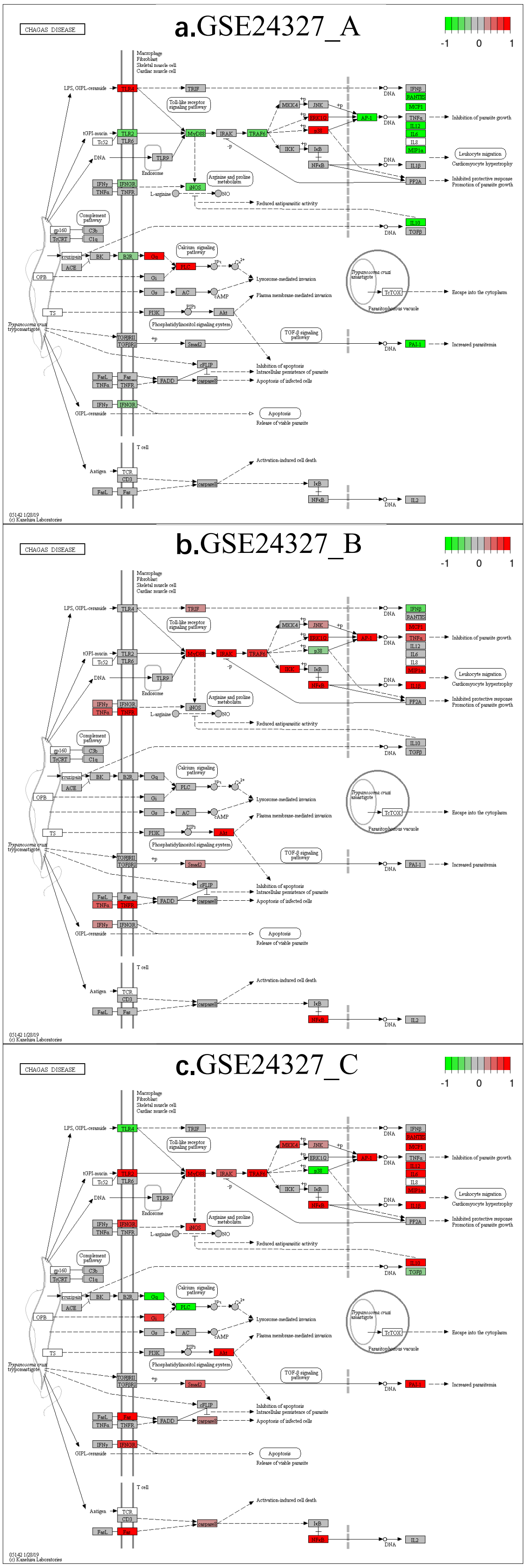

Figure S13 | Comparison on the rendered Chagas disease (American trypanosomiasis) signaling pathway (mmu05142).

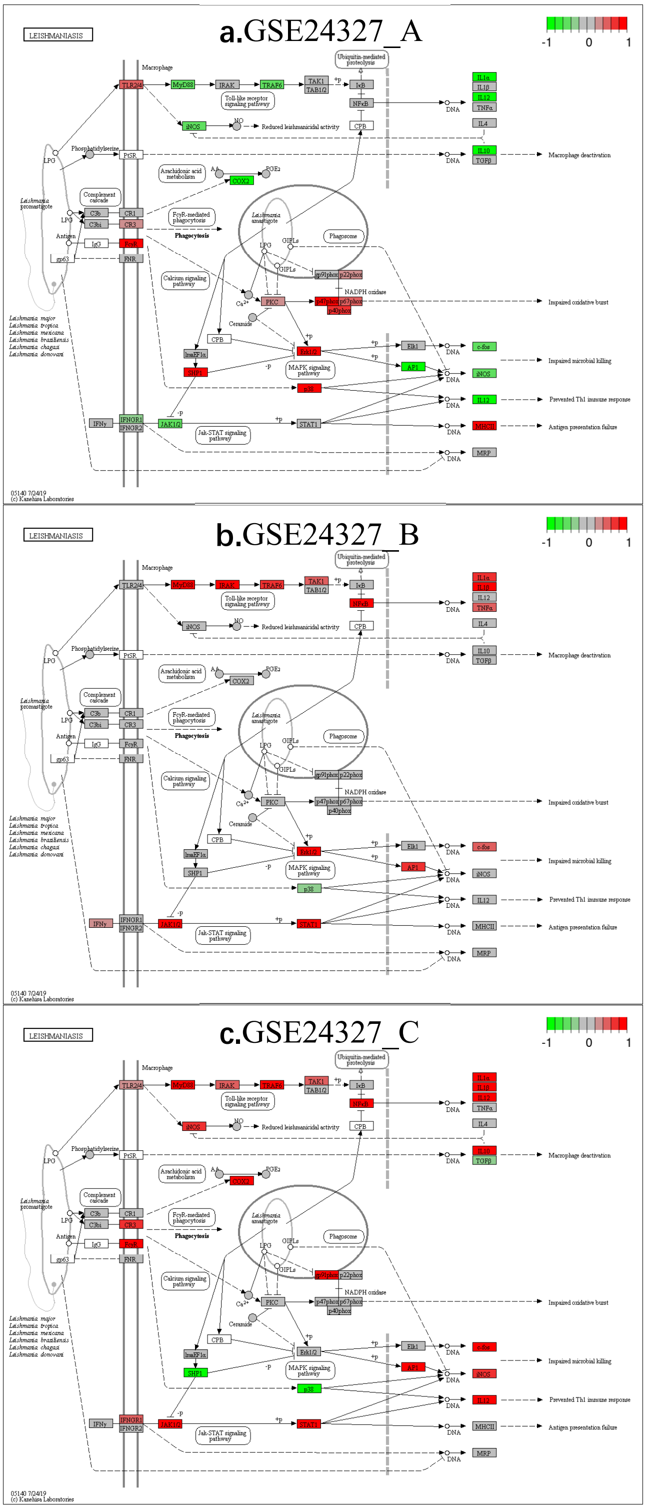

Figure S14 | Comparison on the rendered Leishmaniasis signaling pathway (mmu05140).

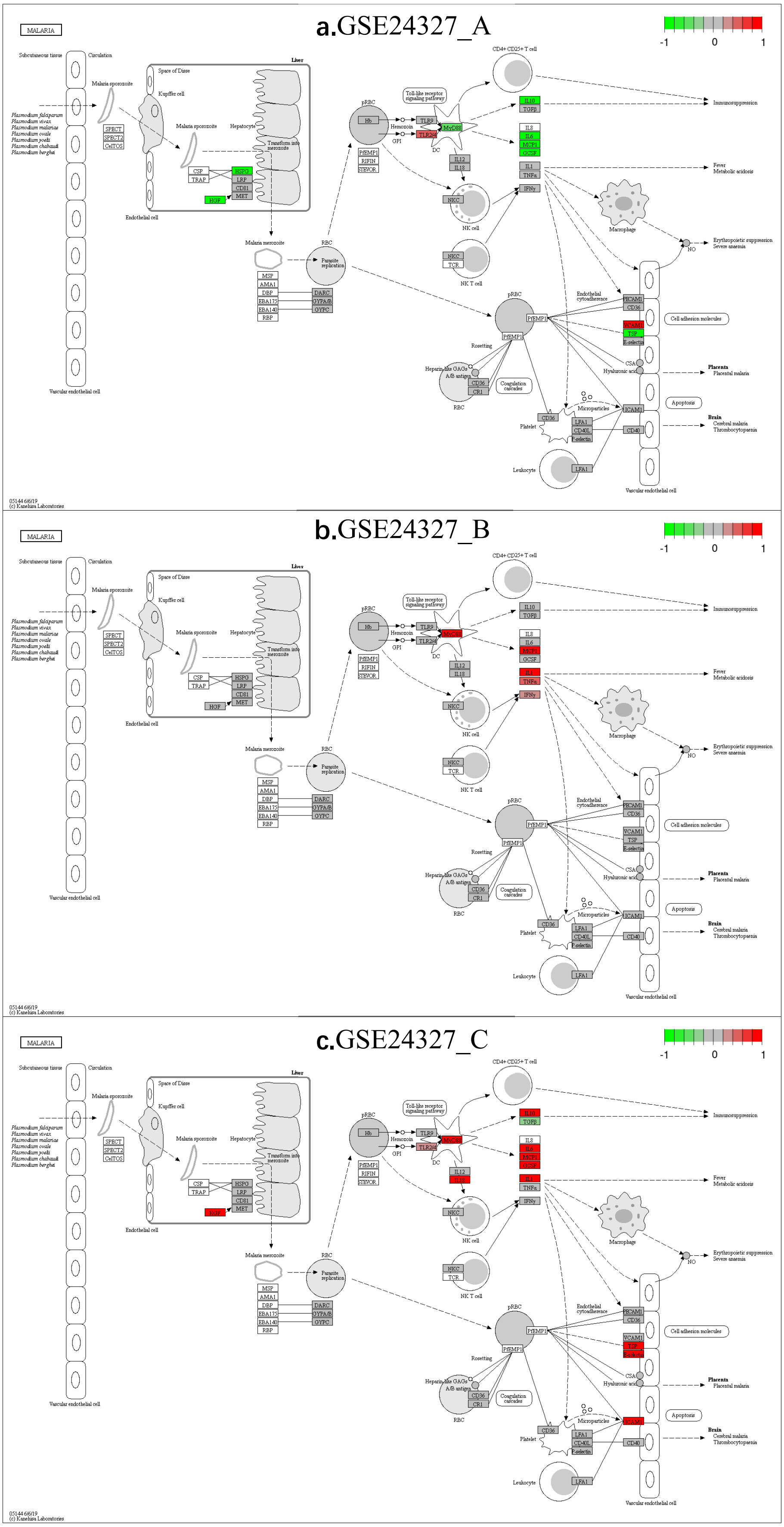

Figure S15 | Comparison on the rendered Malaria signaling pathway (mmu05144)

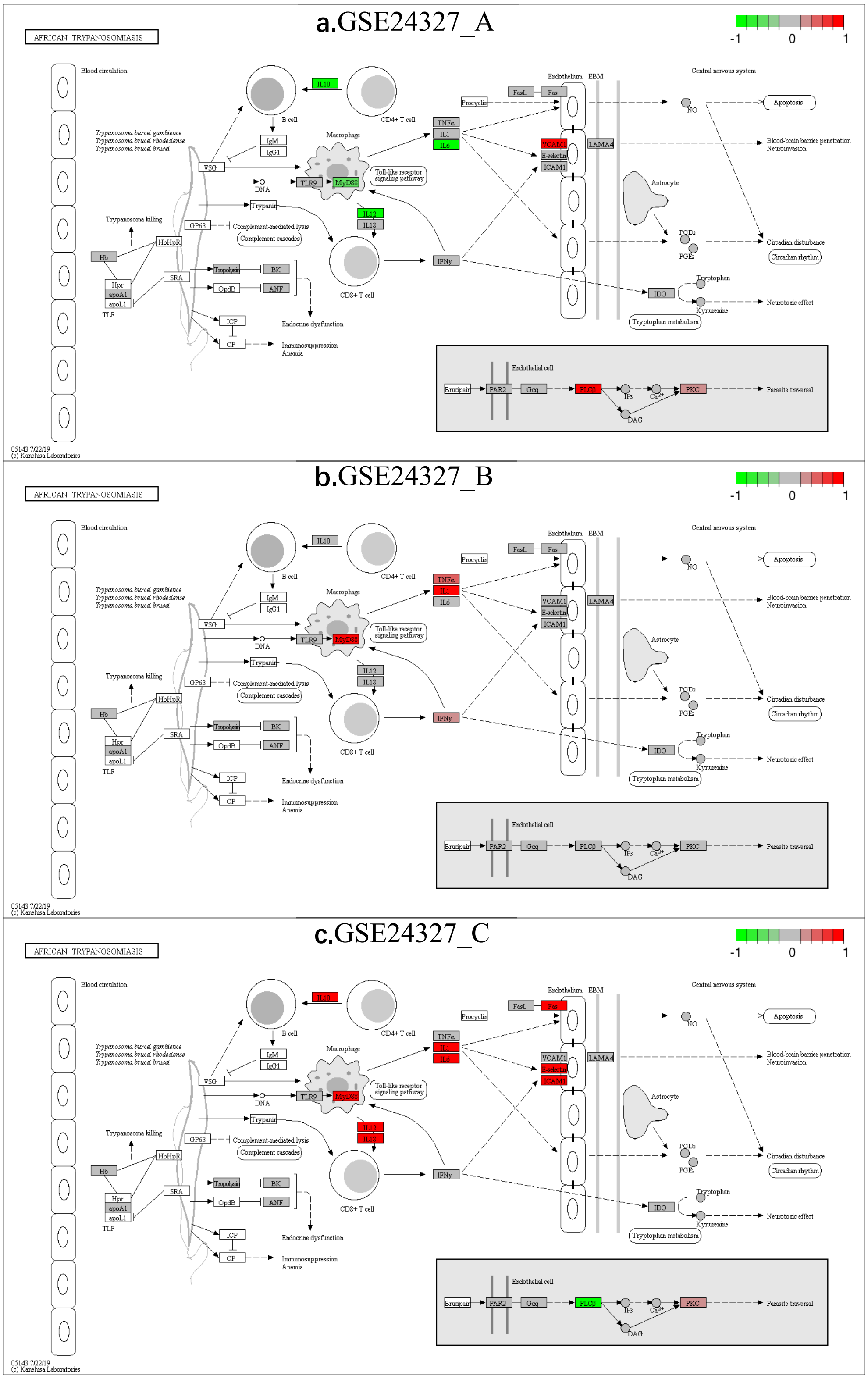

Figure S16 | Comparison on the rendered African trypanosomiasis signaling pathway (mmu05143).

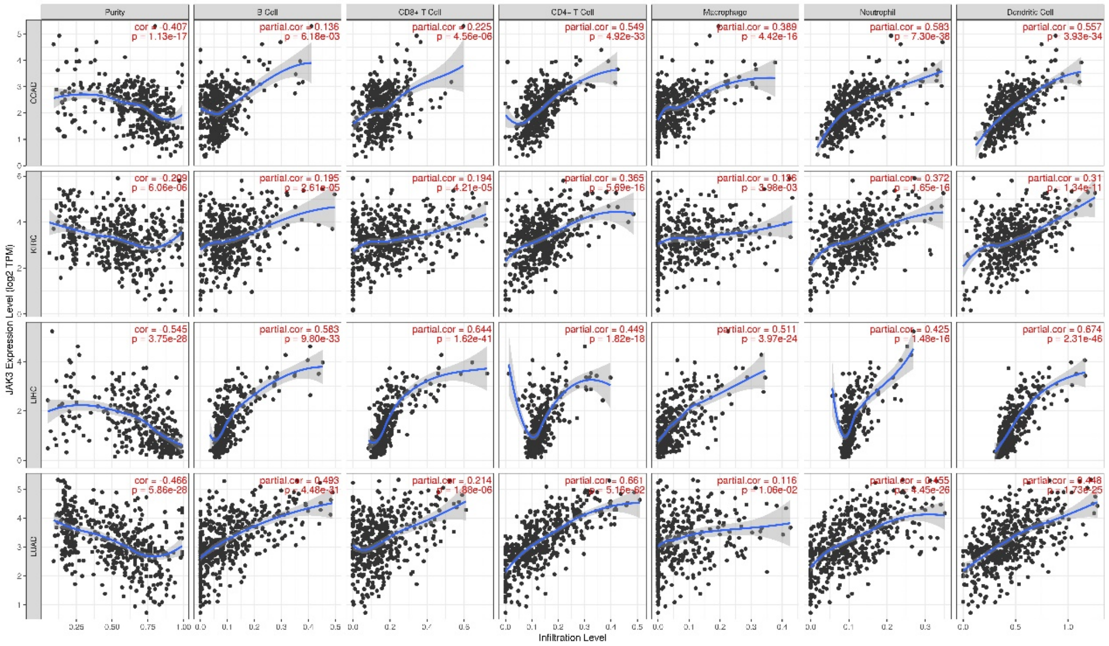

Figure S17 | The positive, significant correlation between the infiltration level of core immune cells (X-axis) and the expression level of key regulator Jak3 (Y-axis). COAD: colon, KIRC: kidney, LIHC: liver, and LUAD: lung. *p*<0.001***.

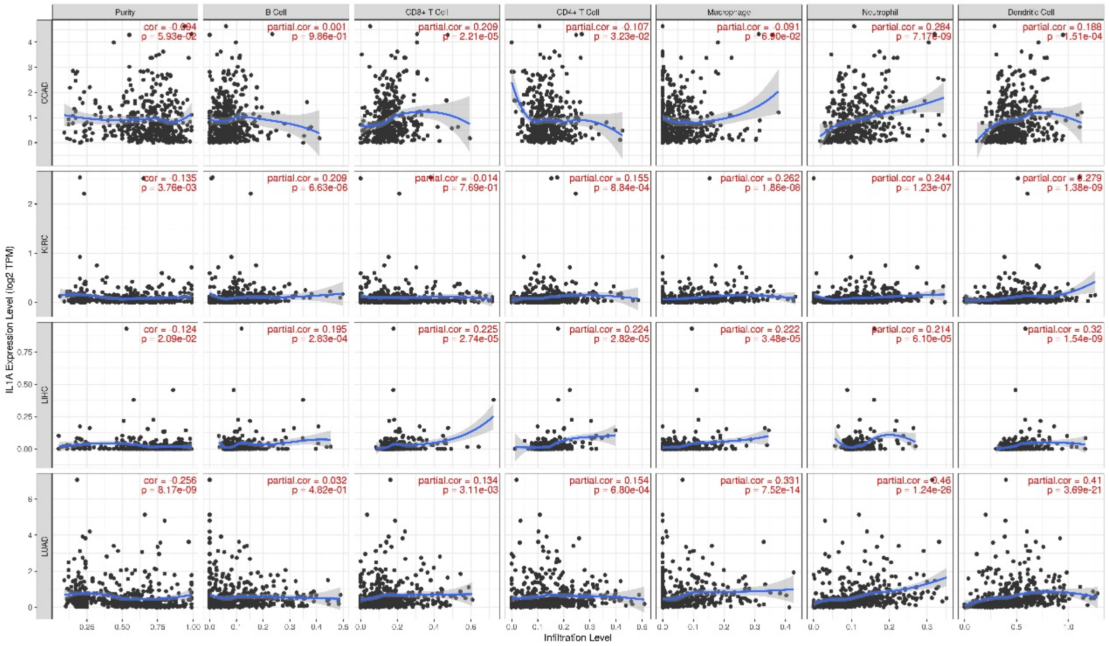

Figure S18 | The positive, significant correlation between the infiltration level of core immune cells (X-axis) and the expression level of key regulator Il1a (Y-axis). COAD: colon, KIRC: kidney, LIHC: liver, and LUAD: lung. *p*<0.001***.

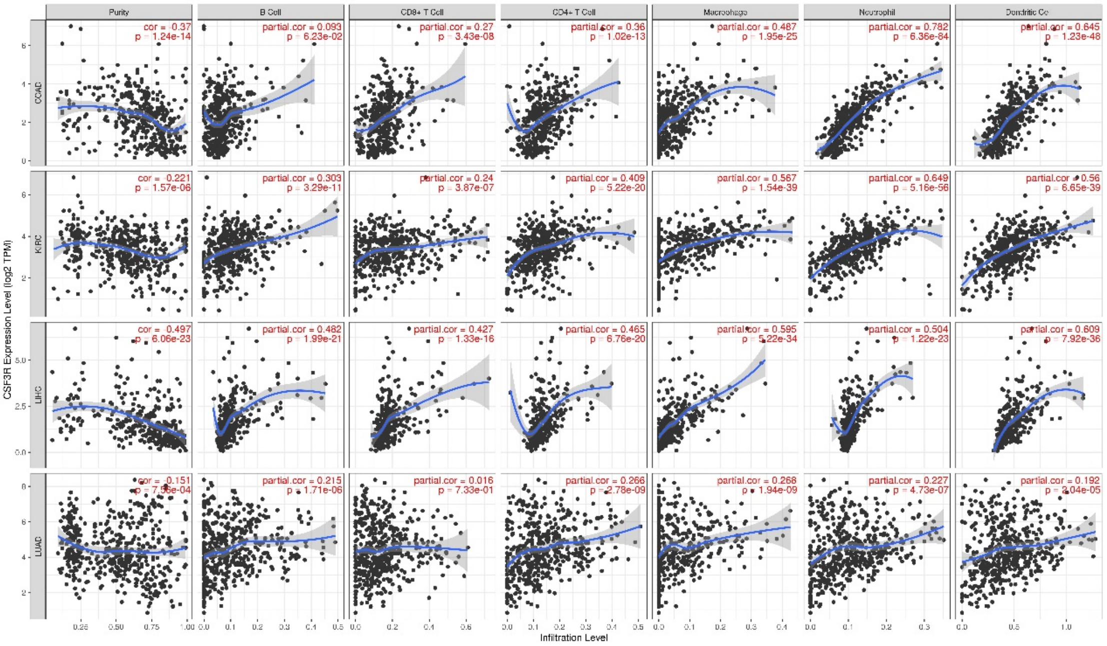

Figure S19 | The positive, significant correlation between the infiltration level of core immune cells (X-axis) and the expression level of key regulator Csf3r (Y-axis). COAD: colon, KIRC: kidney, LIHC: liver, and LUAD: lung. $p<0.001$***.

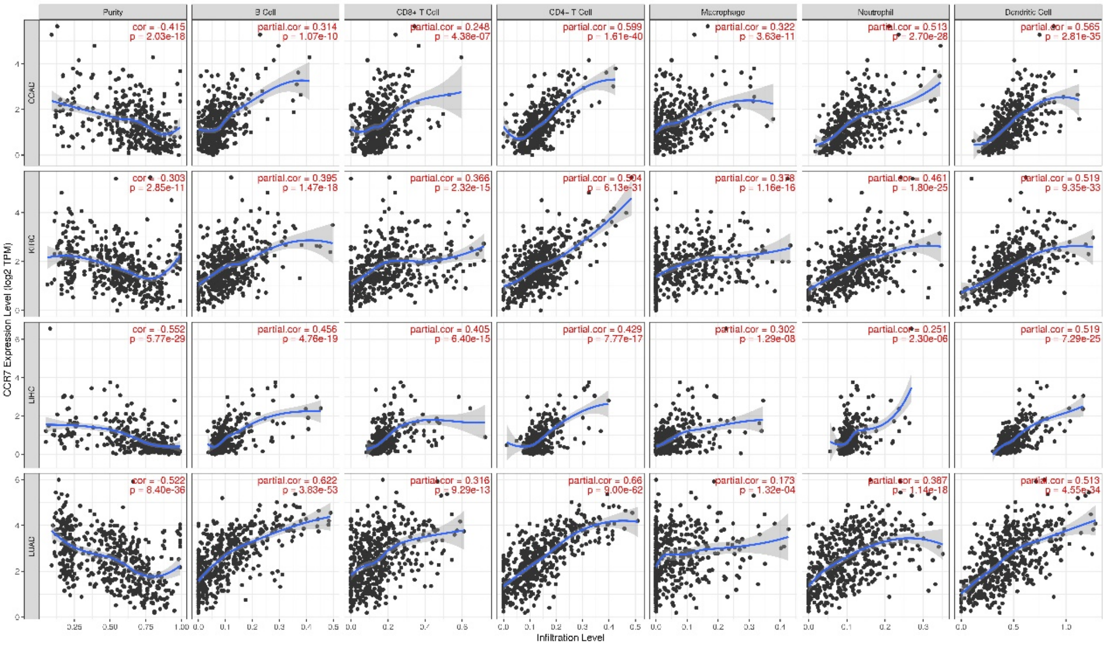

Figure S20 | The positive, significant correlation between the infiltration level of core immune cells (X-axis) and the expression level of key regulator Ccr7 (Y-axis). COAD: colon, KIRC: kidney, LIHC: liver, and LUAD: lung. $p<0.001$***.
